# Cultural Influences, Experiences and Interventions Targeting Self‐Management Behaviours for Prediabetes or Type 2 Diabetes in First‐Generation Immigrants: A Scoping Review

**DOI:** 10.1111/jan.16621

**Published:** 2024-11-21

**Authors:** Min Zhang, Kirsten Coppell, Johnny Lo, Lisa Whitehead

**Affiliations:** ^1^ School of Nursing and Midwifery Edith Cowan University Perth Western Australia Australia; ^2^ The Centre for Evidence‐Informed Nursing, Midwifery and Healthcare Practice, a JBI Affiliated Group Joondalup Western Australia Australia; ^3^ Department of Medicine University of Otago Wellington Wellington New Zealand; ^4^ Nelson Marlborough Institute of Technology Nelson New Zealand; ^5^ School of Science Edith Cowan University Perth Western Australia Australia; ^6^ University of Jordan Amman Jordan; ^7^ University of Otago Dunedin New Zealand

**Keywords:** diabetes, management, multicultural issues, self‐care

## Abstract

**Aim:**

To map the existing evidence and identify research gaps regarding the self‐management of prediabetes or type 2 diabetes among first‐generation immigrants ≥ 18 years.

**Design:**

A scoping review followed the JBI guidelines and was in accordance with the PRISMA extension for Scoping Reviews.

**Methods:**

A systematic search of CINAHL, Cochrane, EMBASE, MEDLINE, PsycINFO, ProQuest, SCOPUS and the Web of Science was conducted. Grey literature and reference lists of included studies were searched for additional citations. Articles published in English from the database inception to February 2023 were included.

**Results:**

We included 96 studies, of which 28.1% were published within the last 5 years. Most studies (71.9%) were conducted in the United States. Study participants were recruited mainly from community settings and English was their second language. The most common study methodologies used were cross‐sectional surveys and phenomenological interviews. Only two studies specifically focussed on individuals with prediabetes. Multiple factors, such as age, gender, country of origin and other societal, linguistic, cultural and resource barriers or facilitators, as well as patient's cultural unique experiences, were of particular significance for self‐management behaviours. Although several studies reported that culturally tailored interventions were feasible and acceptable among first‐generation immigrants living with type 2 diabetes but not prediabetes, the duration and intensity of these interventions varied.

**Conclusion:**

Health professionals should consider various demographic, societal, linguistic and cultural factors, such as participants' low English language proficiency, and provide appropriate support for this group to ensure better self‐management behaviours. Tailoring interventions to individual and cultural preferences in collaboration with key stakeholders is crucial for adult immigrants with prediabetes and type 2 diabetes across diverse cultural and ethnic groups.

**Impact:**

Developing and applying culturally tailored self‐management interventions for the targeted population, particularly those with prediabetes, should be an important direction for future research.

No Patient or Public Contribution.

## 1 Introduction

Diabetes is one of the most prevalent noncommunicable diseases that contributes to increased morbidity, mortality and the cost of illness worldwide (IDF 2021). The global diabetes prevalence among adults increased from 4.7% in 1980 to 10.6% in 2021 (IDF 2021). This figure is projected to increase to 12.2% by 2045, and 94% of these cases are predicted to be in low‐ and middle‐income countries (IDF 2021). The high rate of diabetes imposes a tremendous direct and indirect health and economic burden worldwide (Force et al. 2021). Diabetes can damage the blood vessels, heart, kidneys, nerves and eyes over time (WHO 2023). Alarmingly, diabetes caused 4.2 million deaths globally in 2019, and an estimated 6.7 million deaths were due to diabetes‐related causes in 2021 (IDF 2021; Saeedi et al. 2019). During the last 15 years, global spending on diabetes‐related issues increased more than threefold, reaching USD 966 billion in 2021; and direct health expenditure on diabetes was nearly one trillion USD in 2021, accounting for approximately 10% of global health expenditure (IDF 2021; Sun et al. 2022). The United States spends the most on diabetes (USD 294.6 billion), followed by China (109 billion) (IDF 2021). These statistics underscore the importance of delaying and managing diabetes in low‐, middle‐ and high‐income countries.

Lifestyle modifications, such as consuming a healthy diet, increasing physical activity and weight loss, are recommended as first‐line management approaches for prediabetes and type 2 diabetes (Echouffo‐Tcheugui et al. 2023; Fazli et al. 2020; Jonas et al. 2021; WHO 2023). However, making and maintaining lifestyle changes can be challenging due to multiple factors, including health literacy, knowledge, finances, health beliefs, behaviours related to cultural and religious beliefs and disease‐related factors (AGDH 2021; Bell et al. 2020; Gupta et al. 2019). There is good evidence that patient self‐management and engagement are key to making and maintaining lifestyle changes to reduce the burden of diabetes and its complications (AGDH 2021). Awareness and insight into self‐management behaviours can inform prevention and management programmes.

### 1.1 Background

“Immigrants” are defined as people who move to a country other than that of their country of birth or usual residence, resulting in the country of destination becoming their new country of residence (Douglas, Cetron, and Spiegel 2019). Approximately 4% of the global population was classified as immigrants in 2020 (Batalova 2022). Immigrants living with illness and disease are more likely to face significant health disparities, and thus, health providers need to understand how to address their needs (AGDH 2021; AIHW 2005). From the “generational status” perspective, an individual's or their parents' place of birth can define immigrants as first‐generation, second‐generation, or third‐and‐higher‐generation immigrants (USCB 2021). ‘First‐generation’ typically refers to those who are foreign‐born (i.e., a person born in a country other than their country of residence) (ABS 2021; USCB 2021). First‐generation people speak the language and hold many cultural values of their country of origin. The terms' first‐generation’ and ‘foreign‐born’ are used interchangeably in the literature.

Adult immigrants are at high risk of developing prediabetes and type 2 diabetes (McKay et al. 2022). Type 2 diabetes is the most common form of diabetes, accounting for 90%–95% of all diabetes worldwide. This form encompasses individuals who generally have relative (rather than absolute) insulin deficiency and peripheral insulin resistance (i.e., a decreased biological response to insulin) (ADA 2024b). Prediabetes, as the precursor of type 2 diabetes, is a condition with blood glucose levels higher than normal but not high enough to be diagnosed with diabetes and collectively includes impaired fasting glucose (IFG), impaired glucose tolerance (IGT) and elevated glycated haemoglobin (HbA1c) (Jonas et al. 2021). Compared to native‐born residents, first‐generation immigrants have a higher prevalence of diabetes and higher diabetes‐related mortality rates, and they face more complex obstacles to managing their diabetes, thereby resulting in poorer health behaviours and diabetes outcomes (McKay et al. 2022; Montesi, Caletti, and Marchesini 2016). This could be due to language barriers and limited health literacy, leading to difficulties with accessing the healthcare they need, shifts in socioeconomic status, decreased physical activity and dietary changes (Abubakar et al. 2018).

Identifying, implementing and maintaining effective self‐management are important for managing both prediabetes and type 2 diabetes. Self‐management is the process of an individual actively participating, engaging and being involved in self‐care activities with the aim of improving health behaviours and well‐being (Lambrinou, Hansen, and Beulens 2019). Instead of “adherence” or “compliance”, diabetes self‐management focuses on individuals' responsibility and role in improving their health outcomes and efficiency of care (Captieux et al. 2018; van Smoorenburg et al. 2019). Information on the self‐management practices of first‐generation immigrants living with prediabetes or type 2 diabetes is disparate, and this has not previously been synthesised in a review.

A scoping review methodology was selected according to Pollock et al. (2021) decision tree for selecting a scoping review methodology. After searching the Cochrane Database of Systematic Reviews, JBI Evidence Synthesis, MEDLINE, and PROSPERO, no synthesis of evidence or ongoing review protocol that determined what is known about prediabetes or type 2 diabetes self‐management behaviours among first‐generation immigrants was found. A scoping review was undertaken to map the key concepts and identify gaps in knowledge on this topic.

## 2 Methods

### 2.1 Review Design

This scoping review was conducted following the JBI guidance for scoping reviews (Peters et al. 2020, 2021). A priori protocol was registered in the Open Science Framework and can be accessed via https://osf.io/jw4h7.

#### 2.1.1 Eligibility Criteria

This review considered studies that met the following eligibility criteria:

1. Population: Studies that included adults aged 18 years and over who identified as first‐generation immigrants and had diagnosed prediabetes or type 2 diabetes. Individuals characterised as ‘first‐generation immigrants’ included in this review were immigrants who were born overseas, including foreign‐born temporary or permanent residents, refugees, asylees and unauthorised migrants (ABS 2021; Douglas, Cetron, and Spiegel 2019).
2. Concept: Studies that explored self‐management related to prediabetes or type 2 diabetes among first‐generation immigrants. In this review, ‘self‐management’ refers to a set of skilled behaviours (e.g., exercise, diet, blood glucose monitoring) that were actively engaged in to manage a patient's own disease in partnership with their healthcare provider (Goodall and Halford 1991; Wang et al. 2023).
3. Context: Studies conducted in any country or healthcare setting were considered.
4. Types of sources: Peer‐reviewed publications and grey literature (e.g., theses and dissertations) were included. Studies using qualitative, quantitative and mixed‐methods study designs were considered for inclusion. Studies were excluded if they did not focus on self‐management interventions; did not include first‐generation immigrants; did not specifically include participants with prediabetes or type 2 diabetes; were conference abstracts or protocols or the full text was non‐English.

### 2.2 Search Methods

#### 2.2.1 Search Strategy

Collaborating with an experienced librarian, the research team developed a logic grid and a full search strategy (MEDLINE) based on the population‐concept‐context and the review question. Both keywords and index terms were used to create robust and sensitive searches. Truncation, AND, OR and NOT Boolean searches were performed to enhance specificity and sensitivity. The search strategy was adapted for each included information source (Table S1).

A three‐step search process was used for the search strategy. The initial search was a systematic search of seven databases, including the CINAHL (EBSCO), Cochrane Library, EMBASE (Elsevier), MEDLINE (EBSCO), PsycINFO (Ovid), SCOPUS and Web of Science. The second search was a grey literature search from the WorldCat website, ProQuest, Open Grey, WHO International Clinical Trials Registry organisation and Google Scholar. The third search was a citation search via a review of the reference lists of all studies included. Articles published in English from database inception to February 2023 were included.

#### 2.2.2 Study Selection

A total of 1258 records were identified through the three‐step search, with 820 from the initial search, 320 from grey literature and 118 from the citation search (Figure S1). All identified records were exported into Endnote software version 20.0 (Clarivate, Philadelphia, USA) to remove duplicates. The remaining records were uploaded and collated in the JBI System for the Unified Management of the Assessment and Review of Information (Munn et al. 2019). Two independent reviewers screened the title and abstract, resulting in 226 eligible full‐text records. We resolved disagreements by discussing with a third reviewer to ensure consistency. Two reviewers independently screened the full‐text records, and a further 130 were excluded, with reasons being recorded, leaving a total of 96 studies included in the review (Table S2).

### 2.3 Data Extraction

A standard, pretested data extraction form, based on the JBI data extraction instrument template and our review aim, was used for data extraction (Appendix S1) (Peters et al. 2020). The data extracted included demographic details of participants (e.g., age, sex, ethnicity), migration‐specific factors (e.g., country of origin, duration of immigration), self‐management (e.g., content, barriers/facilitators), context and main study findings. Any disagreements about data extracted were discussed between two authors and, if necessary, with a third author until a consensus was reached. The authors of 12 articles were contacted for missing information.

### 2.4 Data Analysis and Presentation

We used a descriptive, inductive approach to analyse the extracted data. A descriptive statistical analysis was performed using Microsoft Excel (Redmond, Washington, USA) to map study characteristics, frequency of terms used to describe the factors influencing self‐management, self‐management intervention characteristics and frequency of different outcomes and indicators of self‐management behaviours. An iterative process was used to develop categories and subcategories for the factors associated with self‐management behaviours, barriers/facilitators of effective self‐management and the themes of culturally unique experiences. Information about influencing factors was first grouped broadly under main conceptual categories as barriers, facilitators or associated factors, then subsequently recategorised if the factor was culture factors. For example, ‘life after immigration’ was initially extracted as a barrier, then subsequently categorised as a ‘cultural barrier’ because it reflected a ‘cultural influence’ and specifically, the difference in lifestyle and the environment between the country of birth and their new country. Narrative and table formats, figures and bar charts were used to summarise and present the findings.

## 3 Results

### 3.1 Characteristics of Included Studies

The characteristics of the 96 included studies are summarised (Table S3). They were published between 2001 and 2023, with 27 studies (28.1%) published within the last 5 years. The sample size of each study ranged from 5 to 701 participants, and nearly one‐third had a sample size of 60 or more participants. The most common sampling method was convenience sampling (28 studies, 29.2%), followed by purposive sampling, snowballing, theoretical sampling and random sampling.

#### 3.1.1 Population

Participants in the 96 studies ranged from 18 to 95 years old. A total of 56 studies (58.3%) enrolled participants aged > 70 years, and 92 studies (95.8%) reported gender/sex, of which 71 studies reported ≥ 50% of the participants were female. There were six female‐only studies and two male‐only studies. Of the 96 studies, 86 (89.5%) focussed on type 2 diabetes, two studies (2.1%) focussed on persons with type 2 diabetes who were also living with depression, six studies (6.25%) included both persons with prediabetes or type 2 diabetes and two studies (2.1%) reported on individuals with prediabetes only. Sixty‐five studies (67.7%) reported the duration of prediabetes or type 2 diabetes, which ranged from 3 to 22.6 years.

#### 3.1.2 Countries, Settings and Language

**FIGURE 1:**
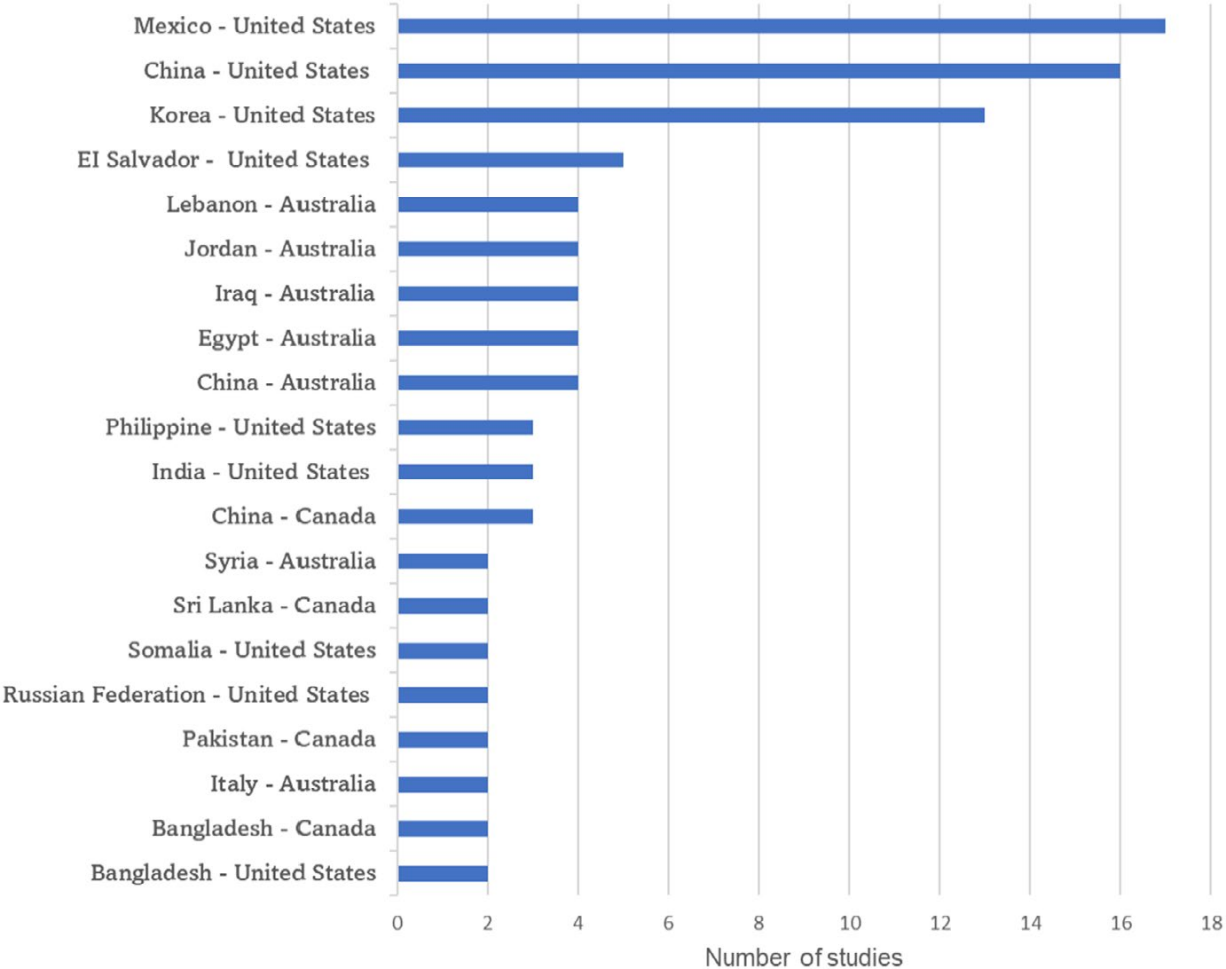
Top 20 country‐to‐country corridors for first‐generation immigrants of all 96 studies. The horizontal axis displays country‐to‐country corridors, representing the movement of immigrants from their country of origin (left) to their country of residence (right) and the vertical axis shows the corresponding number of studies.

Most studies were conducted in the US (*n* = 69), followed by Australia (*n* = 11), Europe (*n* = 11), including Norway, Sweden, Belgium, Denmark, Germany, Italy, Ireland, the Netherlands and the United Kingdom and Canada (*n* = 5). Among the first‐generation immigrants included in the studies, the most common country‐to‐country corridors, representing the movement of immigration from their country of origin to their country of residence, was from Mexico to the US (*n* = 17), then from China (*n* = 16), Korea (*n* = 13) and El Salvador (*n* = 5) to the US, respectively (Figure 1).

Participants were mostly recruited from community settings, such as multiple community settings/institutions (*n* = 53) and community health centres (*n* = 25). Other settings included medical practices or clinics (14 studies), hospitals (*n* = 10), primary care practices (*n* = 6), outpatient services (*n* = 4), diabetes centres (*n* = 3) and senior care facilities (*n* = 1). Two studies did not report specific settings (Jordan and Jordan 2010, 2011). English was the most common second language among first‐generation immigrants, as reported in 87 studies (90.6%). Only 27 studies (28.1%) reported participants' proficiency in a second language, including limited English‐speaking fluency (*n* = 23), limited skills in Danish‐speaking (*n* = 1), Dutch‐speaking (*n* = 1) and satisfactory language skills in Norwegian (*n* = 1).

#### 3.1.3 Types of Study Design

The types and numbers of each study design are listed in Table 1. Thirty‐five (36.5%) studies used quantitative methods, 43 (44.8%) used qualitative methods, and 18 (18.8%) used mixed methods. Most studies employed a qualitative design (*n* = 32, 33.3%), followed by a cross‐sectional quantitative design (*n* = 18, 18.75%). The two most frequently used mixed‐methods methodologies were convergent parallel and explanatory sequential designs. No exploratory sequential design was reported.

**TABLE 1:** Distribution of include studies by study design (*N* = 96).

Types of study design	Number of studies	Percentage
Quantitative (*n* = 35)		
Cross‐sectional	18	18.75%
Quasi‐experimental (pre‐post)	9	9.40%
Randomised clinical trials	5	5.20%
Cohort	3	3.10%
Qualitative (*n* = 43)		
Phenomenology	32	33.30%
Grounded theory	3	3.10%
Ethnographies	3	3.10%
Case studies	2	2.10%
Narrative inquiry	2	2.10%
Secondary analysis	1	1.00%
Mixed methods (*n* = 18)		
Convergent	8	8.30%
Explanatory (QUAN → QUAL)	8	8.30%
Exploratory (QUAL → QUAN)	0	0%
Triangulation	2	2.10%

*Notes:* → A sequential approach to data collection; one form builds on or connects with the other. QUAN indicates quantitative methods, and QUAL indicates qualitative methods.

#### 3.1.4 Conceptual/Theoretical Framework Used

Forty‐six (47.9%) of the 96 studies mentioned conceptual or theoretical frameworks, including eight studies which used more than one theory or model. The most used framework was social cognitive theory (SCT), reported in seven studies.

### 3.2 Main Results of the Included Evidence

#### 3.2.1 Factors Influencing Self‐Management

**FIGURE 2:**
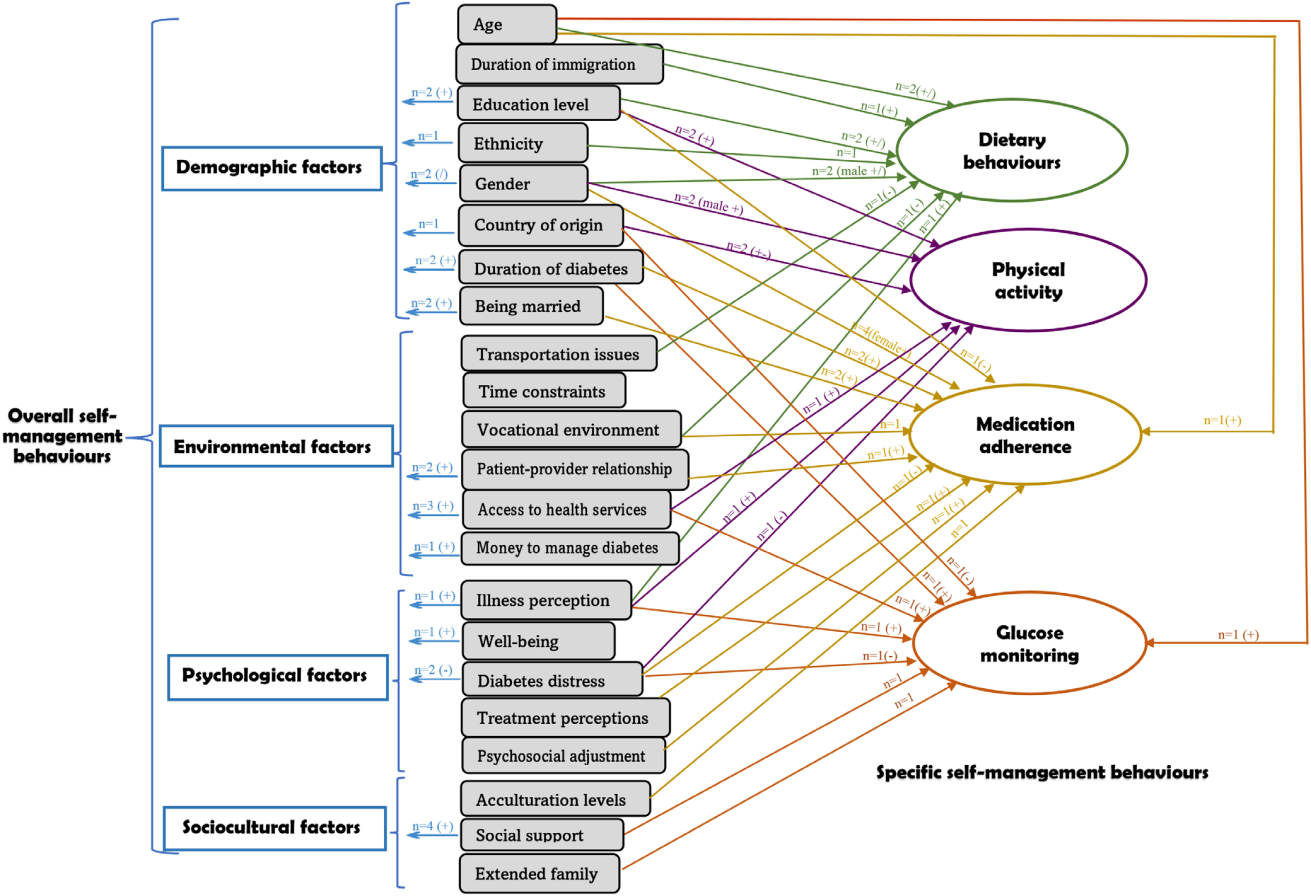
Associated factors of self‐management behaviours among first‐generation immigrants living with type 2 diabetes. (+) positive correlation, (−) negative correlation, (/) not significant, ⟶ an association between factor and overall or specific self‐management behaviours.

This section summarises the factors that influenced participants' self‐management behaviours based on the quantitative and qualitative data extraction. These factors were separated into two sections: associations from the quantitative data; and barriers and facilitators from the qualitative data. We identified four categories of factors associated with type 2 diabetes self‐management, in decreasing order of frequency: demographic, psychological, sociocultural and environmental factors (Figure 2).

##### 3.2.1.1 Factors Associated With Type 2 Diabetes Self‐Management: Summary of Quantitative Component Findings

In total, 22 types of associated factors were identified from the quantitative findings of 18 studies (16 quantitative and two mixed‐methods), and these mostly addressed overall or specific self‐management behaviours. The first‐generation immigrants in these studies were from either a single ethnic group or multiethnic groups. The former included Mexicans, Latinos, Hispanics, Black Caribbeans, Filipinos, Irish, Caucasians, Koreans, and the most common group, Chinese (Table S3). The multiethnic study groups included individuals from various ethnic backgrounds, such as a sample of Asian immigrants, including Sri Lankan, Bangladeshi, Pakistani and Chinese (Hyman et al. 2017).

Twenty studies used a self‐management assessment questionnaire or scale to assess the overall level of self‐management behaviours (Table S5). Ten studies reported the relationship between the overall level of self‐management behaviours and multiple factors. Figure 2 also shows the specific self‐management behaviours, which were grouped into four categories: dietary (*n* = 3) (Ho et al. 2020; Jordan and Jordan 2010; Mier et al. 2012), physical activity (*n* = 3) (Hyman et al. 2017; Jordan and Jordan 2010; Mier et al. 2012), medication adherence (*n* = 5) (Alzubaidi et al. 2015; Chesla et al. 2014; Eh et al. 2016; Jordan and Jordan 2010; Williams 2016) and glucose monitoring (*n* = 6) (Chesla et al. 2014; Choi, Toyama, and Brecht 2020; Choi 2009; Hyman et al. 2017; Jordan and Jordan 2010; Mier et al. 2012).

The following factors were found to be associated with the overall level of self‐management behaviours: education level (Eh et al. 2016; Pettersson et al. 2023), ethnicity (Vaccaro et al. 2014), gender (Hyman et al. 2017), country of origin (Pettersson et al. 2023), duration of diabetes (Eh et al. 2016; Pettersson et al. 2023), being married/living with someone, illness perception (Alzubaidi et al. 2015; Park 2020), well‐being (Pettersson et al. 2023), diabetes distress (Alzubaidi et al. 2022; Huang, Zuñiga, and García 2022), social support (Choi, Toyama, and Brecht 2020; Vaccaro et al. 2014; Williams 2016), patient–provider relationship (Cha et al. 2012; Hyman et al. 2017; Pettersson et al. 2023) and money to manage diabetes (Figure 4). For example, positive illness perceptions were significantly associated with better adherence to diabetes self‐management (Alzubaidi et al. 2015; Park 2020).

Medication adherence was the most reported specific behaviour, followed by behaviours related to glucose monitoring, physical activity and diet. Increasing age (Jordan and Jordan 2010), being female (Chesla et al. 2014; Williams 2016), long duration of immigration, being married (Chesla et al. 2014; Williams 2016), psychosocial adjustment, presence of extended family, acculturation levels, positive patient–provider relationship (Cha et al. 2012; Pettersson et al. 2023) and good treatment perceptions (Alzubaidi et al. 2015) were positively associated with medication adherence, while low education level, diabetes distress and vocational environment had a negative influence on medication adherence.

Among all categories, gender (male or female) was the most frequently mentioned factor affecting overall and specific self‐management behaviours. However, the direction of the association between male/female and self‐management was inconsistent across studies. Some studies reported that males were more likely to have healthy diets (Mier et al. 2012), while others showed that there was no significant gender difference in following a healthy eating plan (Jordan and Jordan 2010) or maintaining self‐care (Pettersson et al. 2023). Among Mexico‐born immigrants in the US, being female was positively associated with physical activity (Mier et al. 2012); whereas among Filipino‐American adults, females Vaccaro et al. (2014) reported no gender differences in type 2 diabetes self‐management among Haitian Americans and African Americans.

Education level was the second most frequently mentioned factor. Studies showed that people who had a higher education level are more likely to be physically active (Jordan and Jordan 2010; Mier et al. 2012), have healthier diets (Jordan and Jordan 2010) and engage in self‐management behaviours (Eh et al. 2016; Pettersson et al. 2023). Social support (e.g., spousal/family/friends) (Alzubaidi et al. 2022; Choi, Toyama, and Brecht 2020; Vaccaro et al. 2014; Williams 2016) was identified as the third most common factor, and this was associated with behaviours related to self‐management and glucose monitoring. In addition, acculturation levels, including lifestyle changes after immigration, and ease of diabetes management in the resident country, can also impact behaviours related to medication adherence (Eh et al. 2016) and glycaemic control (Venkatesh et al. 2013).

##### 3.2.1.2 Factors Acting as Barriers to, or Facilitators of Effective Self‐Management: Summary of Qualitative Component Findings

**FIGURE 3:**
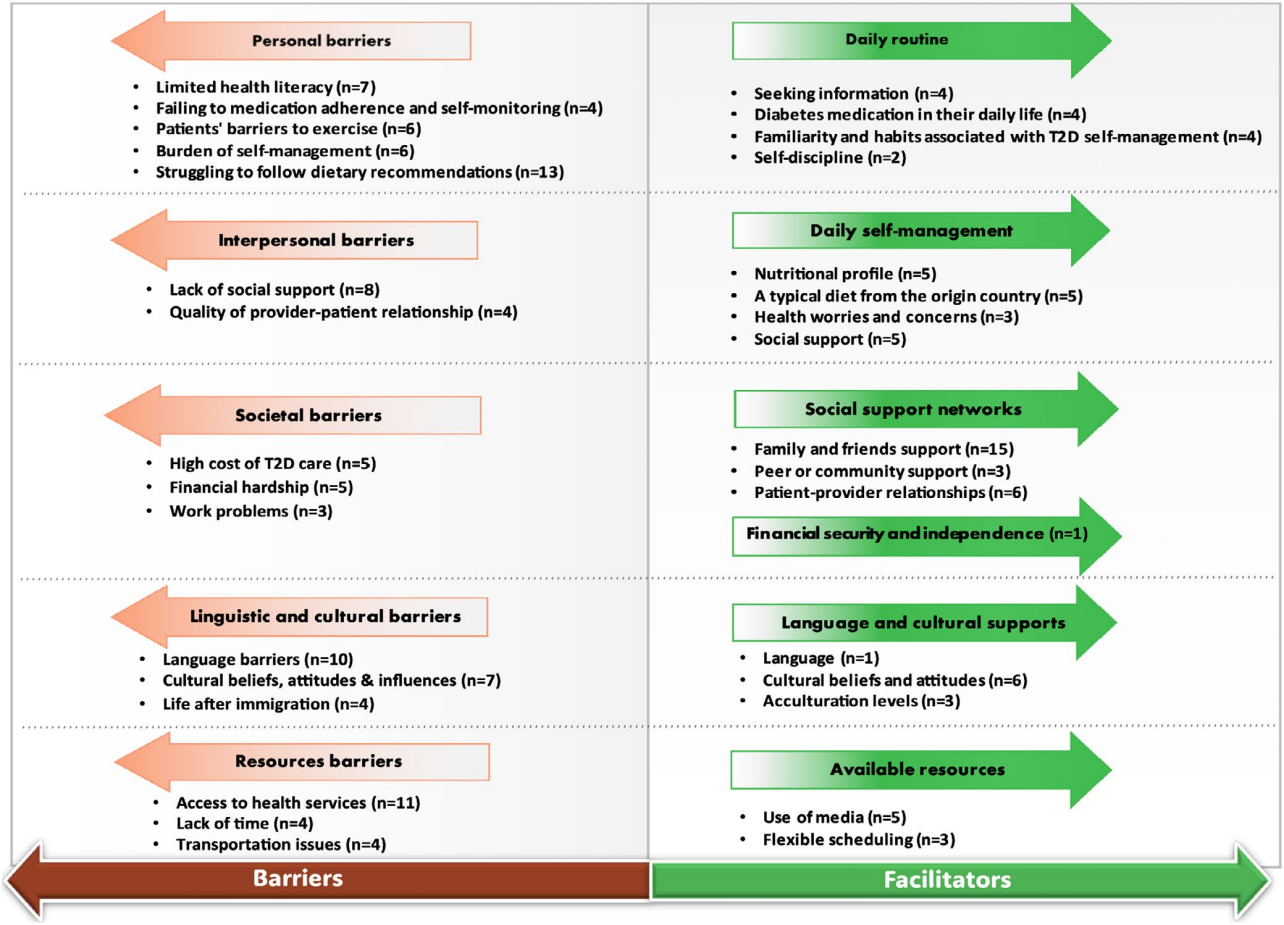
Barriers to and facilitators of effective self‐management. *N*, the number of studies involving a particular facilitator or barrier; T2D, type 2 diabetes.

Thirty‐eight studies (31 qualitative and seven mixed methods) identified barriers to, or facilitators of, effective self‐management, which are summarised in Figure 3. Most studies focussed on first‐generation American immigrants from specific ethnic groups. Chinese Americans and Korean Americans were the most frequent groups studied, followed by Latino, Filipino, Haitian, Bangladeshi, African, Asian‐Indian, Hispanic, Middle Eastern, Hmong, Sub‐Saharan African and Russian‐speaking Slavic immigrants. Additionally, other studies included also encompassed immigrant groups living in Australia (Sudanese, Maltese, Italian), Norway (Kurdish, Pakistani) and Italy (North African, Bangladeshi).

###### 3.2.1.2.1 Barriers Identified as Impeding Effective Self‐Management Behaviours

Identified barriers were divided into five domains, including personal (*n* = 2), interpersonal (*n* = 10), societal (*n* = 9), linguistic and cultural (*n* = 17) and resources (*n* = 13). Within each domain, subcategories were identified (Figure 3, Table S4). Participants' personal barriers involved five themes, including health literacy, medication adherence and self‐monitoring, exercise, the burden of self‐management and diet. Eight studies reported that limited health literacy was an obstacle to effective self‐management. In addition, participants had knowledge gaps and misinformation regarding type 2 diabetes. They assumed that taking insulin may result in foot amputation and considered that the information about diabetes was too scientific to understand (Njeru et al. 2015; Smith‐Miller, Berry, and Miller 2017).

‘Struggling to follow dietary recommendations’ was the most commonly described theme in the domain of personal barriers (*n* = 14). Participants reported that they faced many challenges when trying to adopt a healthy diet, such as the traditional views of food, the expense associated with healthier foods, or their reliance on their spouse or family members to purchase and prepare food (Kaltman et al. 2015; Shultz, Corbett, and Allen 2009). For example, a participant in one study stated that her family's food choices were only pre‐packaged and canned foods (Smith‐Miller, Berry, and Miller 2017). A participant in another study described that “Within my family it is inconvenient for them to just cook for me and then also cook for the rest of the family” (Njeru et al. 2015).

Ten studies reported interpersonal barriers, which consisted of two themes: ‘Lack of social support’ (*n* = 8) and ‘Quality of the provider–patient relationship’ (*n* = 4). For example, studies indicated that because participants could not meet their relatives, friends, or family during the COVID‐19 pandemic, isolation had negatively influenced their type 2 diabetes self‐management practices (Mier et al. 2012). Nine of the included studies reported societal barriers, such as the high cost of diabetes care (*n* = 5), financial hardship (*n* = 5) and work‐related problems (*n* = 3). As one participant stated: ‘My nutrition is, it is not that great because when one is poor, and one does not have money to buy good things’ (Wieland et al. 2017).

Language barriers were noted in ten studies as one of the major barriers among first‐generation immigrants. The theme ‘Cultural beliefs, attitudes and influences’ was reported in seven studies. One participant reported cultural disconnection with providers: ‘I don't tell my doctor about everything that I eat, because I don't think they'd understand what our traditional diet is like’ (Barbara and Krass 2013). Four studies mentioned that the differences in the way of life after immigration can impede effective self‐management behaviours. The most common resource barrier identified was limited access to health services (*n* = 11), including inadequate diabetes education, health insurance and community‐level healthcare resources. Other resource factors included a lack of time (*n* = 4) and limited access to transportation (*n* = 4).

###### 3.2.1.2.2 Facilitators Identified as Promoting Effective Self‐Management Behaviours

Six domains were identified as facilitators of effective self‐management behaviours: daily routine (*n* = 9), dietary self‐management (*n* = 7), social support networks (*n* = 13), financial independence (*n* = 1), language and cultural supports (*n* = 10) and available resources (*n* = 7). (Figure 3).

Having a daily routine was considered an important facilitator of self‐management behaviours, including medication adherence, physical activity and self‐monitoring. Four studies reported that ‘seeking information’ (e.g., easy access to health information and sources providing participants with diabetes information) was beneficial for maintaining a healthy daily routine, thus facilitating self‐management behaviours. One participant mentioned how he accepted and coped with diabetes – “Truthfully after the shock, I started to accept it and became friends with it…If it's high, I bring it down, if it's down, I pick it up. I deal with it as it is (Joo and Lee 2016). Besides, other themes such as ‘Diabetes medication in their daily life’, ‘Familiarity and habits associated with T2D management’ and ‘Self‐discipline’” were also facilitators identified as promoting effective self‐management behaviours.

Dietary self‐management consisted of four themes, including ‘Nutritional profile’, which involved nutritional counselling and personalised diets; ‘A typical diet from the origin country’; Patients' ‘Health worries and concerns’; and ‘Social support’ from family, friends or health workers who speak the same language as the patient. As Shultz, Corbett, and Allen (2009) mentioned in their study, health worries and concerns can also act as motivation for a healthy diet. This is because the participants ‘prefer to eat better than be sick’ or hold the view such as ‘if I eat certain things (e.g., sweets, fatty meats), I'm going to feel bad’ (Shultz, Corbett, and Allen 2009).

Strong social support networks involving a spouse or partner, family, friends, peers, community and healthcare providers can benefit effective self‐management behaviours (*n* = 15). One patient said, ‘My daughter takes me to the doctors and makes sure I understand them’ (Barbara and Krass 2013). Additionally, peer support serves as a central facilitator of self‐management behaviours in immigrant communities. When family members do not provide primary support for patients' self‐management activities, peer support groups with cultural competency, language assistance and culturally appropriate education in the community can be positively received (Magny‐Normilus, Mawn, and Dalton 2020; Mitchell‐Brown et al. 2017).

Cultural supports such as culturally specific education and involvement in religion were reported as facilitators in six studies. Acculturation levels and language support (e.g., clinicians or translators from the participant's community) can also facilitate effective self‐management behaviours (*n* = 3). Specifically, higher acculturation levels facilitate better behaviours related to self‐management (Chun, Chesla, and Kwan 2011), medication adherence (Alzubaidi et al. 2015) and glycaemic control (Venkatesh et al. 2013).

Regarding available resources, the themes ‘use of media’ (*n* = 3) and ‘flexible scheduling’ (*n* = 3) can promote effective self‐management behaviours. For instance, lifestyle programmes should be conducted with flexibility based on participants' jobs and family obligations, such as offering weekend and evening options (Shah et al. 2022). Only one study mentioned financial independence as a facilitator of diabetes self‐management, and participants proposed that ‘Diabetes costs are ongoing, but we work hard to afford what we need’ (Barbara and Krass 2013).

#### 3.2.2 Self‐Management Interventions

Twenty‐four (25%) studies reported on self‐management interventions for first‐generation immigrants living with prediabetes or type 2 diabetes. These studies covered various ethnic and linguistic backgrounds, such as Spanish‐speaking Americans, Korean Americans, Chinese Americans, Cantonese‐speaking Australians, Arabic‐speaking Italians, Armenian‐Americans, as well as Arabic and Turkish‐speaking immigrants in Denmark.

Eight were feasibility studies and reported acceptability and satisfaction with the interventions. Twelve studies used culturally tailored strategies to support self‐management. The intervention programmes included culturally tailored diabetes self‐management education (*n* = 7) (Brunk et al. 2017; Chesla et al. 2013; Choi and Rush 2012; Hempler et al. 2023; Kellow, Palermo, and Choi 2020; Krieg 2017; Naccashian 2014), culturally tailored behavioural interventions (*n* = 5) (Kim et al. 2009, 2015; Loya 2021; Piombo et al. 2020; Wang and Chan 2005), digital health interventions (mobile/web/social media/digital storytelling) (*n* = 5) (Hu et al. 2022, 2022; Yeh et al. 2022), integrated self‐management interventions (Kaltman et al. 2015, 2016), diabetes self‐management and social support interventions (Marylyn et al. 2007; Vang 2021), nutrition and/or physical activity education classes (Coffman et al. 2013; Ho et al. 2020), a community health worker intervention (Islam et al. 2013) and a health coaching intervention (Rechenberg et al. 2021) (Table S3).

The duration of the interventions ranged from 6 to 30 weeks, and the total number of sessions per intervention ranged from 2 to 30. The duration of sessions ranged from 45 min to 2.5 h. The findings of these programmes suggest that interventions facilitate self‐management and improve health outcomes such as clinical indicators (e.g., HbA1C, weight and BMI), diabetes knowledge, diabetes distress, family support, self‐efficacy, self‐management activities of a healthy diet and routine physical activity and quality of life (e.g., Chesla et al. 2013, 2014; Choi and Rush 2012; Kellow, Palermo, and Choi 2020; Kim et al. 2015; Naccashian 2014).

#### 3.2.3 Culturally Unique Experiences: Summary of Themes

As the participants in all 96 studies were first‐generation immigrants, their life experiences in another country were a unique cultural burden that impacted their self‐management behaviours. In Section 3.2, linguistic and cultural issues were identified, but this is insufficient to provide a more comprehensive understanding of the culturally unique experiences of this population. The culturally unique experiences of participants were explored in more detail in 12 studies and categorised into the following themes and subthemes (e.g.， Carolan‐Olah and Cassar 2018; Chun and Chesla 2004; Chun, Chesla, and Kwan 2011; Kindarara et al. 2017):

##### 3.2.3.1 Utilising Health Care

This theme included four subthemes: (1) coping with language barriers to health care, (2) accessing culturally appropriate health care services, (3) coping with economic hardships and insufficient health insurance coverage and (4) evaluating the quality of diabetes care. Many participants identified their need for greater access to interpreters and information in their first language (Carolan‐Olah and Cassar 2018; Chun, Chesla, and Kwan 2011; Jowsey, Gillespie, and Aspin 2011). Data from Pakistani‐born persons living in Norway showed that it was unrealistic for health providers to understand the cultural rationale behind their patients' actions or nonactions due to religious and cultural norms (Fagerli, Lien, and Wandel 2005).

Different participants had different experiences related to the quality of diabetes care they received. The voices of Maltese immigrants in Australia showed that they trusted their doctors and pharmacists and were willing to follow their advice about medications (Barbara and Krass 2013), while the feedback from Chinese immigrants on their diabetes care was not positive (Chun, Chesla, and Kwan 2011). As one participant who shared their experience with their diabetes diagnosis: ‘My GP did not ever suggest a blood test, even though I was overweight. I only found out by doing a self‐check … and discovered that my diabetes level was very high’. Thus, some studies recommended culturally appropriate healthcare services, which include offering interpreters or providers who know the patient's native language, translated written information (e.g., cookbooks) for diabetes, and culturally appropriate diabetes interventions that focus on bicultural competencies and skill development (Chun and Chesla 2004; Chun, Chesla, and Kwan 2011; Jowsey, Gillespie, and Aspin 2011).

##### 3.2.3.2 Maintaining Family Relations and Roles

Appropriate family support is required to sustain healthy behaviour (Barbara and Krass 2013; Chun, Chesla, and Kwan 2011; Kokanovic and Manderson 2006; Roth et al. 2022). However, separation from family members can lead to negative psychological impacts, such as social isolation, loneliness and depression. The subthemes identified: (1) coping with separation from overseas family members, (2) maintaining filial piety and respect for elders and (3) traditional family values (fulfilling family role obligations and expectations) (Barbara and Krass 2013; Chun, Chesla, and Kwan 2011; Kokanovic and Manderson 2006; Roth et al. 2022). One immigrant Australian woman said, ‘I talk to my husband. I talk to my children. They are very helpful…my husband takes time off work and takes me [to the doctor]’ (Kokanovic and Manderson 2006). The traditional family values made participants feel responsible to care for the family or guide the family in healthy eating habits (Barbara and Krass 2013). One Maltese participant expressed that: ‘I have to stay healthy to look after my husband, so if my doctor tells me to eat less sweets, then I will’ (Barbara and Krass 2013).

##### 3.2.3.3 Establishing Community Ties

Generally, participants identified an urgent need for greater community engagement as one of the most significant strategies for managing their type 2 diabetes (Chun, Chesla, and Kwan 2011; Magny‐Normilus et al. 2021). However, participants emphasised their feelings of isolation and disconnection from local communities as a daily affair. One male Haitian immigrant in the US, aged 69, said, ‘We are isolated. Even more now. Our neighbours do not really talk to us because we are different’ (Magny‐Normilus et al. 2021). The first step in establishing community ties is identifying ethnically matched neighbourhoods. For example, Chinese immigrants living in Chinatown benefitted from cultural maintenance and found it easier to meet their food preferences to manage their diabetes (Chun, Chesla, and Kwan 2011). Additionally, community centred around religion can play an important role in day‐to‐day diabetes self‐management (Magny‐Normilus et al. 2021; Roth et al. 2022). For example, in one study, older Haitian adults emphasised the strength of their church community: ‘To tell you the truth, without my family and my sisters from the church, I do not know where I would be with this disease…’.

##### 3.2.3.4 Cultural Considerations

The subthemes of cultural considerations involved (1) the conceptualisation of diabetes, illness and health; (2) culturally influenced opinions about healthy food; (3) exercise and physical activity, (4) perceptions of complementary therapies; and (5) the effects of the disease on family dynamics (Chun and Chesla 2004; Fleming, Carter, and Pettigrew 2008; Jager et al. 2019; Jamil et al. 2022). For example, Chinese Americans had a culturally unique understanding of diabetes linked to traditional Chinese medicine (TCM). From their perspective, diabetes is the ‘three excesses’ based on TCM theory, which explains their syndromes of eating a lot, drinking a lot and giving off many bodily waste materials (Fleming, Carter, and Pettigrew 2008). While participants from Gujarat living in the UK used complementary therapies such ‘herbal’, ‘ayurvedic’ or ‘homeopathic’ more frequently than they did in Gujarat due to the financial and geographical accessibility of these therapies (Fleming, Carter, and Pettigrew 2008).

#### 3.2.4 Key Outcomes, Indicators and Measures

Study outcomes were categorised into six domains according to the 2022 National Standards for Diabetes Self‐Management Education and Support (Davis et al. 2022) and the National Consensus on Outcomes and Indicators for Diabetes Education (Colagiuri and Eigenmann 2009): learning (*n* = 51), psychological (*n* = 51), behavioural (*n* = 6), sociocultural (*n* = 48), clinical (*n* = 53) and process outcomes (*n* = 22). It is important to note that these outcome indicators can overlap, which means they may fit into more than one domain. For example, some dietary behaviours, such as the participant's views on food habits, can also be viewed as sociocultural outcomes.

**FIGURE 4:**
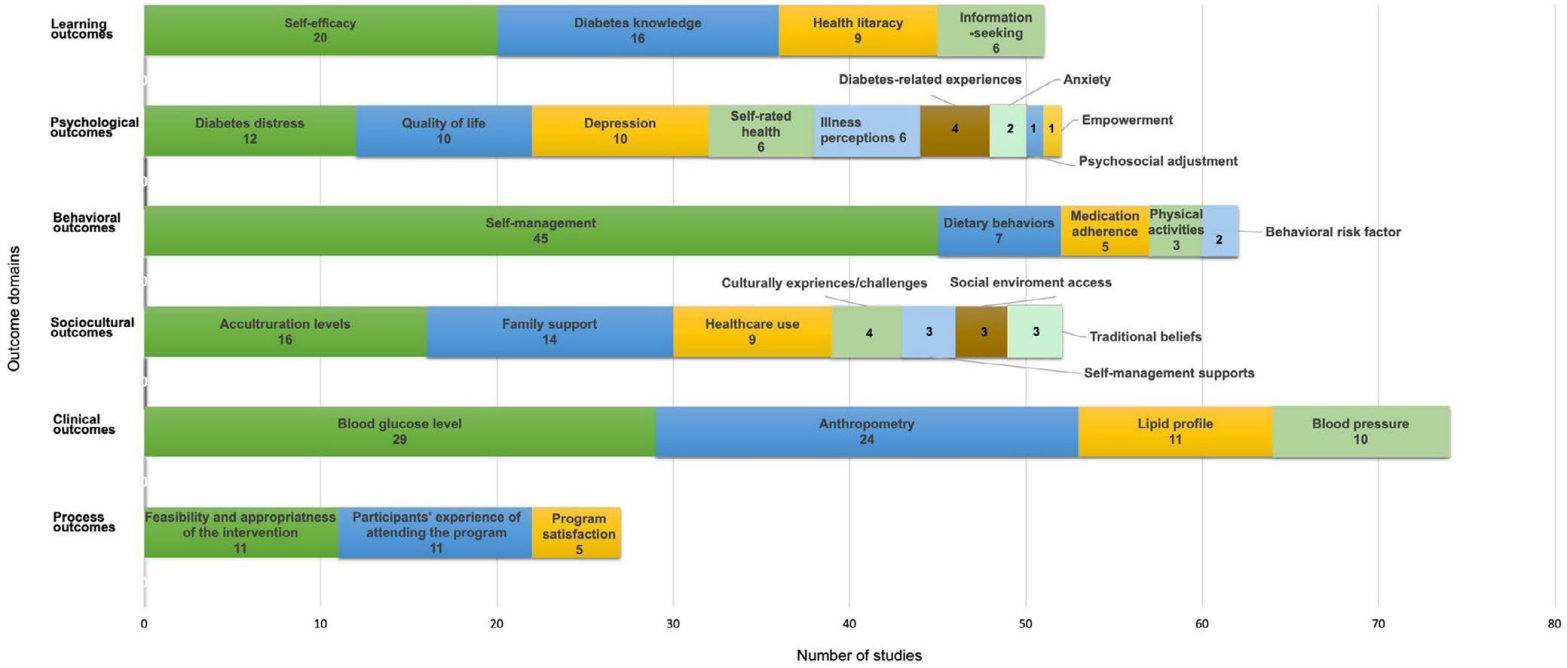
Key outcomes and indicators used in the included studies. The horizontal coordinate represents the categories of outcome domains, and the vertical coordinate represents the number of studies.

An outcome refers to a result, while an indicator refers to any unit of information that can reasonably measure progress towards achieving a result (Colagiuri and Eigenmann 2009). For this review, the outcomes and indicators were grouped and presented in Figure 4. The most common outcome indicators in descending order of frequency were self‐management (*n* = 45), blood glucose level (*n* = 29), anthropometry (*n* = 24), self‐efficacy (*n* = 20), diabetes knowledge (*n* = 16) and acculturation levels (*n* = 16). Except for clinical outcomes (e.g., glycated haemoglobin, blood pressure, body mass index), other measures were all patient‐reported outcomes.

The qualitative components were collected via interviews, open‐ended questions, logs or blogs (e.g., exercise logs, diet blogs), while the quantitative components were evaluated via validated tools or instruments. Self‐management, the most common outcome of diabetes‐related behaviours, was assessed in 45 studies, 14 of which used a summary of diabetes self‐care activities (SDSCA) (Table S5).

## 4 Discussion

Although self‐management behaviours play an increasingly vital role in delaying and managing diabetes, a comprehensive understanding of the implementation or practices of self‐management among first‐generation immigrants living with type 2 diabetes or prediabetes in different regions is lacking. Identifying associated factors, barriers or facilitators of engagement in self‐management, effective intervention strategies as well as understanding patients' cultural unique experiences are significant for developing or implementing an effective management plan for people with prediabetes or type 2 diabetes. This scoping review was conducted to provide a multiperspective understanding of available evidence and identify knowledge gaps on this topic.

Our scoping review demonstrates that not many studies paid attention to the provision of culturally sensitive support or healthcare resources to first‐generation immigrants, despite strong evidence showing that they face greater challenges than nonimmigrant groups when dealing with health and welfare systems (Abubakar et al. 2018; Montesi, Caletti, and Marchesini 2016). For countries with large immigrant populations, such as the US, Australia and Canada, diverse populations call for consideration of the provision of culturally sensitive and supportive services. It is important to note that published studies to date have focussed on the main immigrant subgroups; for example, in the US, the main three groups are Mexican, Chinese and Korean (Figure 1). Perspectives from many other smaller immigrant groups, such as those from Central America, the Pacific Islands, Southeast Asia and North Africa, are largely absent. Besides, religious minorities (e.g., Iranian Baha'i) and immigrants with complex legal statuses, including war migrants, and stateless individuals, remain understudied. This knowledge gap highlights the need to expand research on diabetes self‐management within immigrant subgroups from diverse cultural and ethnic backgrounds and countries of origin. From a global perspective, many cultural and ethnic groups, including Eastern Europeans, some African regions (e.g., Ghanaians), small Caribbean and Pacific Peoples, Latin Americans and certain Middle Eastern countries (e.g., Yemenis), are underrepresented. Information about various Asian groups, such as Burmese, Kazakhs, Japanese and Maldivians, is also lacking. Further qualitative or mixed‐methods research is essential to gain a deeper understanding of the varied health concerns, needs and migration‐specific challenges related to prediabetes and type 2 diabetes in different regions and countries.

The drivers of disparities in diabetes self‐management behaviours among immigrant groups are complex and are influenced by multiple issues. In this review, participants who were male, native‐born citizens, more educated, married or living with someone and had a longer diabetes duration, adequate social support, positive illness perceptions and low levels of acculturative stress or diabetes distress were more likely to have better self‐management behaviours. Some nonmodifiable factors (e.g., age, gender) directly and indirectly influence diabetes self‐management for all people living with diabetes. However, the extent to which nonmodifiable factors affect first‐generation immigrants differs. Older age, for example, is reported to facilitate diabetes self‐management in adults (Alexandre et al. 2021). However, our review revealed mixed effects of older age among first‐generation immigrants with a positive influence on healthy eating, medication taking and glucose monitoring, but this was not evident for foot self‐care practices (Figure 2). Migration‐specific factors are influential and include, but are not limited to, duration of immigration, country of origin, ethnicity, English proficiency and cultural background (Table S3). For instance, Mexico‐born participants demonstrated lower levels of linguistic acculturation compared to their US‐born counterparts, even after an average 31‐year US residence (Mier et al. 2012). Similarly, Arabic‐speaking immigrants showed higher diabetes distress, lower medication adherence, less effective self‐management and glycaemic control, and less favourable overall health profiles compared to Caucasian English‐speaking people (Alzubaidi et al. 2022).

Considering the unique challenges faced by diverse immigrant groups and the findings of this review, greater emphasis needs to be placed on modifiable factors (e.g., social support, healthcare access, acculturation levels) that impact diabetes self‐management. This scoping review highlights the importance of social support from family, friends and healthcare providers in improving effective self‐management behaviours across various ethnic groups, such as Cuban‐Americans, Haitian Americans, African Americans, Korean Americans, Arabic‐speaking Australians and Spanish‐speaking Americans (e.g., Alzubaidi et al. 2022). Previous evidence showed that involvement of family or spouse could be either beneficial or detrimental to type 2 diabetes self‐management (Colagiuri and Eigenmann 2009). This is consistent with the findings from this review. For example, on the one hand, autonomy support from families or spouses can help maintain effective self‐management behaviours, while on the other hand, poor family support can negatively impact health‐related behaviours, which has been observed in other cultural settings (Abel, Whitehead, and Coppell 2018; Abel et al. 2021). Indeed, how family members can best support self‐management behaviours in the targeted population (e.g., Korean Americans) still needs to be further investigated (Choi, Toyama, and Brecht 2020; Choi 2009). In addition, peer support groups—providing cultural competency, language assistance and culturally appropriate education—can play a crucial role among Haitian‐American and Hmong‐American immigrants when family members are not the primary support for self‐management activities (Magny‐Normilus, Mawn, and Dalton 2020; Mitchell‐Brown et al. 2017). Furthermore, increased levels of diabetes distress or acculturative stress has been shown to negatively impact self‐management behaviours in some immigrant subgroups, such as Arabic‐speaking Australians and Chinese‐speaking Americans (Alzubaidi et al. 2022; Huang, Zuñiga, and García 2022). These results provide new insights for researchers and healthcare providers to develop targeted interventions to manage family conflict and reduce the excess burden of diabetes distress or acculturative stress in specific immigrant groups (e.g., Cantonese‐speaking Australians, Chinese‐speaking Americans) (e.g., Alzubaidi et al. 2022; Kellow, Palermo, and Choi 2020), thus potentially improving and supporting their self‐management behaviours.

In our review, we found that many first‐generation immigrant groups often faced multiple barriers when managing their type 2 diabetes, such as access to health services, financial hardship, transportation issues, quality of provider–patient relationship and unfair and discriminatory treatment from providers (Figure 3). To illustrate this, a study on Haitian immigrants in the US described a lack of cultural understanding and sensitivity when communicating with healthcare providers. As a result, participants expressed a desire for better communication, more compassion, respect, active listening and a better understanding of their cultural differences (Magny‐Normilus, Mawn, and Dalton 2020). Developing and applying culturally appropriate strategies in patient–provider communication is of particular significance for ensuring better self‐management behaviours. This aligns with the ADA's recommendation to use a person‐centred communication style with person‐centred, culturally sensitive, strength‐based language and active listening (ADA 2024c). A further approach to support patient–provider communication gaps and opening opportunities to address structural barriers is through screening for social determinants of health (SDOH) at a system‐level. In fact, the integration of SDOH, including the policy, social and physical environments, healthcare systems, family and social networks, and poverty and discrimination faced by immigrants is consistently recommended by the ADA to be incorporated into the screening process and used to tailor treatments to people's circumstances. However, this practice has yet to become the norm (ADA 2024a; Martinez‐Cardoso, Jang, and Baig 2020). Notably, no studies in this review reported on the application of SDOH among the targeted population. A first step in addressing health inequity is awareness among researchers and healthcare providers of the SDOH framework to enable the incorporation of these into the design and delivery of diabetes self‐management programmes for first‐generation immigrants (ADA 2024d; Martinez‐Cardoso, Jang, and Baig 2020).

The review findings show that most self‐management intervention programmes yielded positive results for behaviour changes, but their effectiveness is uncertain due to insufficient or inconsistent evidence. While several studies have shown that physical activity can improve significantly after implementing interventions (e.g., Hempler et al. 2023), Kaltman's study reported that exercise was the only specific self‐management behaviour where there was no improvement (Kaltman et al. 2016). A possible reason for this inconsistent finding may be the heterogeneity of the different interventions (e.g., components, duration and delivery) and study designs. The effectiveness of the interventions needs to be interpreted with caution, as the results of studies are inconsistent, and the interventions vary in content, frequency and length of sessions, and duration of the overall intervention. For example, evidence of the efficacy of digital health interventions is limited for first‐generation immigrants, as only small‐scale feasibility studies are available, despite the strong interest in such approaches (e.g., web‐based platforms) (Hu et al. 2022; Yeh et al. 2022). Additionally, because most self‐management interventions included in this review were tested in small feasibility studies or pre‐post designs with short intervention durations, the level of evidence is insufficient. Future studies should use controlled trials with larger samples and longer follow‐up periods to validate promising results and inform evidence‐based practices or policies.

Similarly, the current evidence for culturally tailored interventions targeting diverse immigrant populations with type 2 diabetes or prediabetes is notably insufficient. Published studies are limited in scope and interventions lack systematic cultural adaptation processes as well as multi‐dimensional culturally adapted content and materials. While relatively short term, an example of an effective culturally tailored intervention is the 12‐month ‘SHIP‐DM’ programme for Korean Americans with type 2 diabetes. This intervention included structured psychobehavioural education, glucose self‐monitoring using a teletransmission system and telephone counselling provided by a trained bilingual team. This intervention offers insights for other immigrant groups by highlighting the use of bilingual resources and by combining cultural adaptation with digital health approaches. However, we postulate that overall the limited scope of interventions, to a certain extent, has led to a scarcity of culturally tailored integrated behavioural interventions, with most focusing narrowly on specific health behaviours such as physical activity, customised diet or medication adherence (e.g., Loya 2021). Moreover, many ethnic immigrant groups across diverse countries of residence, for example, sub‐Saharan African immigrants in the US (Kindarara et al. 2017), still lack culturally tailored strategies for their diabetes self‐management. These significant gaps highlight the need for more robust, culturally nuanced, person‐centred and ethnically diverse research in this field. To address these gaps, further efforts are needed to improve language and translation services in clinical encounters and diabetes education (e.g., Nam et al. 2013). More importantly, interventions for various immigrant groups should extend far beyond simply delivering diabetes self‐management education and support (DSMES) in the native language. Instead, content, educational strategies, communication styles and delivery modes need to be innovative and tailored to individual preferences, cultural beliefs, acculturation, immigrant generational status, family support level, vulnerability and access to resources, which aligns with the ADA's recommendation for person‐centred and culturally sensitive DSMES (ADA 2024d).

We have identified some commonly used indicators of self‐management behaviours from the included studies, such as self‐management scores, HbA1c, body mass index, self‐efficacy and acculturation levels. When selecting indicators and tools, it is important to consider the perceived priorities of first‐generation immigrants, along with their culturally and linguistically diverse backgrounds to ensure the trustworthiness of the research. According to Naccashian (2014), participants strongly felt that empowerment was critical for optimal self‐management; the indicator of empowerment was thus included in the outcomes of their study (Naccashian 2014). However, for some indicators, there is no preferred tool, for instance, for measuring acculturation levels. The selection of tools to assess the same indicators varied, limiting comparisons across studies (e.g., the bicultural efficacy for health management scale in two studies). Acculturation levels refers to participants' preferences in language, food, friends and identification with home or their new culture (Pranata and Wulandari 2021). However, the concept of acculturation lacks a universal definition and understanding, with inconsistencies in its operationalisation and measurement. Accordingly, calculating a score to capture a person's degree of acculturation appears obsolete (Schumann et al. 2020). Future studies on acculturation should explore migration‐specific influences on health disparities and develop a tailored approach that adequately reflects modern societies' diversity and heterogeneity (Schumann et al. 2020).

### 4.1 Limitations

This review included only studies published in English, which may have resulted in missing useful information from studies published in other languages. Current research indicates significant geographical limitations, with notable gaps observed in English‐speaking countries such as New Zealand and the UK, as well as in non‐English‐speaking destinations in Europe (e.g., France), Asia (e.g., Gulf countries) and African countries. Moreover, the results may not be applicable to other regions or populations because most studies use convenience and purposive sampling. Due to the types of studies and their mixed findings, it may be beneficial to synthesise the results in a qualitative systematic review or a quantitative meta‐analysis with implications for recommendations for practice. However, these types of studies may also be limited by the variation in the types and duration of interventions. Finally, we acknowledge that the end date of our review was February 2023 and studies published since then were not included in our data analyses. A subsequent search identified three papers that met our inclusion/exclusion critieria, including a systematic review on Arabic‐speaking immigrants (Althubyani, Gupta et al. 2024; Althubyani, Tang et al. 2024; Wan et al. 2023). The most recent literature in a systematic review on Arabic‐speaking immigrants was published to June 2022 and included only 10 studies, and the findings in all three studies align with our review; that is, the information provided by these studies does not change the findings and conclusions of our scoping review.

### 4.2 Implications for Future Research

Further research should focus on seeking more effective and feasible culturally tailored strategies for the targeted population through the lens of health equity. In addition, further research is needed to explore the benefits of combining digital health interventions with culturally tailored content as there is potential to improve access to healthcare and type 2 diabetes self‐management among minority ethnic groups. It is also necessary to choose indicators and tools that align with the patient's cultural values and preferences. For a better and deeper understanding of patient's cultural needs, a mixed‐methods design such as exploratory sequential design is a potentially good option for integrating multiple perspectives and different methods. Further, it is essential to conduct longitudinal studies to provide a comprehensive picture of the long‐term effectiveness and sustained impact of culturally tailored interventions. This review also reinforced the need for clarification of the ‘prediabetes self‐management behaviours’ among immigrant participants in further research.

### 4.3 Implications for Practice

While the evidence base is incomplete, healthcare providers are nevertheless encouraged to use a person‐centred communication style, fully considering sociodemographic factors, personal preferences, cultural sensitivity and migration‐specific challenges when providing care or interventions. Diabetes self‐management programmes should provide culturally adapted content and materials, integrate support from family, friends, peers and community, and train healthcare providers to effectively implement tailored interventions. When designing programmes, it is necessary to incorporate influencing factors (e.g., SDOH factors) and cultural considerations. Tailoring intervention protocols and delivery modes to individual needs, such as offering digital options for those facing transportation difficulties, is also important.

## 5 Conclusions

Diabetes self‐management behaviours can be influenced by a range of modifiable and nonmodifiable factors such as age, gender, education level, country of origin and other societal, linguistic, cultural and resource‐related barriers or facilitators, as well as cultural experiences that need to be considered by health professionals when providing care or self‐management interventions. Culturally and linguistically appropriate interventions that consider educational strategies, communication styles and flexible delivery modes tailored to individual and cultural preferences, are needed for adult immigrants with type 2 diabetes or prediabetes from culturally and linguistically diverse backgrounds. These interventions should be developed and implemented in collaboration with key stakeholders and the long‐term impact evaluated.

## Author Contributions

M.Z., L.W. and K.C. made substantial contributions to conception and design, or acquisition of data, or analysis and interpretation of data. M.Z. involved in drafting the manuscript or revising it critically for important intellectual content. M.Z., K.C., J.L. and L.W. given final approval of the version to be published. Each author should have participated sufficiently in the work to take public responsibility for appropriate portions of the content. M.Z., K.C., J.L. and L.W. Agreed to be accountable for all aspects of the work in ensuring that questions related to the accuracy or integrity of any part of the work are appropriately investigated and resolved.

## Conflicts of Interest

The authors declare no conflicts of interest.

### Peer Review

The peer review history for this article is available at https://www.webofscience.com/api/gateway/wos/peer‐review/10.1111/jan.16621.

## Registration

The protocol was registered in the Open Science Framework (https://osf.io/jw4h7) in November 2022.

## Supporting information


Figure S1.

Table S1.

Table S2.

Table S3.

Table S4.

Table S5.

Appendix S1.

Data S1.

## Data Availability

The data presented can be found in the Supporting Information and within the article's figures and tables.
