# Supplementary material for: Cultural Influences, Experiences and Interventions Targeting Self‐Management Behaviours for Prediabetes or Type 2 Diabetes in First‐Generation Immigrants: A Scoping Review
Source: J Adv Nurs. 2024 Nov 21;81(6):2929–45. doi: 10.1111/jan.16621 (PMC12080094; doi:10.1111/jan.16621)
Supplement: Supplementary file 1 — Figure S1. [file JAN-81-2929-s002.docx]

Identification of studies via other methods

Identification of studies via databases and registers

Identification

Records removed before screening: Duplicate records (n = 518) Records marked as ineligible by automation tools (n = NA) Records removed for other reasons (n = NA)

Records identified from: Websites (n = 308)

Organizations (n = 0)

Citation searching (n = 118)

Records identified from: Database (n = 820) Registers (n = 12)

Screening

Records excluded

(n = 102)

Records assessed for eligibility retrieval (n = 212)

Records sought for retrieval (n = 212)

Records screened (n = 314)

Records not retrieved (n = 0)

Reports not retrieved (n = 12)

Reports sought for retrieval (n = 192)

Records excluded:

Irrelevant to self-management (n = 23)

Non-1^st^ generation immigrants or including other generations (n = 90)

Participants not selected on basis of T2D/Prediabetes (n = 11)

Duplicate (n = 41)

Protocol only (n = 1)

Records excluded:

Irrelevant to self-management (n = 13)

Not 1^st^ -generation immigrants or including other generations (n = 55)

Participants not selected on basis of T2D/Prediabetes (n = 15)

Conference abstract (n = 7)

Full text unavailable (n =19)

Duplicate not previously identified (n = 10)

Protocol only (n = 11)

Reports assessed for eligibility (n = 14)

Studies included in review

(n = 82)

Reports of included studies

(n = 82)

Included

Total studies included in review (n = 96) Reports of total included studies (n = 96)

**Supplementary figure 1. The PRISMA flow chart**
