# Supplementary material for: Cultural Influences, Experiences and Interventions Targeting Self‐Management Behaviours for Prediabetes or Type 2 Diabetes in First‐Generation Immigrants: A Scoping Review
Source: J Adv Nurs. 2024 Nov 21;81(6):2929–45. doi: 10.1111/jan.16621 (PMC12080094; doi:10.1111/jan.16621)
Supplement: Supplementary file 2 — Table S1. [file JAN-81-2929-s003.docx]

## **Supplementary table 1 Search strategy**

**1. CINAHL (EBSCOhost CINAHL Plus with Full Text)**

Search conducted in Feb 2023.

| **Search** | **Query** | **Records retrieved** |
| --- | --- | --- |
| S1 | (MM "Diabetes Mellitus, Type 2") | 56,608 |
| S2 | AB ("Type 2 Diabetes*" OR "Diabetes, Type 2" OR "Diabetes Mellitus, Stable" OR "Stable Diabetes Mellitus" OR " Type II Diabetes*" OR "Diabetes Mellitus, Type II" OR "NIDDM" OR "Noninsulin-Dependent Diabetes Mellitus" OR "Noninsulin Dependent Diabetes Mellitus" OR "Diabetes Mellitus, Noninsulin Dependent" OR "Diabetes Mellitus, Non Insulin Dependent" OR "Diabetes Mellitus, Non-Insulin-Dependent" OR "Non-Insulin-Dependent Diabetes Mellitus") | 48,649 |
| S3 | (MM "Prediabetic State") | 2,877 |
| S4 | AB ("Prediabetic States" OR "State*, Prediabetic" OR Prediabetes OR Pre-diabet*) | 3,638 |
| S5 | S1 OR S2 OR S3 OR S4 | 77,828 |
| S6 | (MM "Self-Management") OR (MM "Self Care+") OR (MM "Self Assessment") | 35,782 |
| S7 | AB ("Self Management" OR "Management, Self" OR "Self-care" OR "Care, Self" OR "Self-administration" OR "Self Administration*"OR "Administration*, Self" OR "Self Medication" OR "Medication*, Self " OR Self-assessment OR "Self Assessment*" OR "Assessment*, Self" OR Self-efficacy OR "Efficacy, Self" OR " Blood Glucose Self-Monitoring") | 62,470 |
| S8 | S6 OR S7 | 85,727 |
| S9 | (MM "Emigration and Immigration") OR (MM "Immigrants+") OR (MM "Undocumented Immigrants") | 16,954 |
| S10 | AB ("Immigrants and Emigrants" OR Immigrant* OR Foreigner* OR Alien* OR Emigrant* OR "Immigration and Emigration" OR Migration* OR Emigration* OR Immigration* OR In-Migration* OR "In Migration") | 42,964 |
| S11 | S9 OR S10 | 50,482 |
| S12 | S5 AND S8 AND S11 | 82 |
| **Limiters** - English Language; Human  **Expanders** - Apply equivalent subjects  **Search modes** - Boolean/Phrase | | **71** |

**2. Cochrane Library**

Search conducted in Feb 2023.

| **Search** | **Query** | **Records retrieved** |
| --- | --- | --- |
| #1 | (MH "Diabetes Mellitus, Type 2") | 239 |
| #2 | (("Type 2 Diabetes*" OR "Diabetes, Type 2" OR "Diabetes Mellitus, Stable" OR "Stable Diabetes Mellitus" OR " Type II Diabetes*" OR "Diabetes Mellitus, Type II" OR "NIDDM" OR "Noninsulin-Dependent Diabetes Mellitus" OR "Noninsulin Dependent Diabetes Mellitus" OR "Diabetes Mellitus, Noninsulin Dependent" OR "Diabetes Mellitus, Non Insulin Dependent" OR "Diabetes Mellitus, Non-Insulin-Dependent" OR "Non-Insulin-Dependent Diabetes Mellitus")):ti,ab,kw | 46,503 |
| #3 | (MH "Prediabetic State") | 10 |
| #4 | (("Prediabetic States" OR "State*, Prediabetic" OR Prediabetes OR Pre-diabet*)):ti,ab,kw | 3,534 |
| #5 | #1 OR #2 OR #3 OR #4 | 48639 |
| S6 | (MM "Self-Management") OR (MM "Self Care+") OR (MM "Self Assessment") | 1094 |
| S7 | (("Self Management" OR "Management, Self" OR "Self-care" OR "Care, Self" OR "Self-administration" OR "Self Administration*" OR "Administration*, Self" OR "Self Medications" OR "Medication*, Self " OR Self-assessment OR "Self Assessment*" OR "Assessment*, Self" OR Self-efficacy OR "Efficacy, Self")):ti,ab,kw | 40,460 |
| S8 | #6 OR #7 | 40,687 |
| S9 | (MM "Emigration and Immigration") OR (MM "Immigrants+") OR (MM "Undocumented Immigrants") | 35 |
| S10 | (("Immigrants and Emigrants" OR Immigrant* OR Foreigner* OR Alien* OR Emigrant* OR "Immigration and Emigration" OR Migration* OR Emigration* OR Immigration* OR In-Migration* OR "In Migration")):ti,ab,kw | 5,169 |
| S11 | #9 OR #10 | 5,180 |
| S12 | #5 AND #8 AND #11 | **24** |

**3.EMBASE (Elsevier)**

Search conducted in Feb 2023.

| **Search** | **Query** | **Records retrieved** |
| --- | --- | --- |
| #1 | 'non insulin dependent diabetes mellitus'/exp OR ('adult onset diabetes' OR 'adult onset diabetes mellitus' OR 'diabetes mellitus type 2' OR 'diabetes mellitus type ii' OR 'diabetes mellitus, non-insulin-dependent' OR 'diabetes mellitus, type 2' OR 'diabetes mellitus, type II' OR 'diabetes mellitus, maturity onset' OR 'diabetes mellitus, non insulin dependent' OR 'diabetes type 2' OR 'diabetes type II' OR 'diabetes, adult onset' OR 'dm 2' OR 'insulin independent diabetes' OR 'insulin independent diabetes mellitus' OR 'ketosis resistant diabetes mellitus' OR 'maturity onset diabetes' OR 'maturity onset diabetes mellitus' OR 'maturity onset diabetes of the young' OR 'niddm' OR 'NIDDM (non insulin dependent diabetes mellitus)' OR 'non insulin dependent diabetes' OR 'noninsulin dependent diabetes' OR 'noninsulin dependent diabetes mellitus' OR 'T2DM' OR 'type 2 diabetes' OR 'type 2 diabetes mellitus' OR 'type II diabetes'):ab,ti,kw | 379,053 |
| #2 | 'impaired glucose tolerance'/exp OR ('chemical diabetes' OR 'chemical diabetes mellitus' OR 'diabetes mellitus, potential' OR 'diabetes, latent' OR 'genetic prediabetes' OR 'glucose tolerance impairment' OR 'glucose tolerance, potentially impaired' OR 'impaired glucose tolerance, potential' OR 'latent diabetes' OR 'latent diabetes mellitus' OR 'potential diabetes' OR 'potential diabetes mellitus' OR 'potential glucose tolerance impairment' OR 'pre diabetes mellitus' OR 'prediabetes' OR 'prediabetes mellitus' OR 'prediabetic stage' OR 'prediabetic state'):ab,ti,kw | 47,884 |
| #3 | 'self care'/exp OR 'self help'/exp OR 'self medication'/exp OR ('self management' OR 'self treatment' OR 'self-management' OR 'self-nurturance' OR 'selfcare' OR 'selfmanagement' OR 'selftreatment' OR 'group, self help' OR 'self help group' OR 'self-help groups' OR 'selfhelp'):ab,ti,kw | 128,236 |
| #4 | 'immigrant'/exp OR ('emigrants and immigrants' OR 'migrants'):ab,ti,kw | 45,361 |
| #5 | #1 OR #2 | 406,815 |
| #6 | #3 AND #4 AND #5 | 121 |
| #7 | #3 AND #4 AND #5 AND [english]/lim AND [humans]/lim | **119** |

**4. MEDLINE (EBSCO)**

Search conducted in Feb 2023.

| **Search** | **Query** | **Records retrieved** |
| --- | --- | --- |
| S1 | (MH "Diabetes Mellitus, Type 2") OR ("Type 2 Diabetes*" OR "Diabetes, Type 2" OR "Diabetes Mellitus, Stable" OR "Stable Diabetes Mellitus" OR "Diabetes Mellitus, Type II" OR "NIDDM" OR "Noninsulin-Dependent Diabetes Mellitus" OR "Noninsulin Dependent Diabetes Mellitus" OR "Diabetes Mellitus, Noninsulin Dependent" OR "Diabetes Mellitus, Non Insulin Dependent" OR "Diabetes Mellitus, Non-Insulin-Dependent") | 221,129 |
| S2 | (MH "Prediabetic State") OR ("Prediabetic States" OR "State*, Prediabetic" OR Prediabetes OR Pre-diabet*) | 14,345 |
| S3 | ((MH "Self-Management") OR (MH "Self Efficacy") OR (MH "Self-Assessment") OR (MH "Self Care") OR (MH "Self Medication") OR (MH "Self Administration")) OR ("Self Management" OR "Management, Self" OR "Self-care" OR "Care, Self" OR "Self-administration" OR "Self Administration*" OR "Administration*, Self" OR "Self Medications" OR "Medication*, Self " OR Self-assessment OR "Self Assessment*" OR "Assessment*, Self" OR Self-efficacy OR "Efficacy, Self") | 156,217 |
| S4 | ((MH "Emigration and Immigration") OR (MH "Emigrants and Immigrants") OR (MH "Undocumented Immigrants")) OR ("Immigrants and Emigrants" OR Immigrant* OR Foreigner* OR Alien* OR Emigrant* OR "Immigration and Emigration" OR Migration* OR Emigration* OR Immigration* OR In-Migration* OR "In Migration") | 406,566 |
| S5 | S1 OR S2 | 229,320 |
| S6 | S3 AND S4 AND S5 | 144 |
| Limiters - English Language; Human  Expanders - Apply equivalent subjects  Search modes - Boolean/Phrase | | **131** |

**5.** [**ProQuest One Academic**](https://ecu.au.libguides.com/proquest-one)

Search conducted in Feb 2023.

| **Search** | **Query** | **Records retrieved** |
| --- | --- | --- |
| S1 | subject((("Diabetes Mellitus, Type 2") OR ("Prediabetic State"))) | 52,860 |
| S2 | all(("Type 2 Diabetes*" OR "Diabetes, Type 2" OR "Diabetes Mellitus, Stable" OR "Stable Diabetes Mellitus" OR " Type II Diabetes*" OR "Diabetes Mellitus, Type II" OR "NIDDM" OR "Noninsulin-Dependent Diabetes Mellitus" OR "Noninsulin Dependent Diabetes Mellitus" OR "Diabetes Mellitus, Noninsulin Dependent" OR "Diabetes Mellitus, Non Insulin Dependent" OR "Diabetes Mellitus, Non-Insulin-Dependent" OR "Non-Insulin-Dependent Diabetes Mellitus" OR "Prediabetic States" OR "State*, Prediabetic" OR Prediabetes OR Pre-diabet*)) | 240,462 |
| S3 | S1 OR S2 | 255,041 |
| S4 | subject((("Self-Management") OR ("Self Efficacy") OR ("Self-Assessment") OR ("Self Care") OR ("Self Medication") OR ("Self Administration"))) | 88,312 |
| S5 | all(("Self Management" OR "Management, Self" OR "Self-care" OR "Care, Self" OR "Self-administration" OR "Self Administration*" OR "Administration*, Self" OR "Self Medications" OR "Medication*, Self " OR Self-assessment OR "Self Assessment*" OR "Assessment*, Self" OR Self-efficacy OR "Efficacy, Self")) | 243,840 |
| S6 | S4 OR S5 | 249,564 |
| S7 | subject((("Emigration and Immigration") OR ("Emigrants and Immigrants") OR ("Undocumented Immigrants"))) | 8,120 |
| S8 | all(("Immigrants and Emigrants" OR Immigrant* OR Foreigner* OR Alien* OR Emigrant* OR "Immigration and Emigration" OR Migration* OR Emigration* OR Immigration* OR In-Migration* OR "In Migration")) | 4,264,194 |
| S9 | S7 OR S8 | 4,257,469 |
| S10 | S3 AND S6 AND S9 | **160** |

**6. PsycINFO (EBSCOhost)**

Search conducted in Feb 2023.

| **Search** | **Query** | **Records retrieved** |
| --- | --- | --- |
| S1 | MA "Diabetes Mellitus, Type 2" | 4,794 |
| S2 | AB ("Type 2 Diabetes*" OR "Diabetes, Type 2" OR "Diabetes Mellitus, Stable" OR "Stable Diabetes Mellitus" OR " Type II Diabetes*" OR "Diabetes Mellitus, Type II" OR "NIDDM" OR "Noninsulin-Dependent Diabetes Mellitus" OR "Noninsulin Dependent Diabetes Mellitus" OR "Diabetes Mellitus, Noninsulin Dependent" OR "Diabetes Mellitus, Non Insulin Dependent" OR "Diabetes Mellitus, Non-Insulin-Dependent" OR "Non-Insulin-Dependent Diabetes Mellitus") | 8,638 |
| S3 | MA "Prediabetic State" | 192 |
| S4 | AB ("Prediabetic States" OR "State*, Prediabetic" OR Prediabetes OR Pre-diabet*) | 640 |
| S5 | S1 OR S2 OR S3 OR S4 | 10,442 |
| S6 | MA Self-Management OR MA Self Care+ OR MA "Self Assessment" | 6,406 |
| S7 | AB ("Self Management" OR "Management, Self" OR "Self-care" OR "Care, Self" OR "Self-administration" OR "Self Administration*"OR "Administration*, Self" OR "Self Medication" OR "Medication*, Self " OR Self-assessment OR "Self Assessment*" OR "Assessment*, Self" OR Self-efficacy OR "Efficacy, Self" OR " Blood Glucose Self-Monitoring") | 81,305 |
| S8 | S6 OR S7 | 86,364 |
| S9 | MA ( ("Emigration and Immigration") ) OR MA "Immigrants+" OR MA ("Undocumented Immigrants") | 2,936 |
| S10 | AB ("Immigrants and Emigrants" OR Immigrant* OR Foreigner* OR Alien* OR Emigrant* OR "Immigration and Emigration" OR Migration* OR Emigration* OR Immigration* OR In-Migration* OR "In Migration") | 63,197 |
| S11 | S9 OR S10 | 64,094 |
| S12 | S5 AND S8 AND S11 | **54** |

**7. SCOPUS**

Search conducted in Feb 2023.

| **Search** | **Query** | **Records retrieved** |
| --- | --- | --- |
| 1 | TITLE-ABS-KEY ( ( ( "diabetes mellitus, type 2" ) OR ( "type 2 diabetes*" ) OR ( "stable diabetes mellitus" ) OR ( "diabetes mellitus, type ii" ) OR ( "niddm" ) OR ( "noninsulin dependent diabetes mellitus" ) OR ( "diabetes mellitus, non-insulin-dependent" ) ) ) | 247,664 |
| 2 | TITLE-ABS-KEY ( ( ( "prediabetic state*" ) OR ( prediabetes ) OR ( pre-diabet* ) ) ) | 15,723 |
| 3 | TITLE-ABS-KEY ( ( ( "Self-Management" )  OR  ( "Self Efficacy" )  OR  ( "Self-Assessment*" )  OR  ( "Self Care" )  OR  ( "Self Medication*" )  OR  ( "Self Administration*" )  OR  ( "Self Management" )  OR  ( "Self-care" )  OR  ( "Self-administration" )  OR  ( self-assessment )  OR  ( self-efficacy ) ) ) | 242,023 |
| 4 | TITLE-ABS-KEY ( ( ( "Emigration and Immigration" )  OR  ( "Emigrants and Immigrants" )  OR  ( "Undocumented Immigrants" )  OR  ( "Immigrants and Emigrants" )  OR  ( immigrant* )  OR  ( foreigner* )  OR  ( alien* )  OR  ( emigrant* )  OR  ( "Immigration and Emigration" )  OR  ( migration* )  OR  ( emigration* )  OR  ( immigration* )  OR  ( in-migration* )  OR  ( "In Migration" ) ) ) | 988,059 |
| 5 | S1 OR S2 | 256,609 |
| 6 | S3 AND S4 AND S5 | 144 |
| LIMIT-TO ( LANGUAGE , "english" | | **141** |

**8. Web of Science**

Search conducted in Feb 2023.

| **Search** | **Query** | **Records retrieved** |
| --- | --- | --- |
| 1 | TS=(("Diabetes Mellitus, Type 2") OR ("Type 2 Diabetes*" OR "Diabetes, Type 2" OR "Diabetes Mellitus, Stable" OR "Stable Diabetes Mellitus" OR "Diabetes Mellitus, Type II" OR "NIDDM" OR "Noninsulin-Dependent Diabetes Mellitus" OR "Noninsulin Dependent Diabetes Mellitus" OR "Diabetes Mellitus, Noninsulin Dependent" OR "Diabetes Mellitus, Non Insulin Dependent" OR "Diabetes Mellitus, Non-Insulin-Dependent")) | 216,969 |
| 2 | TS=(("Prediabetic State") OR ("Prediabetic States") OR ("State*, Prediabetic") OR Prediabetes OR Pre-diabet*) | 12,644 |
| 3 | TS=((("Self-Management") OR ("Self Efficacy") OR ("Self-Assessment") OR ("Self Care") OR ("Self Medication") OR ("Self Administration") OR ("Self Management" OR "Management, Self" OR "Self-care" OR "Care, Self" OR "Self-administration" OR "Self Administration*" OR "Administration*, Self" OR "Self Medications" OR "Medication*, Self " OR Self-assessment OR "Self Assessment*" OR "Assessment*, Self" OR Self-efficacy OR "Efficacy, Self"))) | 188,221 |
| 4 | TS=((("Emigration and Immigration") OR ("Emigrants and Immigrants") OR ("Undocumented Immigrants") OR ("Immigrants and Emigrants") OR Immigrant* OR Foreigner* OR Alien* OR Emigrant* OR ("Immigration and Emigration") OR Migration* OR Emigration* OR Immigration* OR (In-Migration*) OR ("In Migration"))) | 810,734 |
| 5 | S1 OR S2 | 224,500 |
| 6 | S3 AND S4 AND S5 | 120 |
| Limiters: English Language | | **120** |

**9. WHO - International Clinical Trials Registry Platform (ICTRP)**

Search conducted in Feb 2023.

| **Search** | **Query** | **Records retrieved** |
| --- | --- | --- |
| 1 | ((("Diabetes Mellitus, Type 2") OR ("Type 2 Diabetes*") OR ("Prediabetic State") OR Prediabetes) AND (Immigrant* OR Foreigner* OR Alien* OR Emigrant* OR Immigration* OR In-Migration* OR "In Migration"))  AND ("Self Management" OR "Management, Self" OR "Self-care" OR "Care, Self" OR "Self-administration" OR "Self Administration*"OR "Administration*, Self" OR "Self Medication" OR "Medication*, Self " OR Self-assessment OR "Self Assessment*" OR "Assessment*, Self" OR Self-efficacy OR "Efficacy, Self") | 12 |

**10. Google Scholar**

Search conducted in Feb 2023.

| **Search** | **Query** | **Records retrieved** |
| --- | --- | --- |
| 1 | ((("Diabetes Mellitus, Type 2") OR ("Prediabetic State") AND (Immigrant*))) AND ((Self-management) OR (Self-care) OR (self-efficacy) OR (self-assessment*) OR (self-medication*) OR (self-administration*)) | 217 |

**11. Open Grey**

Search conducted in Feb 2023.

| **Search** | **Query** | **Records retrieved** |
| --- | --- | --- |
| 1 | ((("Diabetes Mellitus, Type 2") OR ("Type 2 Diabetes*") OR ("Prediabetic State") OR Prediabetes)) AND Immigrants | 1 |
| 2 | ((("Diabetes Mellitus, Type 2") OR ("Type 2 Diabetes*") OR ("Prediabetic State") OR Prediabetes)) AND management | 6 |
| In total |  | 7 |

**12. World-Cat**

Search conducted in Feb 2023.

| **Search** | **Query** | **Records retrieved** |
| --- | --- | --- |
| 1 | ((("Diabetes Mellitus, Type 2") OR ("Type 2 Diabetes*") OR ("Prediabetic State") OR Prediabetes) AND (Immigrant* OR Foreigner* OR Alien* OR Emigrant* OR Immigration* OR In-Migration* OR "In Migration"))  AND ("Self Management" OR "Management, Self" OR "Self-care" OR "Care, Self" OR "Self-administration" OR "Self Administration*"OR "Administration*, Self" OR "Self Medication" OR "Medication*, Self " OR Self-assessment OR "Self Assessment*" OR "Assessment*, Self" OR Self-efficacy OR "Efficacy, Self")  **Language: English** | 85 |
