# Supplementary material for: Cultural Influences, Experiences and Interventions Targeting Self‐Management Behaviours for Prediabetes or Type 2 Diabetes in First‐Generation Immigrants: A Scoping Review
Source: J Adv Nurs. 2024 Nov 21;81(6):2929–45. doi: 10.1111/jan.16621 (PMC12080094; doi:10.1111/jan.16621)
Supplement: Supplementary file 3 — Table S2. [file JAN-81-2929-s008.docx]

## **Supplementary table 2: Studies excluded on full text**

| **Items** | **Title** | **Reason for exclusion** |
| --- | --- | --- |
| *1* | *Amirehsani KA, Hu J, Wallace DC, McCoy TP. Herbal/Plant remedies and supplements used by Hispanics/Latinxs for diabetes: source of functional foods?* | Duplicate study |
| 2 | *Amirehsani KA, Wallace DC. Tes, Licuados, and Capsulas: herbal self-care remedies of Latino/Hispanic immigrants for type 2 diabetes.* | Ineligible population |
| 3 | *Andersen E, Burton NW, Anderssen SA. Physical activity levels six months after a randomised controlled physical activity intervention for Pakistani immigrant men living in Norway* | Duplicate study |
| 4 | *Balcha S. A systematic review of the relationship between type 2 diabetes and self-care practices in Ethiopian immigrants* | Ineligible phenomena of interest |
| 5 | *Ballotari P, Ferrari F, Ballini L, Chiarenza A, Manicardi V, Giorgi Rossi P. Lifestyle-tailored interventions for South Asians with type 2 diabetes living in high-income countries: a systematic review* | Conference abstract |
| 6 | *Barko R, Corbett CF, Allen CB, Shultz JA. Perceptions of diabetes symptoms and self-management strategies: A cross-cultural comparison* | Ineligible population |
| 7 | *Bedwell R. Diabetes Illness Narratives Among Mexican Immigrants in the U.S.- Mexico Border Region* | Ineligible condition |
| 8 | *Berry D, Colindres M, Sanchez-Lugo L, Sanchez M, Neal M, Smith-Miller C. Adapting, feasibility testing, and pilot testing a weight management intervention for recently immigrated Spanish-speaking women and their 2- to 4-year-old children* | Ineligible condition |
| 9 | *Bhattacharya G. Psychosocial impacts of type 2 diabetes self-management in a rural African-American population.* | Ineligible condition |
| 10 | *Brown K, Avis M, Hubbard M. Health beliefs of African-Caribbean people with type 2 diabetes: a qualitative study.* | Ineligible population |
| 11 | *Castillo ADS. Stress reduction with Tai Chi for elderly Hispanics with diabetes* | Ineligible population |
| 12 | *Catherine Chesla. Community based participatory research with immigrant Chinese with type 2 diabetes: adapting and testing coping skills training* | Ineligible population |
| 13 | *Cherlie Magny-Normilus P. Self-management and glycemic control in adult Haitian immigrants with type 2 diabetes: A pilot randomized study* | Protocol only |
| 14 | *Chesla CA, Chun KM, Kwan CM. Overcoming family and cultural challenges for Chinese American patients with diabetes: Provider guidelines. Diabetes* | Protocol only |
| 15 | *Chesla CA, Chun KM, Kwan CML, Mullan JT, Kwong Y, Hsu L, et al. Testing the efficacy of culturally adapted coping skills training for Chinese American immigrants with type 2 diabetes using community-based participatory research.* | Conference abstract |
| 16 | *Chidi O. Use of diabetes self-management education to improve type 2 diabetes mellitus among adult Hispanics* | Duplicate study |
| 17 | *Choi SE. Effect of culturally-tailored community-based diabetes self-management program for Korean immigrants: A pilot study* | Full text unavailable |
| 18 | *Chun KM, Kwan CML, Strycker LA, Chesla CA. Acculturation and bicultural efficacy effects on Chinese American immigrants’ diabetes and health management* | Duplicate study |
| 19 | *Clingerman E. Type 2 diabetes among migrant and seasonal farmworkers* | Ineligible phenomena of interest |
| 20 | *Cuevas HE, Brown SA. Cuban Americans (CAs) and type 2 diabetes: an integrative review of the literature* | Ineligible population |
| 21 | *De Man J, Kasujja FX, Delobelle P, Annerstedt KS, Alvesson HM, Absetz P, et al. Motivational determinants of physical activity in disadvantaged populations with (pre)diabetes: a cross-cultural comparison.* | Ineligible population |
| 22 | *Deng FY, Zhang AR, Chan C. Nutrition interventions for type 2 diabetes in Chinese populations: A scoping review* | Ineligible population |
| 23 | *Didulo DL. Correlates of diabetes self-management among Filipinos* | Ineligible population |
| 24 | *Doyle G, Gibney S, Quan J, Martensen U, Schillinger D. Health literacy, health care utilization, and direct cost of care among linguistically diverse patients with type 2 diabetes mellitus* | Full text unavailable |
| 25 | *Felix H, Rowland B, Long CR, Marie-Rachelle N, Piel M, Goulden PA, et al. Diabetes Self-Care Behaviors Among Marshallese Adults Living in the United States* | Ineligible phenomena of interest |
| 26 | *Francis SL, Keane M. Extension-delivered diabetes program for rural-residing latinos improves diabetes self-management, self-efficacy and knowledge.* | Ineligible population |
| 27 | *Futterman Collier AD, Cole DR, Tuulua L, Sellman JD. Cultural identity, psychosocial factors, and glycemic control in New Zealand Samoan people with non-insulin dependent diabetes mellitus. Psychosomatic Medicine* | Full text unavailable |
| 28 | *Gerd Holmboe-Ottesen. Evaluation of a Lifestyle Intervention to Prevent Type 2 Diabetes/Metabolic Syndrome Among Pakistani Immigrants - Focusing on Changes in Diet and Physical Activity* | Full text unavailable |

| **Items** | **Title** | **Reason for exclusion** | |
| --- | --- | --- | --- |
| 29 | *Gold R, Yu K, Liang LJ, Adler F, Balingit P, Luc P, et al. Synchronous provider visit and self-management education improves glycemic control in hispanic patients with long-standing type 2 diabetes.* | | Protocol only |
| 30 | *Grant RC, Retnakaran RR. Healthcare, self-care, and health status of immigrants and non-immigrants with type 2 diabetes in the Canadian Community Health Surveys* | | Ineligible population |
| 31 | *Hadziabdic E, Pettersson S, Marklund H, Hjelm K. Development of a group-based diabetes education model for migrants with type 2 diabetes, living in Sweden. Primary health care research & development* | | Ineligible population |
| 32 | *Hamzah A, Sulieman H, Mc Namara K, Samorinha C, Browning C. The relationship between diabetes distress, medication taking, glycaemic control and self-management. International Journal of Clinical Pharmacy* | | Full text unavailable |
| 33 | *Hu L, Cheng S, Islam N, Wylie-Rosett J, Wu B, Feldman N, et al. Feasibility and Acceptability of a Family-Based mHealth Intervention in Low-Income Chinese Families with Type 2 Diabetes* | | Duplicate study |
| 34 | *Hu L, Islam N, Jiang N, Tamura K, Jagannathan R, Kharmats AY, et al. Preliminary Outcomes of a Social Media-Based Diabetes Intervention among Low-Income Chinese Immigrants* | | Full text unavailable |
| 35 | *Hu L, Islam N, Kharmats AY, Tamura K, Yang S, Xu X, et al. A social media-based intervention improves glycemic control in a low-income older immigrant population. Diabetes [* | | Full text unavailable |
| 36 | *Hu L, Islam N, Zhang Y, Shi Y, Li H, Chan W, et al. Leveraging Social Media to Increase Access to an Evidence-Based Diabetes Intervention Among Low-Income Chinese Immigrants: Protocol for a Pilot Randomized Controlled Trial. JMIR Research Protocols* | | Duplicate study |
| 37 | *Jeong M, Reifsnider E. Associations of Diabetes-Related Distress and Depressive Symptoms With Glycemic Control in Korean Americans With Type 2 Diabetes.* | | Protocol only |
| 38 | *Jessri M, Sadighi P, Toofani N, Woods L, L’Abbe M. Unheard voices of Iranian immigrants with type 2 diabetes: a Canadian focused ethnographic study.* | | Ineligible phenomena of interest |
| 39 | *Joo JY. Effectiveness of Culturally Tailored Diabetes Interventions for Asian Immigrants to the United States A Systematic Review. Diabetes Educator* | | Full text unavailable |
| 40 | *Jordan DN. Comorbidities, perceptions, self-care behaviors, and foot self-care practices of Filipino American adults with type 2 diabetes mellitus* | | Ineligible population |
| 41 | *Jowsey T, Gillespie J, Aspin C. Effective communication is crucial to self-management: The experiences of immigrants to Australia living with diabetes. Chronic Illness* | | Full text unavailable |
| 42 | *Katz AM, Koski-Karell V, Jean-Baptiste ML, Picard KC. Participating in diabetes (care): The art of accompaniment, enactment of illness, and cross-cultural care. Journal of General Internal Medicine* | | Ineligible population |
| 43 | *Khan S, Shah N, Parikh N, Iyer D, Palaniappan L. Type 2 diabetes mellitus in South Asian Americans. In: Biopsychosocial approaches to understanding health in South Asian Americans* | | Full text unavailable |
| 44 | *Khunti K, Camosso-Stefinovic J, Carey M, Davies MJ, Stone MA. Educational interventions for migrant South Asians with Type 2 diabetes: a systematic review. Diabetic medicine : a journal of the British Diabetic Association* | | Ineligible phenomena of interest |
| 45 | *Kim MT, Kim KB, Ko J, Jang Y, Levine D, Lee HB. Role of depression in diabetes management in an ethnic minority population: a case of Korean Americans with type 2 diabetes* | | Ineligible population |
| 46 | *Kindarara DM. Sub-Saharan African immigrants’ health-illness transition experiences with Type 2 diabetes self-management in the United States* | | Ineligible condition |
| 47 | *Kollannoor-Samuel G, Vega-López S, Chhabra J, Segura-Pérez S, Damio G, Pérez-Escamilla R. Food insecurity and low self-efficacy are associated with health care access barriers among Puerto-Ricans with type 2 diabetes* | | Duplicate study |
| 48 | *Kuske S, Schiereck T, Grobosch S, Paduch A, Droste S, Halbach S, et al. Correction to: Diabetes-related information-seeking behaviour: a systematic review.* | | Ineligible phenomena of interest |
| 49 | *Lanting LC, Joung IMA, Vogel I, Bootsma AH, Lamberts SWJ, Mackenbach JP. Ethnic differences in outcomes of diabetes care and the role of self-management behavior. Patient Education and Counseling* | | Ineligible phenomena of interest |
| 50 | *Latham CL, Calvillo E. Predictors of diabetes outcomes in Mexico: testing the Hispanic health protection model.* | | Ineligible condition |
| 51 | *Lee H, Woo BKP. Perceptions of and Attitudes Toward Diabetes Among Chinese Americans.* | | Ineligible population |
| 52 | *Li-Geng T, Kilham J, McLeod KM. Cultural Influences on Dietary Self-Management of Type 2 Diabetes in East Asian Americans: A Mixed-Methods Systematic Review.* | | Duplicate study |
| 53 | *Lim S, Wyatt LC, Mammen S, Zanowiak JM, Mohaimin S, Troxel AB, et al. Implementation of a multi-level community-clinical linkage intervention to improve glycemic control among south Asian patients with uncontrolled diabetes: study protocol of the DREAM initiative.* | | Ineligible population |
| 54 | *Louise Bennet. A Middle Eastern Immigrant Population At-risk for Diabetes; Contributing Risk Factors and the Efficiency and Cost-effectiveness of a Culturally Adopted Lifestyle Intervention Program - the MEDIM Study.* | | Protocol only |
| 55 | *Louise Bennet. Community-Based Diabetes Care for Korean American Immigrants* | | Ineligible population |
| 56 | *Macaden L. Risk perception among older south asians with type 2 diabetes in the united kingdom* | | Protocol only |

| **Items** | **Title** | **Reason for exclusion** | |
| --- | --- | --- | --- |
| 57 | *Magny-Normilus C, Whittemore R. Haitian Immigrants and Type 2 Diabetes: An Integrative Review. Journal of Immigrant and Minority Health* | | Ineligible phenomena of interest |
| 58 | *Magny-Normilus C. Self-Management of Type 2 Diabetes Mellitus in the Context of Adult Haitian Immigrant Culture: A Concept Analysis* | | Ineligible population |
| 59 | *Magny-Normilus C. Self-management of type 2 diabetes mellitus: The lived experience of adult Haitian immigrants. Self-Management of Type 2 Diabetes Mellitus: The Lived Experience of Adult Haitian Immigrants* | | Full text unavailable |
| 60 | *Majeed R. Understanding the self-management of type 2 diabetes in black and minority ethnic groups using a health literacy framework* | | Ineligible population |
| 61 | *Majeed-Ariss R, Jackson C, Knapp P, Cheater FM. British-Pakistani women’s perspectives of diabetes self-management: the role of identity* | | Ineligible population |
| 62 | *Maneze D, Everett B, Astorga C, Yogendran D, Salamonson Y. The Influence of Health Literacy and Depression on Diabetes Self-Management: A Cross-Sectional Study* | | Ineligible population |
| 63 | *Mansyur CL, Rustveld LO, Nash SG, Jibaja-Weiss ML. Social factors and barriers to self-care adherence in Hispanic men and women with diabetes* | | Ineligible population |
| 64 | *McEwen MM, Pasvogel A, Gallegos G, Barrera L. Type 2 diabetes self-management social support intervention at the U.S.-Mexico border* | | Ineligible population |
| 65 | *McGowan PT. The punjabi diabetes self-management program* | | Ineligible population |
| 66 | *Megha SM. Better Together: A Patient-centered Approach to Improve Diabetes Among Immigrant Communities* | | Conference abstract |
| 67 | *Mikell M. Exploring Factors Influencing Health Promoting Behaviors Among Latino Immigrants. Exploring Factors Influencing Health Promoting Behaviors Among Latino Immigrants* | | Ineligible condition |
| 68 | *Moore AP, Rivas CA, Harding S, Goff LM. Barriers to following dietary recommendations for type 2 diabetes in patients from UK African and Caribbean communities: A qualitative study* | | Full text unavailable |
| 69 | *Naccashian Z. The impact of diabetes self-management education on glucose management in ethnic Armenians with type 2 diabetes* | | Conference abstract |
| 70 | *Nam S, Song HJ, Park SY. Challenges of diabetes management in immigrant Korean Americans. Diabetes* | | Full text unavailable |
| 71 | *Naranjo DM, Jacobs EA, Fisher L, Hessler D, Fernandez A. Age and glycemic control among low-income Latinos. Journal of immigrant and minority health / Center for Minority Public Health* | | Conference abstract |
| 72 | *Navodia N, Wahoush O, Tang T, Yost J, Ibrahim S, Sherifali D. Culturally tailored self-management interventions for South Asians with type 2 diabetes: a systematic review* | | Ineligible population |
| 73 | *Nct. A Mobile Health Intervention to Reduce Diabetes Disparities in Chinese Americans* | | Ineligible population |
| 74 | *Njeru JW, Wieland ML, Kwete G, Tan EM, Breitkopf CR, Agunwamba AA, et al. Diabetes Mellitus Management Among Patients with Limited English Proficiency: A Systematic Review and Meta-Analysis* | | Protocol only |
| 75 | *Ntr. Dialert, effects of a lifestyle intervention in Dutch and Turkish 1st degree relatives of persons with type 2 diabetes, a randomised controlled trial* | | Ineligible population |
| 76 | *Park C, Nam S, Whittemore R. Incorporating Cultural Perspectives into Diabetes Self-Management Programs for East Asian Immigrants: A Mixed-Study Review* | | Protocol only |
| 77 | *Persaud K. Changing the Behavior of the Caribbean Immigrants in Toronto* | | Ineligible population |
| 78 | *Pistulka GM. Type 2 diabetes mellitus and hypertension in middle-aged, first-generation Korean American immigrants: an exploration of everyday life* | | Full text unavailable |
| 79 | [*Pérez-Escamilla*](https://pubmed.ncbi.nlm.nih.gov/?term=P%C3%A9rez-Escamilla+R&cauthor_id=21367946)*R. Acculturation, nutrition, and health disparities in Latinos. American Journal of Clinical Nutrition* | | Full text unavailable |
| 80 | *Ramal E, Petersen AB, Ingram KM, Champlin AM. Factors that influence diabetes self-management in Hispanics living in low socioeconomic neighborhoods in San Bernardino, California. Journal of immigrant and minority health / Center for Minority Public Health* | | Ineligible population |
| 81 | *Rawal L, Sahle BW, Smith BJ, Kanda K, Owusu-Addo E, Renzaho AMN. Lifestyle interventions for type 2 diabetes management among migrants and ethnic minorities living in industrialized countries: A systematic review and meta-analyses* | | Ineligible population |
| 82 | *Rosas LG, Lv N, Lewis MA, Venditti EM, Zavella P, Luna V, et al. A latino patient-centered, evidence-based approach to diabetes prevention. Journal of the American Board of Family Medicine* | | Ineligible population |
| 83 | *Sarte AF, Fong M, Yung K, Ng L, Koehn S, Sohal P. Culturally-appropriate prediabetes lifestyle intervention programs: A review of the literature* | | Ineligible phenomena of interest |
| 84 | *Seliger J, Simons AL, Maida CA. Promotores-focused screening and education to improve diabetes awareness and self-care in low-income Latino intergenerational families* | | Conference abstract |

| **Items** | **Title** | **Reason for exclusion** | |
| --- | --- | --- | --- |
| 85 | *Zeng B, Sun WJ, Gary RA, Li CW, Liu TT. Towards a conceptual model of diabetes self-management among Chinese immigrants in the United States* | | Ineligible phenomena of interest |
| 86 | *Whittemore R, Vilar-Compte M, Cerda SDL, Delvy R, Jeon S, Burrola-Méndez S, et al. ¡Sí, Yo Puedo Vivir Sano con Diabetes! A Self-Management Randomized Controlled Pilot Trial for Low-Income Adults with Type 2 Diabetes in Mexico City* | | Ineligible population |
| 87 | *Hosler AS, Solanki MN, Savadatti S. Assessing Needs and Feasibility of Diabetes Self-management Coaching at Faith-Based Organizations for Indo-Guyanese Immigrants* | | Ineligible population |
| 88 | *Huffman FG, Vaccaro JA, Gundupalli D, Zarini GG, Dixon Z. Acculturation and diabetes self-management of Cuban Americans: is age a protective factor?* | | Ineligible population |
| In89 | *Sharma A, Stuckey h, Mendez-Miller M, Cuffee Y, Juris AJ, MxCall-Hosenfeld JS. The influence of patriarchy on Nepali-speaking Bhutanese women's diabetes self-management* | | Ineligible population |
| 90 | *Osborn B, Albrecht SS, Fleischer NL, Ro A. Food insecurity, diabetes, and perceived diabetes self-management among Latinos in California: Differences by nativity and duration of residence* | | Ineligible population |
| 91 | *Hu J, Amirehsani K, Wallace DC, Letvak S. Perceptions of barriers in managing diabetes: perspectives of Hispanic immigrant patients and family members* | | Ineligible population |
| 92 | *Hu J, Wallace DC, McCoy TP, Amirehsani KA. A family-based diabetes intervention for Hispanic adults and their family members* | | Ineligible population |
| 93 | *Tatara N, Hammer HL, Andreassen HK, Mirkovic J, Kjøllesdal MKR. The Association Between Commonly Investigated User Factors and Various Types of eHealth Use for Self-Care of Type 2 Diabetes: Case of First-Generation Immigrants from Pakistan in the Oslo Area, Norway* | | Ineligible population |
| 94 | *Tseng J, Halperin L, Ritholz MD, Hsu WC. Perceptions and management of psychosocial factors affecting type 2 diabetes mellitus in Chinese Americans* | | Ineligible population |
| 95 | *Telle-Hjellset V, KjÃ¸llesdal MKR, BjÃ¸rge B, Holmboe-Ottesen G, Wandel M, Birkeland KI, et al. The InnvaDiab-DE-PLAN study: a randomised controlled trial with a culturally adapted education programme improved the risk profile for type 2 diabetes in Pakistani immigrant women* | | Ineligible population |
| 96 | *Sullivan LV, Hicks P, Salazar G, Robinson CK. Patient beliefs and sense of control among Spanish-speaking patients with diabetes in northeast Colorado* | | Ineligible population |
| 97 | *Wieland ML, Njeru JW, Hanza MM, Boehm D, Singh D, Yawn B, et al. Stories for change: Pilot feasibility project of a diabetes digital storytelling intervention for refugee and immigrant adults with type 2 diabetes* | | Ineligible population |
| 98 | *Sun AC, Tsoh JY, Saw A, Chan JL, Cheng JW. Effectiveness of a Culturally Tailored Diabetes Self-Management Program for Chinese Americans* | | Ineligible population |
| 99 | *Uitewaal P, Hoes A, Thomas S. Diabetes education on Turkish immigrant diabetics: predictors of compliance.* | | Ineligible population |
| 100 | *Seligman R, Valdovinos MD, Fernandez A, Jacobs EA. Self-care and Subjectivity among Mexican Diabetes Patients in the United States* | | Ineligible population |
| 101 | *Lanting LC, Joung IM, Vogel I, Bootsma AH, Lamberts SWJ, Mackenbach JP. Ethnic differences in outcomes of diabetes care and the role of self-management behavior* | | Ineligible population |
| 102 | *Jowsey T, Gillespie J, Aspin C. Effective communication is crucial to self-management: The experiences of immigrants to Australia living with diabetes* | | Ineligible population |
| 103 | *Lee H, Woo BKP.* *Perceptions of and Attitudes Toward Diabetes Among Chinese Americans* | | Ineligible population |
| 104 | *Joo Ly, Liu MF. Experience of culturally-tailored diabetes interventions for Ethnic minorities: a qualitative systematic review* | | Ineligible condition |
| 105 | *Alzubaidi H, Sulieman H, Namara KC, Samorinha C, Browning C. The relationship between diabetes distress, medication taking, glycaemic control and self-management.* | | Duplicate study |
| 106 | *Fellx H, Rowland B, Long CR, narcisse MR, Piel m, Goulden PA, McElfish PA. Diabetes self-care behaviors among Marshallese adults living in the United States* | | Ineligible population |
| 107 | *Robert G, Katherine Y, Li-Jung L, Fredric A, Peter B, Penny L, Jose H, Synchronous provider visit and self-management education improves glycemic control in hispanic patients with long-standing type 2 diabetes* | | Ineligible population |
| 108 | *Grant RC, Retnakaran. Healthcare, self-care, and health status of immigrants and non-immigrants with type 2 diabetes in the Canadian Community Health Surveys* | | Ineligible population |
| 109 | *Tatara N, Hammer HL, Andreassen HK, Correction: The Association Between Commonly Investigated User Factors and Various Types of eHealth Use for Self-Care of Type 2 Diabetes: Case of First-Generation Immigrants From Pakistan in the Oslo Area, Norway.* | | Duplicate study |
| 110 | *Venkatesh S. Acculturation and associated factors in relation to glycemic control and self-management of diabetes in Asian Indian adults in the U.S.* | | Duplicate study |
| 111 | *Sonsona JB. Factors Influencing Diabetes Self-Management of Filipino Americans with Type 2 Diabetes Mellitus: A Holistic Approach* | | Full text unavailable |
| 112 | *Yeh MC, Lau W, Chen S, Wong A, Tung HJ, Ma GX, Wylie-Roserr J. Adaptation of diabetes prevention program for Chinese Americans - a qualitative study.* | | Ineligible population |

| **Items** | **Title** | **Reason for exclusion** | |
| --- | --- | --- | --- |
| 113 | *Weiler D, Crist JD. Diabetes self-management in the migrant Latino population* | | Ineligible population |
| 114 | *Zeng B, Sun W, Gary RA, Li CW, Liu TT. Towards a Conceptual Model of Diabetes Self-Management among Chinese Immigrants in the United States* | | Ineligible population |
| 115 | *Borovoy A, Hine J. Managing the unmanageable: elderly Russian Jewish Émigrés and the biomedical culture of diabetes care* | | Ineligible population |
| 116 | *Osborn B, Albrecht SS, Fleischer NL, Ro A.Food insecurity, diabetes, and perceived diabetes self-management among Latinos in California: Differences by nativity and duration of residence* | | Ineligible population |
| 117 | *Amirehsani KA. Self-care expressions, patterns, and practices of Latinos/Hispanics for the management of type 2 diabetes* | | Duplicated study |
| 118 | *Smith-miller CA, Berry DC. Miller CT. Gender differences and their influences on T2DM self-management among Spanish-speaking Latinx immigrants* | | Ineligible population |
| 119 | *Wang CY, Abbott LJ. Development of a community-based diabetes and hypertension preventive program.* | | Ineligible population |
