# Supplementary material for: Cultural Influences, Experiences and Interventions Targeting Self‐Management Behaviours for Prediabetes or Type 2 Diabetes in First‐Generation Immigrants: A Scoping Review
Source: J Adv Nurs. 2024 Nov 21;81(6):2929–45. doi: 10.1111/jan.16621 (PMC12080094; doi:10.1111/jan.16621)
Supplement: Supplementary file 4 — Table S3. [file JAN-81-2929-s001.docx]

| **Supplementary table 3-a: Characteristics of included quantitative studies (N=35)** | | | | | | | | | | | | | |
| --- | --- | --- | --- | --- | --- | --- | --- | --- | --- | --- | --- | --- | --- |
| **Author,**  **year** | **Study aims** | **Study design,**  **Setting,**  **Location** | **Sample size,**  **Sampling method** | **Population** | | | **Country of origin,**  **Primary language** | **Country of residence,**  **Second language** | **Ethnicity (%)** | | | | **Duration of**  **immigration**  ***Mean (SD) or Range (year)*** |
|  |  |  |  | **Age**  ***Mean (SD) or Range (year)*** | **Female**  **sex (%)** | **Diagnosis,**  **Disease duration**  ***Mean (SD) or Range (year)*** |  |  |  |  |  |  |  |
| Alzubaidi,2015 ^1^ | To compare illness and treatment perceptions between Arabic-speaking immigrants (ASPs) and Caucasian English-speaking people (ESPs) with T2D and explore the relationships between these beliefs and adherence to self-care activities. | Multicentre cross-sectional, in hospitals, medical practices and  community settings,  in the Melbourne metropolitan area and rural Victoria,  Australia | N=701  (ASPs=392, ESPs=309) | ASPs vs. ESPs:  58.04 (8.00)  vs.  60.41 (9.54) | 49.9% vs. 46.1% | T2D,  7.18(4.63) vs. 10.51(8.39) | 1)ASPs: Iraq 18.3%,  Egypt 24.7%, UAE 0.8%, Lebanon 32.6%, Syria 3.6%, Somalia 12%, Jordan 3.1%, Kuwait 1.3%, Palestine 1.8%; Arabic.  2)ESPs: Australia 76%, UK 0.65%, Ireland 0.97%, New Zealand 1.63%, Europe 20%; English. | Australia,  English | | ASPs: Middle Eastern people  ESPs: Anglo-Celtic or other Caucasian identity. | | N/A | |
| Alzubaidi, 2022 ^2^ | To assess and compare diabetes distress and its association with medication-taking behaviours, glycemic control, self-management, and psychosocial factors among first-generation ASPs and ESPs with diabetes and determine diabetes distress predictors. | Multicentre cross-sectional, in metropolitan and rural Victoria,  Australia | N= 696  (ASPs=392, ESPs=304) | 59.1(8.9) | 48.6% | T2D,  8.7(6.8) | 1)ASPs: the Arab League countries; Arabic.  2) ESPs: North America,  Australia, UK, Ireland, or New Zealand; English | Australia, English | | N/A | N/A | | |
| Amirehsani,2011^3^ | To explore the use of traditional, complementary, and alternative (TCA), self-care expressions, patterns, and practices for T2D management among Latinos/Hispanics living in a non-border community. | Cross-sectional, in multiple  community settings, in North Carolina | N=75,  convenience sampling | 47 or (25-73) | 66.7% | Self-reported T2D,  7.59 | Mexico 85.3%, Puerto Rican, Cuban, Dominican, Central or South American, or other Spanish origin;  Spanish | US,  English | | Latino/Hispanic | 16 (7.22) | | |
| Chesla,  2013 ^4^ | To improve diabetes care processes and outcomes, with a goal of developing culturally accessible and acceptable diabetes care for Chinese immigrants; to adapt and test a small group intervention in this population, using community-based participatory research (CBPR) strategies. | Single-group pre- and post-test, in federally qualified health centres in Chinatown, San Francisco. | N=145,  convenience sampling | 64.4(9.37) or  (36-83) | 59% | T2D,  7.91(6.63) or (1.1–32) | China,  Cantonese | US,  English | | Chinese | 7.96 (11.91)  or (0.1–52) | | |
| Chesla,  2014 ^5^ | To examine how Chinese American women with T2D differed from their male counterparts on psychosocial factors associated with disease management, and whether they responded differently to a behavioural intervention. | Single-cohort trial, in federally qualified health centres in Chinatown, San Francisco. | N=145,  convenience sampling | 61(9.49) | 56% | T2D,  8.2(6.97) | Mainland China 57%,  Hong Kong 40%;  Cantonese | US,  English | | Chinese | 18(11.9) | | |
| Choi,  2012 ^6^ | To assess the effectiveness, feasibility, and acceptability of a short-duration culturally tailored, community-based diabetes self-management program (CTCDSP) for Korean immigrants with T2D. | Pre- and post-test, at a Korean community on the  West Coast. | N=41 | 70.3(8.4) or  (30-87) | 53.7% | T2D,  8.9 (8.6) | Korea,  Korean | US,  English | | Korean | 27 | | |
| Choi,  2019 ^7^ | To examine the influence of diet family support on glucose outcome in Korean immigrants with T2D. | Cross-sectional, at community sites in a West Coast Koreatown. | N=143 | 64.5(11.9) or  (30-80) | 51.75% | T2D,  5.96(5.20) | Korea,  Korean | US,  English | | Korean | 23.2(8.7) | | |
| Choi,  2020 ^8^ | To investigate factors associated with receiving diabetes spousal support among Korean immigrants with T2D, and to test whether culture-specific factor is a significant predictor of spousal support receipt in this group. | Cross-sectional, at a Korean community in southern California. | N=136,  convenience sampling | 70.97(8.11) or (46-89) | 33% | T2D,  12y | Korea,  Korean | US,  English | | Korean | 29.46 (10.09) | | |
| Coffman,  2012 ^9^ | To examine the extent of undetected and untreated T2D in recent Latino immigrants, and to look at the relationships among diabetes symptoms, blood glucose level, diabetes knowledge, health literacy, and health care use. | Cohort study, in one Latino service agency, at community education centre and Latino health fairs in South-eastern US. | N=144 | 43.6(12.6) | 78.5% | Self-reported T2D,  < 5 y | Mexico 57.6%,  Central 13.9%,  South America 23.6%; Spanish | US,  English | | Latinos | 9.8(8.8) | | |
| Eh,  2016 ^10^ | To investigate the influence of cultural and other factors on DSM behaviours among Australian Chinese immigrants with T2D. | Cross-sectional, at the community and diabetes centre of Hospital, Sydney. | N=139,  convenience sampling | 62.9(11.6) | 54.4% | T2D,  12.5(8–20) | Mainland China 44.6%, Hong Kong 26.6%, Others 28.7% (Taiwan, Singapore, Malaysia, Vietnam, Philippines, Indonesia). | Australia, English | | Chinese | N/A | | |
| Hempler,  2023 ^11^ | To examine the impact of a co-created culturally sensitive DSMES intervention on the physical and mental health of immigrants with T2D. | Pre- and post-test, at a diabetes centre in Copenhagen, Denmark. | N=97 | 59 | 73.3% | T2D,  < 1 y (20.0%),  2–3 y (12.6%),  4–5 y (10.5%),  6–10 y (16.8%),  > 10 y (40.0%). | Arabic 54.6%, Urdu35.1%, Turkish10.3% | Denmark,  Danish | | Urdu, Arabic and Turkish-speaking immigrants | 30.1(11.5) | | |
| Ho,  2020 ^12^ | To develop and test feasibility of an integrative nutritional counselling (INC) program that combines Chinese medicine principles with biomedical nutrition standards. | A pilot two-arm cluster RCT, at a public health centre, San Francisco | N=18  (Intervention=11, Control=7) | Intervention:  61.1(10.5); Control:  63.2(8.7) | 83.3% | Self-reported T2D or Prediabetes | Mainland China77.8%, Hong Kong5.6%, other16.7%;  Cantonese | US,  English | | Chinese | N/A | | |
| Hu,  2022 ^13^ | To examine mobile device ownership, current use, and interest in mHealth interventions among Chinese immigrants with T2D. | Cross-sectional, in  community centres in Chinatown, New York. | N=91 | 70(11) | 63% | Self-report T2D | China;  Mandarin, Cantonese. | US,  English | | Chinese | 19(14) | | |
| Hu & Islam, 2022 ^14^ | To examine the feasibility and acceptability and to test the potential efficacy of a social media based DSME intervention among low-income Chinese immigrants with T2D. | Single group pre- and post-test, in New York. | N=30 | 61 (7) | 70% | Self-report T2D,  9.0 (7.0) | China,  Mandarin | US,  English | | Chinese | 13(7.0) | | |
| Huang,  2022 ^15^ | To examine the role of illness perceptions in the relationship between emotional distress and self-efficacy among Chinese Americans with T2D. | Cross-sectional | N=155 | 69.07 (0.75) or (31-95) | 52.9% | T2D,  13.41 (10.21) or (0-48) | Chinese-speaking countries; Mandarin, Taiwanese, Cantonese. | US,  English | | Chinese | 28.55 (12.84)  or (1-57) | | |
| Hyman,  2017 ^16^ | To examine provider- and patient-related factors associated with DSM among recent immigrants. | Cross-sectional, at the immigrant communities in Toronto. | N=130 | 51.2 | 55% | Self-reported T2D | Sri Lanka 23.1%, Bangladesh 26.9%, Pakistan 26.9%, China 23.1%. | Canada, English | | Asian immigrants | < 10 | | |
| Hyman,  2014 ^17^ | To explore self-management practices and the use of diabetes information and care among Black-Caribbean immigrants with T2D. | Cross-sectional,  in CHCs, clinics or serving organizations, Toronto. | N=102 (Immigrants=48 Canadian-born=54), convenience sampling | 35-64. Immigrants:  54.6(8.2), Canadian-born :52.3(8.3). | 35% vs.52% | Self-reported T2D | Immigrants: Jamaica50%, Trinidad and Tobago16%, St. Vincentand 10%, Caribbean island countries 14%. | Canada, English | | Immigrants:  Black-Caribbean | N/A | | |
| Hyman,  2012 ^18^ | To explore self-management practices, health services use and information-seeking for T2D care among recent immigrants. | Cross-sectional,  in community and hospital settings, Toronto | N=184 (Immigrants=130, Canadian-born=54),  convenience sampling. | 35-64y.  Immigrants:  51.2,  Canadian-born:52.3. | 55% vs.48% | Self-reported T2D | Immigrants:  Sri Lanka 23.1%, Bangladesh 26.9%, Pakistan26.9%, China3.1%. | Canada, English | | Asian immigrants | N/A | | |
| Iten,  2014 ^19^ | To investigate the relationship between immigration status and the patient experience of health care, DSM, and clinical outcomes among Mexican immigrants with diabetes. | Secondary analysis from a cross-sectional study and medical record in community clinics in San Francisco and Chicago | N=401  (US-born=124, Immigrants (IM): Documented IM=166, Undocumented IM=111), convenience sample | US-born:  51.8 (13.9),  Documented IM:55.7 (10.8), Undocumented IM 49.8 (12.5) | 51.6% vs. 50% vs. 54.0% | T2D,  11.75 (10.35) vs.  11.27 (9.71) vs.  9.24 (9.92) | Mexico, Spanish | US,  English, | | Mexican | Documented IM:  31.95 (11.63),  Undocumented IM:  14.60 (8.44) | | |
| Jordan,  2010 ^20^ | To examine the diabetes self-care behaviours of Filipino-American adults with T2D. | Cross-sectional | N=192, convenience sampling | 68.3(10.9) | 61.5% | Self-reported T2D,  11.2(8.4) | The Philippines,  Filipino | US,  English | | Filipino | 17.9(10.5) | | |
| Jordan,  2011 ^21^ | To determine the foot self-care practices performed by Filipino-American women with T2D. | Cross-sectional, in Los Angeles and Orange Counties, California. | N=118 | 68.3(10.5) | 100% | Self-reported T2D | The Philippines,  Filipino | US,  English | | Filipino | N/A | | |
| Kim,  2009 ^22^ | To test the efficacy of a culturally tailored T2D management intervention for Korean American immigrants with T2D. | A two-arm RCT, at community-based settings, in Baltimore-Washington. | N=79  (Intervention = 40, Control = 39), stratified sampling | 56.4(7.9) | 44.3% | Uncontrolled T2D | Korea,  Korean | US,  English | | Korean | 53.2% participants > 20 | | |
| Kim,  2015 ^23^ | To test the effectiveness of a culturally tailored behavioural intervention program in an ethnic/linguistic minority group with T2D. | RCT, at a community-based  setting. | N=209  (Intervention=105, Control=104) | 58.7(8.4) | 43.1% | T2D,  8.53(7.17) | Korea,  Korean | US,  English | | Korean | 23.8 (11.0) | | |
| Kim,  2020 ^24^ | To empirically examine the underlying mechanisms of health literacy 's role in diabetes management among Korean American immigrants with T2D. | RCT, at a community-based  setting. | N=209  (Intervention=105, Control=104) | 58.7(8.4) | 40.9% | T2D | Korea,  Korean | US,  English | | Korean | 23.4 (0.1–53.0;  median = 25.1) | | |
| Krieg,  2017 ^25^ | To evaluate the feasibility and effectiveness of a culturally tailored Diabetes Conversation Map TM education intervention. | Pre- and post-test | N=7,  convenience sampling | 52 (12.8) or (35-71) | 71.4% | T2D,  7.4(5.4) or (1-16) | El Salvador 37.5%, Mexico37.5%, Guatemala 12.5%, Honduras 12.5%; Spanish | US,  English | | Hispanic | N/A | | |
| Loya,  2021 ^26^ | To examine a culturally tailored PA intervention. | Pre- and post-test | N=21, convenience sampling | 53.0(11.8) or  (30-75) | 90.5% | T2D or Prediabetes | Mexico,  Spanish | US,  English | | Mexican95.2%,  Dominican4.8% | 14.94 (11.25) or  (0.25-35) | | |
| Mier,  2012 ^27^ | To compare the magnitude of self-care behaviours among US-born older Hispanics with T2D to those Mexico-born; and examine the influence of personal, acculturation, and health indicators on self-care behaviours among this population. | Cross-sectional | N=238  (Mexico-born=179,  US-born=59) | 60-64y (41.9%),  65-74y (42.4%), 75+y (15.7%). | 65.4% | T2D | Mexico,  Spanish | US,  English | | Hispanic | 39.95 (22.29) | | |
| Naccashian, 2014 ^28^ | To investigate the impact of DSM education on glycaemic control and perceptions of empowerment in Armenian immigrants with T2D. | Pre- and post-test, at health day care centres in Glendale or Los Angeles. | N=75,  convenience sampling | 75.33 (6.3) | 78.7% | T2D,  10.65(8.2) | Armenia,  Armenian | US,  English | | Armenians | N/A | | |
| Pettersson,  2017 ^29^ | To compare foreign‐ and Swedish‐born persons with T2D, to study whether there are dissimilarities in diabetes knowledge and to study determinants of knowledge. | Cross-sectional | N=138  (Foreign-born=69, Sweden=69), convenience sampling | Foreign-born:33-90,  Sweden:48-91. | Foreign-  born: 46%, Sweden:  46%. | T2D,  Foreign-born:  <10y (58%),  ≥10 (42%);  Sweden:  <10y (40%),  ≥10 (60%). | 1) Europe: Bosnia=9, Turkey=8, Poland=4, Finland=4, Kosovo=3, Croatia=2, Italy=1.  2)Middle East/outside Europe: Syria=14, Iraq=12, Chile=5, Lebanon=3, Sri Lanka=1, Burma=1, Burundi=1, Somalia=1. | Sweden, Swedish | | European, Middle East immigrants,  Swedish. | Foreign-born:  25 (17) | | |
| Park,  2020 ^30^ | To examine the relationships between health literacy, illness perceptions, and T2D self-management, and to determine if health literacy moderates the relationship between IP and T2D self-management in Korean immigrants with T2D. | Cross-sectional, at Korean churches in Dallas-Fort Worth areas. | N=52,  convenience sampling | 63.13(11.97) | 32.7% | Self-reported T2D,  9.4(8.11) | Korea,  Korean | US,  English | | Korean | 23.29(12.48) | | |
| Rechenberg, 2021 ^31^ | To test the feasibility and acceptability of a language concordant health coaching intervention among Latinx immigrants with T2D and limited English proficiency (LEP). | Feasibility RCT, in federally qualified health centres. | N=17  (Intervention=10; Control= 7) | 51.7 (12.9) | 64.7% | T2D | N/A,  Spanish | US,  English | | Latinx | N/A | | |
| Thabit,  2009 ^32^ | To compare the diabetes self-care profile of immigrant patients (IM) and Irish patients (IR), and to evaluate differences in health literacy between the two cohorts. | Cross-sectional,  from diabetes outpatient service. | N=100  (IM=52, IR=48),  random sample | IM vs.IR:  45.8(11.8) vs. 60.1(11.0) | N/A | T2D,  4.7(3.7) vs. 6.3(5.6). | IM: South Asia 29%, Africa 27%, East Asia13%, Middle East 17%, Eastern Europe 8%, Western Europe 4%, South America 2%.  IR: Irish/Caucasian, English. | Ireland,  English | | IM:  Non-Irish  IR: Irish/Caucasian. | IM<10y | | |
| Vaccaro,  2014 ^33^ | To investigate how ethnicity, perceived family/friend social support, and health behaviours are associated with DSM in minorities. | Cross-sectional | N=405  (Cuban Americans (CA)=174, Haitian (HA)=121, African Americans (AA)=110). | CA:65(12.0),  HA:58.4(9.9),  AA:54.1(10.4). | CA: 62%,  HA: 58%,  AA: 57%. | T2D | CA: Creole,  HA: Spanish,  AA: English | US,  English | | CA, HA, AA | CA >15 y (75.9%,  HA > 15y (58.7%),  AA >15y (100%). | | |
| Wang,  2005 ^34^ | To assess the feasibility, acceptability, and preliminary outcomes of a culturally appropriate diabetes management program among Chinese Americans with T2D. | Pre- and post-test | N=33/50 | 68.8(10.1) or (44-87) | 51.5% | T2D,  9.03(8.74)  or (1-40) | China, Mandarin36.4%, Cantonese57.6%, Taiwanese6.1%. | US,  English | | Chinese | 16.5 (9.3) | | |
| Williams, 2016 ^35^ | To examine the influence of psychosocial adjustment on medication adherence in uninsured Hispanic immigrants with T2D. | Cohort study, at the clinics associated with an academic  institution in Northern Virginia. | N=70,  convenience sampling | 53.43 or  (40-64) | 68.6% | T2D,  <1y (14.3%),  >1y (85.7%) | America 1.4%, Argentina1.4%, Bolivia 7.1%, Colombia 1.4%, Ecuado 4.3%, El Salvador 32.9%, Guatemala 12.9%, Honduras 12.9%, Lima-Peru 1.4%, Mexico 17.1%, Nicaragua1.4%, Peru 4.3%, Puerto Rico 1.4%; Spanish | US,  English | | Hispanic | 1-59 | | |

Notes: diabetes self-management, DSM; diabetes self-management education and support (DSMES); diabetes self-management education (DSME); type 2 diabetes (T2D); United States (US); not applicable (N/A)

| **Supplementary table 3-a: Characteristics of included quantitative studies (N=35) (continued)** | | | | | | |
| --- | --- | --- | --- | --- | --- | --- |
| **Author (year)** | **Concept:**  **Content of Self-management** | **Theoretical framework** | **Primary outcomes** | **Dara collection method** | **Data analysis** | **Major findings related to SM behaviours** |
| Alzubaidi 2015 ^1^ | Diet, exercise, blood glucose testing, foot care, smoking | N/A | 1. Illness perceptions.  2. Treatment perceptions.  3. Diabetes self-care activities.  4. Medication adherence.  5. HbA1c, BP, and lipid levels. | Questionnaires | Chi-square test; Student’s  t-test; Wilcoxon rank-sum tests; Pearson’s correlation; Spearman’s  rank correlation coefficient; ANOVA. | Compared to ESPs, ASPs had significantly poorer adherence levels to all aspects of DSM: diet, exercise, blood glucose testing and foot care. |
| Alzubaidi, 2022 ^2^ | Dietary behaviours, medication adherence, medication underuse, exercise, blood glucose testing, foot care, smoking; self-efficacy. | N/A | 1. Medication adherence.  2. Diabetes self‑management.  3. Health literacy and self‑efficacy.  4. Diabetes distress.  5. HbA1c level, BP, and lipid panel. | Questionnaires | Pearson Chi-square test; Pearson's Correlation (r); Independent-samples T-Test; one-way ANOVA; Multiple linear regression. | Compared with ESPs, ASPs had higher diabetes distress, lower medication adherence, worse self-management and glycaemic control, and poorer health and clinical profile. Higher diabetes distress in ASPs was associated with cost-related medication underuse and lower adherence to exercise, younger age, lower education level, unemployment, lower self-efficacy, and inadequate glycaemic control. |
| Amirehsani,2011 ^3^ | TCA culturally based self-care practices: the use of herbal remedies, faith-based interventions, consulting traditional healers, and the practice of self-medication/self-prescription without seeking medical advice. | Leininger ‘s Culture Care Diversity and Universality Theory; Leininger ‘s Sunrise Enabler | 1. The use of herbal remedies, faith-based interventions,  consulting traditional healers, and the practices of self-medication/self-prescription.  2. The association of TCA self-care expressions, patterns, and practices to glycaemic control and factors associated with the use of TCA self-care expressions, patterns, and  Practices. | Questionnaires; Face-to-face interviews; open-ended questions | A priori power analysis; descriptive statistics, basic content analysis; multiple regression analysis | 1. Diabetes knowledge, self-care, and glycaemic control: Knowledge deficits were noted pertaining to the treatment of hypoglycaemia.  2. Usage of TCA self-care expressions, patterns, and practices herbal remedies: participants reported trusting the combination of herbal remedies and prescription medications more than prescribed medications alone or herbal remedies alone.  3. Using faith-based interventions as part of diabetes self-care. 4. Self-medication/self-prescription practices: Reasons  for using self-medication/self-prescription: to be healed; cultural medicines are not available from US doctors; and a preference for herbal medicines rather than prescription medicines. |
| Chesla,  2013 ^4^ | Chinese Coping Skills Training (CST) session: T2D review skills such as problem-solving, communication, conflict resolution, and stress management were taught and practised via exercises or role-plays. a 4-month delayed treatment for six sessions (1 session/week), 2-hour DVD in Cantonese, lasted 8 months, ten weeks follow up. | Coping Skills Training | 1. Personal domain:1) self-efficacy, 2) diabetes knowledge, 3) bicultural efficacy.  2. Family domain:1) Family diabetes support, 2) Family diabetes conflicts.  3. Diabetes domain:1) diabetes distress,  2) QoL,3) HbA1c.  4. Demographics.  5. Acculturation.  6. Depressive symptoms. | Questionnaires | Multilevel regression models; Mixed Models-linear module; Piecewise time segments | CCST was effective in improving personal, family, and disease management indicators of health, examined immediately and two months post-treatment. Immediate benefits of treatment were evident in the improvement (p<.05) in diabetes self-efficacy, diabetes knowledge, bicultural efficacy, family emotional support, family instrumental support, diabetes distress, and DQoL-Satisfaction. |
| Chesla,  2014 ^5^ | N/A | N/A | 1. Personal domain:1) self-efficacy, 2) diabetes knowledge, 3) bicultural efficacy.  2. Family domain:1) Family diabetes support, 2) Family diabetes conflicts.  3. Diabetes domain:1) diabetes distress,  2) QoL,3) HbA1c.  4. Demographics.  5. Acculturation.  6. Depressive symptoms. | Questionnaires | Multilevel models | 1.72% Participants took oral medications to manage their disease, 16% managed with diet and exercise only, and 12% took insulin.  2. Men are more likely to be prescribed insulin as opposed to lifestyle or oral medications; Women tend to have a greater number of comorbidities and complications and higher HbA1c.  3.Significant gender by treatment interactions were observed for diabetes self-efficacy, bicultural efficacy, family instrumental support, and DQoL–Satisfaction. |
| Choi,  2012 ^6^ | A short-duration CTCDSP (2- sessions): 1. The instruction included pathophysiology of diabetes, complications, treatment modalities, medication, diet, exercise, and self-management strategies; SMBG and how to interpret results. 2. Cultural tailoring.3. Particular attention to diet management. | N/A | 1. Self-management behaviours.  2. Diabetes knowledge.  3. Self-efficacy.  4. Mood.  5. Health status.  6. HbA1c, BP, lipids, BMI, WC, and WHR.  7. Feasibility and acceptability. | Questionnaires; Physical assessment (a finger-stick blood test) | Paired t-tests; chi-square analysis repeated measures  ANOVA; Descriptive statistics | Regarding diabetes management behaviours, participants reported an increase in weekly foot checks and a trend increase in their reported frequency of exercise activities. The CTCDSP is feasible and may be an effective approach to improving self-management and health outcomes in this population. |
| Choi,  2019 ^7^ | Diet family support | N/A | 1. HbA1c.  2. Family support.  3. Demographics, BMI and WHR. | Questionnaires; Physical assessment (a finger stick blood test) | Descriptive statistics; the chi-square test; Bivariate analyses; a multiple linear regression | Family support, specific to diet, is significantly associated with glucose outcomes in Korean immigrants with T2D. More perceived family support was associated with better glucose control. The positive impact of family support on glucose outcome was significantly stronger in men than in women, even after other factors were considered. |
| Choi,  2020 ^8^ | Diet (general and specific), exercise, blood glucose testing, and medication adherence. | N/A | 1. Personal characteristics: 1) Quality of marriage,2) Depression.  2. Duration of diabetes, HbA1c, insulin use.  3. DSM.  4. Diabetes worries/concerns.  5. Self-disclosure of diabetes distress.  6. Demographics. | Questionnaires | Descriptive statistics, correlations, and hierarchical multivariable linear regression models, sensitivity analysis | Among Korean immigrants with T2D, education level, years spent in the US, glucose control status, DSM level, diabetes worries/concerns, and disclosure of diabetes distress were significant predictors of receiving spousal support. Interventions to improve diabetes spousal/family support receipt among Korean immigrants are likely to have the higher impact when this unique cultural factor is integrated. |
| Coffman,  2012 ^9^ | Formal diabetes education classes | N/A | 1. Diabetes symptoms.  2. Diabetes knowledge.  3. Health literacy .  4. Healthcare use.  5.HbAlc. | Structured interviews; Questionnaires | Pearson's correlations; Multiple regression; Residual diagnostics (scatterplots and qq-plots) | Most of these participants' diseases were not being adequately monitored by a medical professional, and they may not have enough information to accurately interpret diabetes symptoms. Adequate health literacy may help Latinos understand, interpret, and act on diabetes symptoms. |
| Eh,  2016 ^10^ | Diet management, exercise management, self-monitoring of blood glucose, foot care, and medication adherence. | N/A | 1. Self-management behaviours.  2. Medication adherence.  3. Acculturation.  4. Traditional beliefs.  5. Demographics. | Self-completion questionnaires | Descriptive statistics; the independent samples  t-test/one-way ANOVA; the Mann–Whitney U-test /Kruskal–Wallis test; a multiple stepwise  regression | 1. Participants had poor self-management practices generally but moderate medication adherence.  2. Gender, education level and duration of diabetes were predictors of DSM behaviours.  3. Higher levels of acculturation predicted better medication adherence, whereas stronger beliefs in TCM predicted poorer medication adherence. |
| Hempler,  2023 ^11^ | The DSMES weekly 2.5-h group sessions covered diabetes knowledge and complications, mental health, diet, exercise, blood sugar measurement and fasting, as well as cultural competence. | N/A | 1.HbA1c and lipid Profile.  2.Body weight, height, WC, BMI.  3.Demographics and diabetes-related measures.  3.Self-Reported physical and mental health.  4.Health behaviours: smoking habits and alcohol consumption.  5. Self-management activities. | Physical assessment; Questionnaires; Medical records. | Descriptive statistics; Paired t-tests; Wilcoxon signed-rank tests; General linear models | The DSMES intervention developed in a co-creation process was highly effective in improving the health of immigrants with T2D.HbA1c, weight, BMI, self-rated general health, and the self-management activities of a healthy diet and physical activity. |
| Ho,  2020 ^12^ | 1. The DSME include diabetes knowledge, exercise, diet, medications, and individual self-management plans. 2. Integrative nutritional counselling (INC) curriculum: a combination of ADA recommendations and Chinese medicine principles.  Intervention group: DSME+INC; Control group: DSME. | Culture-Cantered Approach (CCA) | 1.Demographics.  2. Acculturation.  3.Beliefs in Chinese/Western medicine.  4.Feasibility and acceptability of the intervention. 5.Dietary self-efficacy, diabetes self-efficacy, and diabetes distress.  6.Dietary adherence.  7. Patient experiences, satisfaction, knowledge, behavioural changes, cultural congruence of the diet, and barriers to diabetes diet management. | Physical assessment;  Questionnaires  Qualitative interviews | Descriptive statistics; Fisher’s exact test; Linear mixed models | 1. Barriers to diabetes diet management: time constraints and lack of transportation.  2. Dietary adherence significantly improved in participants who received the INC curriculum. The similar benefits were observed in knowledge, attitudes, and beliefs among participants in INC and in usual DSME. |
| Hu,  2022 ^13^ | Interest in using mHealth for T2D self-management. | The National Institutes of Health (NIH) Stage Model for Behavioural Intervention Development; the National Cancer Institute’s (NCI) Health Information National Trends Survey (HINTS) framework | 1.Demographics.  2.Technology-related questions: mobile device ownership.  3.Access to technology.  4.Current social media use.  5.Current use of technology for health-related purposes.  6.Interest in using mHealth for T2D management.  7.Family and friends’ involvement and interest in mHealth interventions. | Questionnaires | Descriptive statistics | 1. The current use of technology for health-related issues remained low in older Chinese immigrants with T2D.  2. The primary source for diabetes-related information is healthcare clinicians.  3. The majority (76%) of participants reported a strong interest in future mHealth interventions. |
| Hu & Islam, 2022 ^14^ | The culturally tailored DSME intervention: 1) the DSME videos focus on diabetes education (e.g., basics of diabetes care, diet, physical activity); 2) behavioural counselling techniques (e.g., goal setting, self-reward, problem solving). | The Cultural Adaptation Model, the Ecological Validity Model. | 1. Feasibility and acceptability of the intervention.  2. HbA1c.  3. Self-efficacy.  4. Dietary intake.  5. Physical activity. | Surveys (in -person and via telephone), medical records | Descriptive statistics, paired 2-sided t tests | 1.This DSME intervention is feasible, acceptable, and potentially efficacious in a low-income Chinese immigrant population with T2D.  2.The retention rate was 100% at 3-month follow-up and 97% at 6-month follow-up. All strongly agreed that they preferred this video based DSME over face-to-face visits.  3. There were significant improvements in self-efficacy, dietary, and PA behaviours between baseline and 6 months. |
| Huang.  2022 ^15^ | Diabetes management self-efficacy (DMSE): Diet management, self-monitoring, physical exercise and body weight, medical treatment. | Leventhal’s Self-Regulatory Model | 1. Demographic and clinical information.  2. Acculturation.  3. Diabetes distress .  4. Depressive symptoms.  5. Illness perceptions.  6. Self-Efficacy. | Questionnaires | Descriptive analysis, Pearson correlations, mediation model analyses. | 1.Diabetes distress and depressive symptoms had direct negative effects on self-efficacy.  2. Improved perceptions of treatment control can ameliorate diabetes distress and improve diabetes management self-efficacy among Chinese Americans.  3.All five categories of illness perceptions were moderately correlated with self-efficacy scores. |
| Hyman,  2017 ^16^ | DSM practices: PA, smoking cessation, the consumption of a healthy diet, regular foot care, and weekly self-monitoring of glucose. | N/A | 1. DSM practices.  2. The quality of the provider-patient interaction.  3. BMI, WC, diabetes-related comorbidity, and stress. | Questionnaires; Face-to-face interviews (a computer-assisted personal interviewing methodology) | Univariate logistic regression analysis; Multivariate logistic regression using backward stepwise selection; The Akaike information criterion. | Financial barriers and the quality of the provider-patient relationship were statistically significant predictors of DSM among recent immigrants. |
| Hyman,  2014 ^17^ | DSM practices: daily/weekly glucose check, daily/weekly foot check, smoking, physical activity, reducing dietary carbohydrates, reducing dietary fat. | N/A | 1. Demographics.  2. Acculturation.  3. Beliefs in Chinese/Western medicine.  4. Feasibility and acceptability of the intervention. | Open- and close-ended questions,  a structured questionnaire. | t-test,  Chi-square test,  Fisher’s exact test. | 1. Black-Caribbean immigrants were significantly more likely than the Canadian-born group to engage in recommended DSM practices and receive regular HbA1c and eye screening by a health professional.  2. Immigrant participants were significantly more likely to report receiving diabetes information and care through a CHC and nurses and dieticians than their Canadian-born counterparts.  3.CHCs and allied health professionals play an important role in managing diabetes in the immigrant community and may contribute to this group's favourable DSM profile and access to information and care. |
| Hyman,  2012 ^18^ | DSM practices: daily/weekly glucose check, daily/weekly foot check, smoking, regular physical activity, reducing carbohydrates moderately or a lot. | N/A | 1.Demographics.  2. Self-management practices.  3. Health services use  4.Questions about information-seeking practices. 5.Questions on barriers to accessing health care. | A structured questionnaire | Student's t tests,  Chi-square tests | 1.Compared to the Canadian-born group, recent immigrants were significantly less likely to perform regular blood glucose and foot checks; to use a specialist, alternative provider, and dietician; to report using dieticians, nurses and diabetes organizations as sources of diabetes-related information.  2. Important differences were observed by sex and country of origin.  3. Barriers experienced in accessing health care among immigrants. |
| Iten,  2014 ^19^ | DSM:  diet, exercise, blood-glucose testing, foot care, smoking, medication adherence, and self-efficacy. | N/A | 1. Immigration status.  2. Patient experiences of care-cultural competency.  3. DSM:  1) Diabetes self-care;  2) Self-care barriers/supports;  3) Self-efficacy;  4) Medication adherence.  4. HbA1c, BP, LDL-C, weight. | Questionnaires,  Medical records. | Chi-square tests, t-tests; Multivariate linear and logistic regressions | 1. There are no significant differences in reports of physician communication or in measures of diabetes management between undocumented and documented immigrants.  2. All three groups had similar clinical outcomes in glycaemic, systolic BP, and lipid control.  3. At least in some settings, undocumented Mexican immigrants with diabetes can achieve similar clinical outcomes and DSM behaviours as documented immigrants and US-born Mexican Americans. |
| Jordan,  2010 ^20^ | The diabetes self-care behaviours: dietary habits, exercise, education regimens, and self-monitoring of blood glucose. | The Self-Care Deficit Nursing Theory (SCDNT) of Dorothea Orem | 1. Demographics.  2. Self-care behaviours. | Questionnaires | Descriptive statistics, Earman's rank correlation | 1. Dietary habits: Participants who lived in the US longer, were females, or were older, less educated, older at immigration, and older upon diagnosis of T2D were more likely to follow healthful eating plans.  2. Exercise: males and those with higher education exercised more frequently.  3. Medication regimens: The older FAs, females, who were older at immigration and upon diagnosis of T2D followed their medication regimens.  4. SMBG: Younger FAs and participants who have had T2D for a shorter duration tested their blood glucose less frequently. |
| Jordan,  2011 ^21^ | The foot self-care practice involves washing the feet, drying in between toes, checking the feet, and inspecting the inside of shoes. | N/A | 1. Demographics.  2. The foot self-care practice. | Questionnaires | Descriptive statistics；  Students t-test | Foot self-care practices were less frequently performed by FA women with T2D aged above 65. |
| Kim,  2009 ^22^ | 1. Self-care activities: dietary information, exercise, blood glucose testing, foot care, and smoking.  2. Self-efficacy in diabetes management: making appropriate food choices, exercising regularly, and monitoring blood glucose levels.  3. The intervention includes weekly education sessions for six weeks, home glucose monitoring, and monthly telephone counselling. | N/A | 1.HbA1c, fasting glucose, and lipid batteries.  2. Diabetes knowledge.  3. Self-care activities.  4. Self-efficacy.  5. Depression.  6. QoL.  7. Attitudes toward diabetes. | Structured questionnaires  ; physical assessments | Descriptive statistics; a general linear model. | This intervention using trained bilingual nurse counsellors and self-monitoring of glucose can be effective in improving clinical indicators of diabetes and in increasing participants’ level of diabetes-related knowledge and self-efficacy. |
| Kim,  2015 ^23^ | 1. Self-care activities: medication adherence, diet, SMBG, exercise, foot care, and daily decision-making.  2. Intervention group (2h weekly sessions for six weeks): 1) structured behavioural education programs delivered in a group education format,2) ongoing self-monitoring of glucose, and 3) individualized counselling. Control group: a brief educational brochure. | The Predisposing, Reinforcing, and Enabling Constructs in Education/Environmental Diagnosis and Evaluation (PRECEDE)–Policy, Regulatory, and Organizational Constructs in Educational and Environmental Development (PROCEED) model | 1. Predisposing factors:  1) Lifestyle-related T2D risk factors;  2) Medical history;  3) Depression.  4) Demographics.  2. Enabling factors:  1) Diabetes knowledge;  2) Self-efficacy;  3) The participants’ experience and QoL;  4) Self-care activity. | Structured questionnaires; physical assessments | Parametric tests (e.g., t-tests, chi-square tests); t-tests; Chi-square test; the mixed model of panel data. | This intervention improved the self-efficacy of DSM, diabetes knowledge, and diabetes related QoL. |
| Kim,  2020 ^24^ | 1.The intervention included:1) 2-hour classes (6 weeks) focused on T2D etiology, treatment regimens (medication, diet, exercise, and stress management), health literacy, and communication with healthcare providers. 2)Monthly telephone counselling. 3) Home monitoring of daily blood sugar. | The PRECEDE–PROCEDE model | 1. Health literacy.  2. Self-Efficacy.  3. HbA1c.  4. QoL. | Structured questionnaires; physical assessments | Descriptive analysis (t-test, chi-square); mixed-effects models; maximum likelihood estimation of structural equation models. | 1. The health literacy intervention had a positive influence on self-care skills such as knowledge, self-efficacy, adherence, and, ultimately, glucose control; it improved the overall capacity of  individuals to effectively manage their chronic conditions. 2. Self-care skills and self-efficacy mediate the relationship between health literacy, glucose control and QoL. |
| Krieg,  2017 ^25^ | A culturally tailored DSME program | SCT | 1. Diabetes knowledge.  2. Self-efficacy.  3. Participant satisfaction.  4. Self-rated health.  5. HbA1c. | Questionnaires; physical assessments. | Descriptive statistics; A Wilcoxon Signed Ranks Test | In this culturally tailored DSME program, diabetes-related knowledge improved significantly, while self-efficacy and HbA1c did not improve. |
| Loya,  2021 ^26^ | A culturally tailored physical activity intervention: face-to-face sessions delivered weekly for 6 weeks, with a one 60-min education session and four 45-min sessions. | SCT | 1. Demographics.  2. Self-efficacy.  3. Acculturation.  4. Exercise logs and physical activity recall.  5. Feasibility and acceptability. | Questionnaires | Descriptive statistics; paired-sample t tests; generalized estimating equations. | 1.About 90% participants completed the intervention. This PA intervention has promise as a strategy to enhance PA behaviours in the priority population.  2.Despite participants in this study had low acculturation scores, successfully increased their PA from pre to post intervention.  3.There were no significant changes on self-efficacy from pre- to post intervention. |
| Mier,  2012 ^27^ | Self-care behaviours: diet behaviours, physical activity, and self-glucose testing. | A model of determinants and processes of health behaviour change | 1.Demographics.  2.Acculturation.  3.BMI, the mean number of self-reported chronic condition types, and exposure to diabetes education.  4.Self-care behaviours. | Questionnaires, anthropometric measurements | Pearson’s Chi-squared statistics; t-tests;  Independent binary logistic regression analyses;  The log-likelihood ratio test. | 1.Mexico-born participants lived in the U.S. an average of 31 years and still scored significantly lower in the linguistic acculturation scale than those U.S. born.  2.older age, being male, and having higher education attainment are significant correlates to following daily a healthful eating plan.  3.Being female, more educated, US-born, and receiving diabetes education were associated with PA. Diabetes education was associated with glucose monitoring. |
| Naccashian, 2014 ^28^ | DSME classes (6 weeks,9 h): taking care of your diabetes, medication management, mastering the meal plan, understanding healthy food choices, self-monitoring of blood glucose, exercise, beating the blues and keeping healthy foot and skin practices. | N/A | 1.HbA1c.  2.Empowerment.  3.Acculturation.  4. Demographics. | Questionnaires; medical records | Univariate analysis; a paired t test; multiple regression analyses. | The post-intervention mean DES score was significantly greater than the preintervention mean DES score. The DSME classes can improve diabetes self-care management skills. |
| Pettersson,  2017 ^29^ | Diabetes knowledge | N/A | 1.Diabetes knowledge.  2. Demographics.  3. HbA1c. | Structured interviews (45–60 min) based on questionnaires | Student’s t test, Mann–Whitney U‐test, chi‐squared test, multiple logistic regression analysis | 1.Foreign‐born persons had lower knowledge about diabetes than Swedish‐born persons.  2.There was a relationship between poor knowledge and country of birth, marital status, and employment status. 3.Country of birth was the strongest independent determinant of knowledge about diabetes. The risk of poor knowledge was ten times higher among persons born in the Middle East or in another country outside Europe compared with Swedish‐born persons.  4.Other influencing factors for poor knowledge about diabetes were being not gainfully employed and living alone. |
| Park,  2020 ^30^ | Diet control, physical activity , blood glucose test, and foot exam. | The common-sense model of self-regulation (CSM). | 1.Demographics.  2.Health literacy.  3.Illness Perception.  4.Self-management behaviours. | Questionnaires | Descriptive statistics; the Pearson’s product-moment correlation; the Shapiro-Wilk test; a hierarchical multiple regression. | 1. Differences in T2D self-management were found in sex and employment status.  2. The effect of illness perception on T2D self-management was not different at different values of health literacy. |
| Rechenberg, 2021 ^31^ | Intervention group: standard diabetes care + six biweekly health coaching sessions.  Control group: standard diabetes care + written educational materials. | N/A | 1.Demographics.  2.HbA1c.  3.Anxiety symptoms.  4.Depressive symptom.  5.Patient satisfaction. | Questionnaires; A semi-structured interview; Self-report measures; a medical record review | Descriptive statistics, dependent t- tests. | The language concordant health coaching intervention is both feasible and acceptable to Latinx immigrants with T2D and LEP and resulted in clinically meaningful differences in key diabetes-related outcomes. |
| Thabit,  2009 ^32^ | Patients’ self-care practices, attitudes and beliefs towards diabetes and difficulties with diabetes self-care. | The Common European Framework of Reference for Languages | 1. Demographics.  2. DSM.  3. Health literacy .  4. Plasma glucose, HbA1c, lipid panel, BP, BMI. | Self-report survey, physical assessments, open-ended  questions | Independent samples t-test, chi-square test | 1. Compared with the IR group, IM had poorer glycaemic control and lower health literacy (using REALM), and a significant proportion of IM forgot to monitor their daily blood glucose.  2. Family support is more important among IM in performing daily blood glucose monitoring, taking medications, and following an appropriate meal plan. |
| Vaccaro,  2014 ^33^ | The role of family or friend social support (FSS) in DSM. | Fischer and colleagues’ ecological approach to disease self-management | 1. Participants’ characteristics.  2. Participants’ perception of receiving functional social support.  3. DSM.  4. HbA1c.  5. Self-rated health. | Questionnaires | Descriptive statistics, Spearman’s rho correlations, MANOVA and post hoc analyses, multiple regression models. | 1. FSS, together with ethnicity, may influence DSM.  2. DSM was highest in Haitians- as compared to African Americans, yet Haitian Americans had poorer glycaemic control.  3. Higher FSS scores were associated with higher DSM scores, independent of ethnicity.  4. Several ordinal components of FSS, such as the degree of agreement concerning perceived family listening, nagging, and feeling uncomfortable about diabetes, differed by ethnicity.  5. This study did not find gender differences to be associated with DSM. |
| Wang,  2005 ^34^ | A culturally tailored diabetes education program: dietary education, exercise, medication, self-care, and Chinese-specific activities such as meditation, Tai chi, and Chi-gong. 60 min per session for ten sessions. | The Empowerment Model | 1. Feasibility and acceptability.  2. Diabetes QoL.  3. Weight, BP, and HbA1c. | Questionnaires, physical assessments | Descriptive statistics, Student's t-test, t-test of paired sample statistics. | 1. The attrition rate was 17.5%. 75% of the participants understood the course content and identified and demonstrated various diabetes management skills (70% and 82.5%, respectively).  2. All participants who completed the program were "very satisfied" with the program.  3. Significant improvements in weight loss, BP, mean HbA1c, and DQoL were reported.  4. This culturally tailored diabetes management pilot study could be an effective tool for reducing health disparities in the Chinese American population. |
| Williams, 2016 ^35^ | Medication adherence | The Life Course Health Development (LCHD) Framework | 1. Demographics.  2. Psychosocial adjustment.  3. Medication adherence. | Questionnaires | Descriptive statistics; Stepwise Multinomial Logistic Regression; Pearson’s chi-square. | 1. About 78.5%participants were in the low to medium adherence medication.  2. Psychosocial adjustment has a positive influence on medication adherence in the targeted population. |
| Notes: Arabic-speaking immigrants (ASPs); Caucasian English-speaking people (ESPs); blood pressure (BP); Body Mass Index (BMI); diabetes self-management (DSM); traditional, complementary, and alternative (TCA); culturally tailored, community-based diabetes self-management program (CTCDSP); diabetes self-management education and support (DSMES); diabetes self-management education (DSME); social cognitive theory (SCT); Diabetes Quality of Life (DQoL); Glycated Haemoglobin (HbA1c); Low-density Lipoprotein Cholesterol (LDL-C); limited English proficiency (LEP); type 2 diabetes (T2D); United States (US); not applicable (N/A); Waist Circumference (WC).. | | | | | | |

| **Supplementary table 3-b: Characteristics of included qualitative studies(N=43)** | | | | | | | | | | | | | | | | |
| --- | --- | --- | --- | --- | --- | --- | --- | --- | --- | --- | --- | --- | --- | --- | --- | --- |
| **Author, year** | **Study aims/focus** | **Study design，**  **Sample size,**  **Sampling method** | **Country of origin/residence;**  **Language;**  **Duration of immigration**  ***Mean (SD) or Range (year)*** | **Population** | | | | | | **Theoretical framework** | | **Data collection method** | **Data analysis** | **Main findings/themes related to SM behaviours** | | |
|  |  |  |  | **Age (year)**  ***Mean (SD) or Range (year)*** | | | **Female**  **Sex (%)** | | **Diagnosis, Duration**  ***Mean (SD) or Range (year)*** |  |  |  |  |  |  |  |
| [Abuelmagd, 2019](https://pubmed.ncbi.nlm.nih.gov/?term=Abuelmagd%20W%5BAuthor%5D) ^36^ | To explore the experiences of immigrant Kurdish patients in Oslo, Norway, related to the management of T2D. | Qualitative study,  N=18 | Iraq72.2%, Iran27.8%,  Kurdish;  Norway, Norwegian. | | 51.2 or (40-64) | | | 50 | T2D,  7.3 (0.5-16) | | N/A | Focus group interviews (60 - 90 mins) | Descriptive statistics,  A thematic content analysis with a deductive approach | | | 1. Participants perceived T2D as a burden, and they were afraid of possible complications of the disease.  2. Participants adhered to the medical treatment.  3. Most participants had made changes to their diet.  4. Physical activity received only minimal attention.  5. The general practitioner was the main source of information for the participants. |
| Alzubaidi,  2015 ^37^ | To explore and compare medication-taking experiences and associated issues in Arabic-speaking patients (ASPs) and Caucasian English-speaking patients (CEPs) with T2D in Australia. | Qualitative study,  N=100 (ASPs=60, CEPs=40),  purposive sampling | ASPs:  Lebanon38%,  Jordan7%,  Iraq18%, Syria5%,  Egypt32%,  Arabic.  CEPs:  North America, Australia or the UK, Ireland, New Zealand,  English;  Australia, English;  8 or (3-18). | | ASPs vs. CEPs:  57 or (35-68) vs.  60 or (54-69) | | | 63 vs.60 | T2D,  9 or (1-17) vs.  7 or (3-14) | | N/A | Questionnaire,  medical records,  face-to-face semi-structured individual interviews(n=28); group  interviews (n=72,14 groups) | Constant comparative method | | | 1.ASPs consistently reported failing to conform to their prescribed diabetes treatment regimen.  2. Issues associated with medicine taking:1) Participants’ knowledge.2) Cultural beliefs and views on medicines.3) Communication between participants and healthcare professionals.  3.Medicine adherence among ASPS is related to their experiences with current hypoglycaemic medications, experiences with the disease itself, and experiences of people with a close relationship to the participant. |
| Alzubaidi  ,2017 ^38^ | To explore a new model for DSM support in Arabic-speaking migrants. | Qualitative study,  N=60 | Lebanon 38%, Jordan 7%,  Iraq18%,  Syria5%,  Egypt32%,  Arabic;  Australia, English;  8 or (3-18) | | 57 or (35–68) | | | 63 | T2D,  9 or (1-17) | | N/A | Face-to-face semi-structured individual interviews (n=14),  focus groups (n=46), open-ended questions (30-110 mins). | Descriptive statistics, a thematic analysis | | Themes:  1. Reflections on the journey with diabetes.  2. Preferred delivery modalities.  3. Preferred content. | |
| Baghikar, 2019 ^39^ | To explore barriers and facilitators to adherence among low-income, urban Latinos with T2D. | Qualitative study,  N =27,  convenience sampling | Mexico92.6%,other origin7.4%, Spanish;  US, English;  28(15) | | 57 | | | 81 | Self-reported T2D,  8.8y | | Self-Determination Theory; The Social Ecological Model | Semi-structured interviews, face-to-face, in-depth interviews  (45–60 mins) | A modified template approach | | 1.The participants expressed the desire to control diabetes with lifestyle instead of medication.  2.Unwanted effects of medication and concerns regarding utility were important factors that decreased adherence.  3. Barriers and facilitators to adherence. | |
| Brunk,  2017 ^40^ | To assess the feasibility of adapting a patient-centred educational intervention for T2D self-management for a Hispanic population with low health literacy skills. | Phenomenological descriptive qualitative study, N =9  (patient=8, family member=1) | Mexico 75%, El Salvador 25%; Spanish;  US, English;  (1-30) | | 30-66 | | | Patient:50 | T2D,  1-7y. | | Patient-centred model of DSM assessment and intervention | Focus groups interviews (120 mins) | A hermeneutical phenomenology approach | | Participants shared feedback clustered around four themes: information and knowledge about T2D, motivation and barriers to changing behaviours, experiences with new self-management behaviours, and personal responsibility for disease management. | |
| Barbara, 2013 ^41^ | To identify issues in DSM in an Australian Maltese community with T2D, and to identify opportunities for community pharmacies to offer self-management support to these population. | Qualitative study,  N=24 | Italy, Italian;  Australia, English;  >50y | | 73 or (54-95) | | | 58.3 | T2D,  <5y (8.3%),  6-10y (33.3%),  >10y (58.3%). | | The Airhihenbuwa PEN-3 cultural framework model | Open-ended  Questions,  questionnaire, semi-structured interviews (30–45 mins) | Content-analysed and iteratively and inductively coded into themes | | 1.Themes: 1) Diabetes knowledge; 2) Self-management behaviours;  3)Cultural predictors of self-management behaviours;  4)Interest in pharmacy diabetes care support.  2.Barriers to care: poor written literacy and limited access to diabetes education.  3.Enablers: attitudes towards financial independence and social integration while nurturers included family and community support. | |
| Barko,  2011 ^42^ | To compare perceived symptoms of T2D and self-management strategies between Russian-speaking Slavic immigrants (RSIs) and non-Hispanic, non-immigrant White American women (NNW). | Qualitative descriptive study,  N =20  (RSIs=10, NNW=10),  convenience sampling | Russia, Slavic languages;  US, English. | | 66.01 or  (48-81) | | | 100 | T2D,  RSIs:  9.2(5.47) or  (5-20)  NNW:  11(9.17) or  (1-28) | | Explanatory Model of Illness | Individual interviews, open-ended questions | A thematic analysis | | 1.Described symptoms related to diabetes.  2.The non-immigrant women used more complex DSM techniques compared with the Slavic group.  3.Identified diabetes educational needs. | |
| Carolan-Olah, 2018 ^43^ | To evaluate elderly Italian migrants’ experience of diabetes care and factors that may contribute to local health service use. | Qualitative study,  N=13 | Italy, Italian;  Australia, English;  >50 y. | | 74 or (68-85) | | | 38.5 | T2D,  2-5 y (23.1%),  > 5 y (76.9%). | | N/A | Focus groups interviews(n=3) | A thematic analysis | | 1.Themes:1) the value of health;  2) the impact of diabetes: social impact, physical impact, psychological effects;3) making changes;4) managing diabetes;5) access to information and services. | |
| Cha,  2012 ^44^ | To explore potential factors affecting the SMBs of Korean immigrants with T2D | Cross-cultural qualitative study, N =20 | Korea, Korean;  US, English;  23.6 (9.7) . | | 64.5(11.6) or (37-77) | | | 55 | T2D,  52.5(12.3) | | N/A | A self-reported questionnaire, face-to-face, in-depth, semi- structured interviews (45 -60 mins) | Conventional content analysis | | Three major ideas:  1. Issues on treatment regimen related to medications and diet:1) managing medication “my way”;2) struggling to follow dietary recommendations.  2. Resources that helped or hindered ability to manage diabetes: time and community resources; health insurance; transportation.  3. The physician-patient relationship: desire to become a partner; desire to be seen by a specialist. | |
| Choi.,  2015 ^45^ | To identify domains of spousal support in T2D self-management, compare the domains of spousal support expressed by diabetic patient participants with those of their spouses, and explore perceptions of offering and receiving spousal support within this group. | Qualitative study, N=33  (Patient=16, Spouse=17), purposive sampling | Korea, Korean;  US, English;  Patient:  29.1(9.15),  Spouse:  25.9 (9.93). | | Patient vs. Spouse:  68.1(7.96) or  (60-81) vs.  74.4(3.98) or (66 -83). | | | 50 vs. 47.1 | Patient: T2D,  16.8(23.7) | | N/A | Focus groups interviews (90- 120mins) | Qualitative content analysis | | 1.Domains of spousal support in DSM.  2.Responses to spousal support and suggestions for good spousal support:  1) Support/Advice not listened to;  2) Differences in spousal support style/support should be individualized;  3)Diabetes care is teamwork between husband and wife. | |
| Chun,  2011 ^46^ | To articulate the complex ways in which acculturation affects diabetes management and perceived health for first-generation Chinese immigrants in the U.S. | Qualitative study,  N =59  (Informant group=40,  Respondent group=19),  convenience sampling | Informants:  Mainland China 55%, Hong Kong 45%, Cantonese.  Respondents:  Mainland China 46%, Hong Kong 54%, Cantonese;  US, English;  Informants:  14.7 (13.6),  Respondents:  11.8 (12). | | Informants: 62(9.2), Respondents:  60 (9.4). | | | Informants:60, Respondents: 46. | Informants: T2D: 8.4 (5.9).  Respondents: T2D: 6.2(4.2). | | N/A | Semi-structured interviews (2 hr) in individual, couple and group contexts. | Narrative and thematic analyses | | Themes in patients’ and spouses’ acculturation experiences:  1. Utilizing health care/  2. Maintaining family relations and roles.  3. Establishing community ties and groundedness in the US. | |
| Chesla,  2009 ^47^ | To describe cultural and family challenges to illness management in foreign-born Chinese American patients with T2D and their spouses. | Interpretive comparative interview study, Informants= 40; Respondents = 19,  convenience sampling | Informants: mainland China 55%, Hongkong 45%.  Respondents: mainland China 46%, Hong Kong 54%; Cantonese;  US, English; Informants:15 (13.4); Respondents:11.8 (12). | | Informants:  62 (9.2).  Respondents (13 patients, 6 spouses):  60(9.4) | | | Informants: patient: 60.  Respondents: patient 4 | Informants: T2D: 8.4(5.9)  Respondents: T2D:  6.2 (4.2) | | N/A | Multiple semi structured interviews | Interpretive narrative, thematic analysis | | Themes of cultural and family challenges to diabetes management: 1) diabetes symptoms challenged family harmony;  2) dietary prescriptions challenged food beliefs and practices;  3) disease management requirements challenged established family role responsibilities. | |
| Chun,  2004 ^48^ | To highlight culturally unique experiences and responses to T2D among Chinese immigrant families. | Interpretive phenomenology study,  Patient=13, Spouse=7, convenience sampling | China, Cantonese/Mandarin;  US, English;  Patient:14.11(10.33), Spouse:19.00(13.13). | | Patient:  60.85 (7.5),  Spouse:  59.57 (8.77) | | | Patient: 30.8, Spouse: 42.9. | Patient: T2D or Prediabetes, 6.54 (4.86) | | N/A | Group interviews | Interpretive narrative | | Five primary cultural considerations in diabetes management: (1) the conceptualization of diabetes, illness and health, (2) the significance and meaning of food, (3) exercise and physical activity (4) perceptions of Chinese and Western medicines, and (5) effects of the disease on family dynamics. | |
| Deng,  2019 ^49^ | To develop a nutritional intervention for Chinese immigrants with diabetes and define current deficiencies and identify modifiable factors and mechanisms of change. | Qualitative study,  N=13 | Mainland China 50%, Taiwan 14%, Hong Kong 14%, Southeast Asia 21%, Mandarin;  Canada, English;  25(17) | | 63(15) | | | 61.5 | T2D or prediabetes,  7.5(7.8) | | The conceptual model of DSM among Chinese Immigrants. | A one-on-one, semi-structured interview (40-mins), questionnaire | Descriptive statistics, a thematic analysis | | Themes:  1.Available resources for diabetes dietary management:  1)Barriers: a lack of culturally relevant, language.  2)Facilitator: social support. | |
| Fleming,  2008 ^50^ | To explore the influence of culture on T2D self-management in Gujarati Muslim immigrates. | Case study, N=5, convenience sampling | Uganda40%,  Gujarat 60%;  UK, English. | | 55–72 | | | 0 | T2D | | N/A | Interviews;  Participant observation. | Topic and analytic coding | | Themes:  1) Past experiences and socio-economic factors.  2) Social and gendered roles.  3) Personal choice and contextual factors. | |
| Fagerli,  2005 ^51^ | To explore how ethnic minority persons with diabetes experience dietary advice given by Norwegian health-workers. | Qualitative study,  N=26 (Patient=15, health-workers=12), purposeful sampling | Pakistan, Pakistani;  Norway, Norwegian;  (13-30) | | Patient:38-66 | | | Patient: 73.3 | T2D | | N/A | Semi-structured interviews (45-210 min) | Giorgi's phenomenologically-inspired method | | Experience of constraints related to communication problems.  Struggles of grasping the meaning of the advice. | |
| Guel,  2011 ^52^ | To explore Turkish migrants' experiences with diabetes are, the role of family in shaping responses to chronic illness. | Qualitative study, N=24  (Patients=7,  Health  professionals=  17),  purposive sampling | Turkey, Turkish;  German, Germany. | | Patient:40-65 | | | Patient:  85.7 | T2D:  4-10 | | Social Practice Theory | Narrative, open-ended interviews and conversations (several hours), a semi-structured, flexible interview(20-60mins). | The ethnographic analysis | | 1.Family and other social relationships are inherently tied to efforts of diabetes self-care.  2.Family shapes Turkish Berliners' experience with diabetes in several ways.  3.Constant inter-generational exchange of knowledge takes place between parents and their children and parents with their own parents.  4. DSM needs fit into family lives and is profoundly shaped by family life, sharing knowledge, care, and concerns. | |
| Hawkins,  2015 ^53^ | To explore the psychosocial factors that influence self-management and health care utilization in African American (AA) and Latino men with T2D. | Qualitative study, N=22 (Latino =12, AA =10) | Latino:  Mexico75%, Dominican Republic8.3%, El Salvador8.3%, Nicaragua8.3%,  Spanish; US, English;  <15y, n=2; ≥15y, n=10.  AA:  US, English. | | Latino:52,  AA:63 | | | 0 | T2D | | A CBPR framework | Focus groups interviews  (90min, 3 groups) | Inductive approach, deductive approach | | Five themes (a) social support as a motivator, (b) patient–provider relationships as facilitators of healthy behaviours, (c) immigration status and access to resources, (d) waiting until symptoms became severe before seeking medical attention, and (e) structural barriers. | |
| Jager,  2019 ^54^ | To explore the views regarding a healthy diet and dietetic care among ethnic minority T2D patients. | Qualitative study, N=12,  purposive sampling | Turkey50%,  Iraq25%,  Curacao, Netherlands Antilles8.3%,  Morocco16.7%,  Turkish, Arabm or Berber;  Netherlands,  Duch;  10-46y. | | 44-87 | | | 66.7 | T2D | | The Attitudes, Social influence, and self-Efficacy (ASE) model; Kleinman’s explanatory model of illness. | In-depth, semi-structured interviews | A deductive qualitative analysis | | All respondents acknowledged the importance of a healthy diet. What they considered healthy was determined by culturally influenced ideas about health benefits of specific foods. Hindrances for dietary change were lack of self-efficacy and social support. Social influences were experienced both as supportive and a hindrance. | |
| Jamil,  2022 ^55^ | To explore how cultural perspectives of South Asian immigrants impact medication adherence to for T2D and CVD. | Cross-sectional qualitative study, N=12 | Pakistan 58%, India33%, Bangladesh9%; English, Urdu, Hindi, or Bengali speaking;  US, English;  24 or (1-45). | | 63 or (49–75) | | | 50 | T2D | | Dimensions of Medication Adherence, the Patient Explanatory Model; the HOPE questions. | A semi-structured interview | Leininger's four phases of qualitative data analysis. | | Five themes: (a) Numerical results motivated health change; (b) Open communication improved medication adherence; (c) Self-management and autonomy valued; (d) Religious/spiritual beliefs may strengthen medication adherence; and (e) Complementary and alternative medicines (CAM) augment Western medicines. | |
| Jowsey,  2011 ^56^ | To explore the immigrant experience of T2D in terms of communication, self-management support and agency, and the implications for self-management programs. | Secondary analysis of the SCIPPS qualitative study,  N=32 (Immigrants=15, Australian-born=17),  purposeful sampling | 1.Immigrants (patients=10, carers=5):  Patients: Finland=1/Finnish, France=1/French, Spanish, Hungary=1/Hungarian, Hong Kong=1/Cantonese, Germany=1/German,Philippines=2/Tagalog,Turkey=2/Turkish,China=1/Cantonese,Mandarin.  2.Australian-born (patients=15, carer=2): Australia=17, English;  Australia, English. | | Immigrants:  51-85,  Australian-born:38-81 | | | Immigrants:60  Australian-born:  41.2 | T2D,  Immigrants=10, Australian-born=15 | | N/A | In-depth, semi-structured interviews, Survey | A qualitative content analysis,  descriptive analysis | | 1.Linguistic and cultural barriers.  2.Older people who were born overseas face obstacles to effective engagement.  3.The difference between the experiences of immigrants and Australian-born participants. | |
| Joo,  2016 ^57^ | To explore barriers to and facilitators of DSM among first-generation Korean immigrants with T2D. | Descriptive qualitative study,  N=18,  convenience and purposive sampling | Korea, Korean;  US, English;  25(5). | | 68.5(2.50) | | | 47.8 | T2D,  11-14y (35%), ≥15y (65%). | | N/A | Focus groups, a semi-structured, open-ended questions, individual interview. | Descriptive statistics, standard content-based analysis. | | 1. Barriers: the high cost of T2D care, language issues, loss of self-control, memory loss and limited access to healthcare resources.  2. Facilitators: time, seeking information, and family and peer supports.  3. Including family members, considering age-related limitations, and developing materials written in Korean may empower positive diabetes outcomes in this group. | |
| Kaltman, 2015 ^58^ | To examine the design and implementation of an integrated behavioural intervention that will simultaneously target T2D and depression self-management among the Latino immigrants. | Qualitative study,  N=21  (patients=14, family members=7) | Patients: El Salvador 50.0%, Honduras 21.4%, Guatemala 14.3%, Mexico14.3%. Family members: El Salvador 57.1%, Honduras14.3%, Guatemala28.6%;  US, English;  Patients: 14.36 (6.03), Family members: 14.0(8.51). | | Patients:  52.93 (9.83)  Family members: 44.0(17.24) | | | Patients: 79, Family members:71 | Patients:  T2D and depression, 7.29(4.39). | | N/A | Individual semi-structured interviews,  focus groups interviews | The stepwise process, based on consensual qualitative research | | Content:  1.Challenges with T2D management.  2.The role of depression in T2D patients.  3.Family involvement.  4.Perspective on design of future intervention.  5.Barriers/motivators to intervention participation.  6.Perspectives on family member involvement in the intervention. | |
| Kindarara, 2017 ^59^ | To describe Sub-Saharan African (SSA) immigrants' health-illness transition experiences associated with T2D self-management. | Descriptive qualitative study, N=10,  purposive and snowball sampling | Eritrea10%, Ethiopia10%, Kenya  20%, Liberia10%, Nigeria20%, Sierra Leone10%,  Zimbabwe20%,  African-languages;  US, English;  >10 y (70%). | | 60.3 or (44-76) | | | 50 | Self-reported T2D,  > 5y (60%). | | N/A | The demographic and transition experience questionnaire, face-to-face semi-structured in-depth interviews. | Qualitative content analysis | | Health-illness transition experiences:  (a) Knowledge of T2D self-management behaviours.  (b) Current T2D self-management behaviours.  (c) Inhibitors of T2D self-management.  (d) Facilitators of T2D self-management. | |
| Kokanovic, 2006 ^60^ | To elucidate the social meanings and interpretations that immigrant women with T2D, and the social support and professional advice that they received. | Qualitative study,  N=16,  purposive sampling | Greek25%, Chinese25%, Samoan /Tongan25%, Indians25%;  Australia, English. | | N/A | | | 100 | T2D,  >5y | | Arthur Kleinman’s explanatory model framework; A model for explaining health disparities. | In-depth interviews. | The thematic analysis | | Themes:  1.Interactions with doctors.  2.Social support.  3.Distress, worry and protection.  4.Support that helps.  5.Cultural diversity. | |
| Leake,  2003 ^61^ | To describe self-management by uninsured Filipino immigrants with T2D. | Focused ethnographic qualitative study,  N=11,  purposive  sampling | Philippine, Filipino;  US, English;  7 or (0.25-32). | | 62 or (53-76) | | | 81.8 | T2D,  5 or (0.17-22). | | The Concept of Health Literacy | Semi-structured interviews, open-ended-questions | Descriptive analysis, Roper and Shapira | | 1.Barriers: cultural, financial, Job problems, insurance, hunger.  2.Explanatory model: pathophysiology, diagnosis,  etiology.  3.Family: advice, family member, Filipino, motivation.  4.Self-management behaviours. | |
| Leung,  2014 ^62^ | To investigate why first-generation Chinese immigrants with diabetes have difficulty obtaining, processing, and understanding diabetes related information. | Qualitative study,  N=29,  purposive sampling | China, Mandarin 62% or Cantonese38%;  US, English;  15 or (6-39). | | 63.6(12.2) | | | N/A | T2D, 1-5y(34%),6-10y(34%),11-15y(14%),>15y(17%). | | N/A | Focus groups interviews (semi-structured, open-ended questions,90mins), individual interviews (60 mins). | A thematic content analysis | | Themes-affect different components of health literacy :  1.Cultural factors: high regard for authority; a desire to avoid being burdensome to others; a desire to be together or follow a collective approach.  2.Structural barriers: Insurance makes a difference; transportation issues; limited information in the Chinese-speaking community.  3. Personal barriers: unawareness of self-care responsibility; age related limitations. | |
| Magny-Normilus, 2020 ^63^ | To explore and describe the lived experience of adult Haitian immigrants managing T2D living in the US. | Phenomenological qualitative study,  N=16,  purposive sampling | Port-au-Prince37.6%, other 62.4%, Haitian Creole;  US, English; 11y. | | 55.63(6.5) or (40-63) | | | 75 | T2D | | N/A | A semi-structured face-to-face one-on-one interview(45mins) | Demographics, Moustakas’s existential data analysis. | | Four themes self-reliance (internal locus of control), spirituality (external locus of control), nostalgia for home, and a desire for positive patient-provider relationships (a lack of cultural sensitivity). | |
| Magny-Normilus, 2021 ^64^ | To describe the experiences of older adult Haitian immigrants in managing T2D. | Descriptive qualitative study,  N=20,  purposive sampling | Haiti origin, Haitian Creole ;  US, English;  >30y | | 69(7.3) | | | 60 | T2D,  12.6(7.0) | | N/A | Semi-structured, face-to-face in-depth audio-recorded individual interviews. | A modified iterative 7-step descriptive data analysis method, descriptive analyses. | | Themes related to experiences in managing T2D:  a) enduring financial hardship;  b) facing isolation outside the Haitian community (social isolation);  c) creating and maintaining good community relationships among themselves. | |
| McConatha,2020 ^65^ | To examine how aspects of culture, social support, isolation, and loneliness are perceived as influences in prediabetes or T2D management. | Qualitative study,  N=28 (T2D=13,  Prediabetes=15), convenience, snowball sampling | Middle-Eastern countries (Iran, Turkey, and Lebanon);  US, English;  >5y. | | 60-80 | | | 60.7:  T2D:  46.2,  Prediabetes: 73.3 | T2D/Pre  -diabetes, <3y. | | N/A | Semi-structured interviews. | The thematic analysis | | Challenges faced by diabetic and prediabetic patients:  (a) personal factors focusing on a loss of control, feelings of vulnerability, anxiety, and stress;  (b) interpersonal factors relating to relationship and social  support challenges;  (c) cultural factors relating to cultural disconnection, loneliness and resulting feelings of social isolation. | |
| Mitchell-Brown, 2017 ^66^ | To identify barriers and facilitators related to the diabetes education experience of Hmong Americans with T2D. | Qualitative study,  N=16, convenience sample | Laos, Hmong;  US, English;  >20y. | | 40-69 | | | 56.25 | Self-reported T2D,  1-16y. | | The  Framework for the Study of Access to Medical Care (FSAMC) | Focus groups  interviews (semi-structured) | An analytic method that combines inductive and deductive approaches | | The diabetes education experience: 1. The health care access category: 1) Consumer satisfaction (cost, quality); 2) The health care delivery system (resource, organization);3) Barrier to access of health care: mistrust of providers.  2. The health care experience category:1) Diabetes understanding. 2) Perceived barriers: language, self-management, stress.3) Perceived facilitators: focused culturally specific education, use of media, and peer support group. | |
| Mwalui, 2017 ^67^ | To understand the importance of culturally appropriate patient-provider communication to the T2D self-management among African immigrants; and offer  recommendations that could contribute to social change for this population. | Case  study,  N=15 (patients=10, CDEs=5), purposive sampling | Patients: Cameroonian=3, Ghanaian=1, Ugandan=1, Sierra Leone=1, Ethiopian=1, Nigerian=1, Gambia=1, Benin=1. CDEs:  Ghanaian=1, Ugandan=1, Afro-Caribbean=1, Kenyan=1, Nigerian=1;  US, English. | | Patients:  38-70,  CDEs:  32-51 | | | Patients: 70,  CDEs:  80 | Patients:  self-reported T2D,  2-5y. | | SCT | Focus groups interviews: open-ended questions. | Negative case analysis: a reflective approach | | 1.Themes generated from patients’ responses: (a)Support system. (b)Knowledge about behaviour toward DSM. (c) Lack of confidence in disease management due to language barrier. (d) Culture influences on DSM. (e) Ethnic diet and DSM. (f) Quality of life and diabetes. (g)Spirituality and diabetes. (h) Self-efficacy.  2.Themes generated from CDEs’ responses: (a)Using visuals to reinforce understanding. (b) Shared decisions. Lack of confidence. (c) Understanding patient circumstances. (d)Good health assessment. (e)Support groups/group activities. (f)Problem-solving skills. (g) Cultural component in patient care. (h) Patient cultural beliefs in disease management. | |
| Njeru,  2015 ^68^ | To develop a diabetes digital storytelling intervention with and for immigrant and refugee populations. | Phenomenological qualitative study, Step2: focus groups=37, Step3: digital stories development=8, purposive  sampling | Somalia or Latin America, Somali /Spanish;  US, English;  15.6 (8.2) | | Step2:  55.8(13.3),  Step3: 58.2 | | | Step2: 33.2,  Step3: 50 | Self-reported T2D,  Step2: 9.1(2.0),  Step3: 10.5y | | SCT; Narrative Theory; Social Construction Theory. | Focus groups interviews, a half day story development workshop. | Team-based thematic analysis | | Themes in Step2:  1.Diabetes diagnosis, understanding, and reactions.  2.Barriers to diabetes management.  3.Motivations and strategies for diabetes management.  4.Differences between Latino and Somali participants. | |
| Nam,  2013 ^69^ | To examine challenges in DSM among Korean Americans, and to guide clinicians in providing culturally appropriate diabetes care. | Qualitative study,  N=23 | Korea, Korean;  US, English;  25.3 (11.5) | | 58.5(7.3) or  (30-75) | | | 39.1 | T2D,  12.0(8.7). | | N/A | Focus groups (n=5 groups, 90-120min), open-ended questions | Descriptive statistics, content-based analysis | | Themes:  Q1 Social stigma and socialization.  Q2:1. Immigration life and DSM  (a)Priority and responsibility, (b)Language barriers  2.Diabetes-related knowledge and DSM. Q3: Family support in DSM. | |
| Patel,  2018 ^70^ | To understand SMBs of South Asian (SA) individuals with T2D. | Grounded Theory qualitative study, Study1= 14, Study2=17, Study3=7, Study4=30,  Study5=30,  Initial Purposive Sampling (Study1-3)/ theoretical sampling (Study2). | Study1: Health professionals: White British57.1%, SA42.95%.  Study2: Patients:  Indian 76.5%, Pakistani5.9%, African17.6%.  Study3:Family members:  Indian 85.7%, African 14.3%, Gujarati.  Study5: health professionals: White Caucasian 56.7%,  Ethnic Minority 43.3%.  US, English. | | Study1:  51.25 or (32-58), Study2:T2D patients: 54 or (35-73), Study3: 41 or (20-61).  Study4:30 SA with T2D  Study5:  18-64. | | | Study1:71.4,  Study2: 47.1,  Study3: 85.7,  Study 5: 76.7. | T2D,  Study2:  0.17-17y.  Study4:30 SA participants with T2D | | Social Norms  Theory;  SRM; HBM;  Communication Theory;  Social  Comparisons  Theory. | Study1: Semi-structured interviews (open-ended questions,28mins). Study 2: Semi-structured interviews.  Study3: Semi-structured interviews (face-to-face in-depth interviews,18min). Study5: Online questionnaires. | Study1-3:GT methodology and analysis. Study 4: Work-shop discussion with a structured conversation.  Study5: A Mann-Whitney analysis | | Themes: Study1:  (a) Health professional’s reflections on SA patients. (b) Delivery of diabetes care.  (c) Patient understanding and engagement with diabetes care.  Study2: Patient-centred care:  (a) Psychological distress and communication.  (b) Behavioural outcomes.  (c) Influencing variables take priority.  Study3. (a) Family roles. (b) Diabetes seriousness and acceptance.  Study 4: The GFT.  (a) Diabetes Communication. (b) Psychological Conflict.  (c) Cultural identity.  Study 5: GFT is a complex theoretical model and could also be used to develop interventions and resources for patients and/or family members. | |
| Peeters,  2015 ^71^ | To explore perspectives of Turkish migrants with T2D on adherence to oral hypoglycemic agents (OHA). | Grounded theory, qualitative study,  N=21,  theoretical sampling | Turkey, Turkish;  Belgium, Dutch;  1-20y,(47.6%),  >20y,(52.4%) | | 30-69 | | | 57.1 | T2D,  0–4y,23.8%;  5–9 y,42.9%;  10–14y,9.5%;  ≥15y,23.8%. | | N/A | In-depth interviews | A grounded theory approach analysis | | 1. Beliefs about diabetes.  2. Beliefs about OHA.  3. Patient–health care provider relationship.  4. Barriers of adherence to OHA.  5. Facilitators of OHA adherence. | |
| Pistulka, 2012 ^72^ | To examine the illness experience to inform future intervention for Korean immigrants living with T2D and hypertension. | Cross-cultural  qualitative descriptive inquiry study,  N=12 | Korea, Korean;  US, English | | 55.9 or (40-65) | | | 66.7 | T2D,8y | | N/A | In-depth interviews (n=12, 60-120min),  follow-up interviews (n=6,  20-60min) | The constant comparative method | | Themes:  1. Projecting an outward image of health in the public setting. 2. Motivations to maintain the outward image.  3.Strategies for maintaining an outward image of good health. | |
| Renfrew, 2013 ^73^ | To explore the potential barriers to care for Cambodian patients with diabetes. | Qualitative study, N=45 (patients=15, clinicians=25,  bilingual staff =5) | Cambodia, Khmer;  US, English. | | Patients:52 | | | Patients:60, Clinicians:70 | Patients: T2D | | N/A | Focus groups interviews (groups=5, 180mins): open ended questions | The thematic analysis (Strauss and Corbin’s three step approach) | | Themes: (a) Views of chronic disease, (b) Diabetes etiology and explanatory models; (c) Diabetes management (Medication, nutrition, exercise, use of remedies traditional in Khmer culture); (d) Communication (language and literacy, deference to physicians); (c) Psychosocial factors; and (f) Fears and challenges interacting with the health care system. | |
| Roth,  2022 ^74^ | To explore these experiences and perceptions and identify cultural aspects related to T2D self-management strategies among Sudanese migrants. | Qualitative study,  N=12, snowballing | Sudan, Arabic/ Indigenous Sudanese language;  Australia, English;  5-15y (83.3%), >15y (16.7%). | | 30-59 | | | 75 | Self-reported T2D | | N/A | In-depth semi-structured interviews (30 -75min) | The thematic analysis | | 1.Barriers to and facilitators of self-management of diabetes.  2.Themes of participants’ desire to maintain their cultural identity: traditional foods, traditional medicines, religion, collectivist culture, and choice of health professional. | |
| Weiler,  2009 ^75^ | To explore the socio-cultural influences and social context associated with living with T2D among migrant Latino adults. | Grounded theory techniques, qualitative descriptive study  N=10 | Mexico, Spanish;  US, English;  32 (3–65). | | | 56.5 or (46-55) | | 60 | T2D,  10.04 or  (1.5-40) | | N/A | In-depth semi-structured interviews | Descriptive statistics, constant comparative analysis | | Themes of self-management in a social environment:(a) family cohesion, (b) social stigma of disease, (c) social expectations/ perception of “Illness,” and (d) disease knowledge and understanding, was influenced by the social context. | |
| Wieland,2017 ^76^ | To inform the intervention as it related to four domains of DSM; to identify champion storytellers for the intervention development. | Qualitative study,  N=37,  purposeful sample | Somali 64.9%, Spanish 35.1%;  US, English;  15.6 (8.2) | | | 55.8 (13.3) | | 43.2 | Self-reported T2D,  9.1 (2.0) | | SCT | Focus groups | Team-based thematic analysis | | Themes:  1.Reactions to the diagnosis: relief or denial; shock, fear, hopelessness.  2. Barriers to diabetes management:  competing family needs; physical pain; lack of knowledge; food cravings and cultural customs; difficulties with changing habits; structural barriers.  3. Motivations and strategies for diabetes management: fear of  complications; intrinsic desire; family; faith; adapting to circumstances; self-discipline; acceptance; following  medical advice. | |
| Wang,  2012 ^77^ | To provide insights into the patients’ beliefs, experience, knowledge, skills in managing T2D, and support for a more authoritative  assessment of factors that differentiate Chinese Americans  with good control of their blood glucose from those with  poor control. | Focus group study, N=24  (Poorly controlled=7, Well-controlled=17) | China 41.7%, other 4.2%; Mandarin83.3%, Cantonese20.8%;  US, English  13.7 vs.16.6 | | | 56 vs.60.6 | | 57% vs.12% | Poorly controlled T2D (HbA1c>8), Well-controlled T2D (HbA1c<7) | | Determinants of effective self-management | Focus groups: open-ended  questions | Thematic analysis | | Common themes for both groups: interest in diet and Traditional Chinese Medicine.  Six themes:1. Find out their diabetes. Well-controlled: through routine testing; Poorly- controlled: after symptoms had developed.  2.Know about diabetes. The well-controlled group had a much deeper understanding of diabetes.  3. Cope with diabetes. Well-controlled people adjust their medication based on their level of activity; poorly controlled people simply stop taking the medication.  4. Self-manage their diabetes. Well-controlled people have specific goals and can state their reasons for choosing the methods of self-management. Poorly controlled people use symptoms as their motivation.  5. Factors that encouraged or hindered efforts to manage diabetes. Barriers: confusion with nutrition, a lack of Chinese language materials.  6. The relationship with the primary care provider and peer patients. Well-controlled: a positive relationship with a specific doctor. | |
| Washington, 2009 ^78^ | To explore self-care practices of Chinese American immigrants with T2D and identify risk factors related to lifestyle, attitudes, and health beliefs. | Qualitative study,  N=13 | China, Chinese;  US, English;  15 or (3-28) | | | 78 | | N/A | T2D,  9.2 or (1-31) | | CBPR;  The Vulnerable Populations Conceptual Model for Research  and Practice (VPCM) | Semi-structured interviews with open-ended questions, focus group interviews | Thematic analysis | | 1. Two themes reflecting self-care management:  (a) self-care practices which include diet, exercise, and medications.  (b) health beliefs which include attitudes, lifestyles, and health practices.  2. Although older adult Chinese Americans have been in the US for several decades, acculturation still has not occurred. | |

Notes: community-based participatory research (CBPR); cardiovascular disease (CVD); Certified Diabetes Educators (CDEs); diabetes self-management (DSM); Glycated Haemoglobin (HbA1c); social cognitive theory (SCT); type 2 diabetes (T2D); United States (US); not applicable (N/A).

| **Supplementary table 3-c: Characteristics of included mixed methods studies(N=18)** | | | | | | | | | | | | | |  |  |
| --- | --- | --- | --- | --- | --- | --- | --- | --- | --- | --- | --- | --- | --- | --- | --- |
| **Author,**  **year** | | **Study aims/focus** | **Sample size,**  **Sampling method** | **Country of origin/residence,**  **Language; Immigration duration**  ***Mean (SD) or Range (year)*** | **Sample**  **characteristics** | | | | **Theoretical framework** | | **Methodology, data collection, data analysis** | **Content of Self-management** | **Outcome variables** | **Summary of findings** | |
|  |  |  |  |  | **Age *Mean (SD) or Range (year)*, Sex, Ethnicity** | **Diagnosis, Disease Duration**  ***Mean (SD) or Range (year)*** | | |  | |  |  |  |  |  |
| Coffman, 2013 ^79^ | | To develop and pilot test nutrition and physical activity educational tools for immigrant Latina women who have T2D and low health literacy levels. | N=26, convenience sampling | Mexico62.9%, South America18.5%, Other18.5%,  Spanish;  US, English; 6.9(4.4). | 47(11.4),  Latinos,  Female  100%. | | | Self-reported T2D,  6.9(4.7) | Situated Learning Theory | | Pilot, explanatory sequential study;  Physical assessment, questionnaires, focus groups interviews;  Spearman’s rank correlation coefficient, a descriptive summary of each theme | Diabetes education program:  1.An educational class (2.5 hours):  1) Nutrition content. 2)Physical activity strategies: promote walking and quantify distances; goal setting; safe exercise education; use a Yamax Digi-Walker CW-600 pedometer.  2. A focus group to assess the program. | 1.Nutrition log.  2.Height, Weight, BMI.  3. Health literacy.  4. A log of steps taken.  5.Participants’ experience. | 1.The women reported that the pedometer and logs helped them increase vegetable consumption and physical activity.  2.The women were able to use the tools and logs even if they had low health literacy levels.  3. Barriers that prevented the women from walking and using the pedometer: inclement weather, illness, and physical limitations, including poor vision and dizziness. | |
| Islam,  2013 ^80^ | | To explore the impact and feasibility of a pilot CHW intervention designed to improve health behaviours and clinical measures among Korean Americans identified as prediabetes. | N=48(Intervention=21/25, Control=14/23) | Korea, Korean;  US, English | 59.7 (8.1), Female 64% | | | Prediabetes,  22.6 (10.3) | | N/A | Explanatory sequential with a RCT first;  Questionnaire, focus groups interviews;  Descriptive statistics, paired-sample t-tests, Chi square tests, Narrative analysis techniques. | A community-based CHW intervention (2-h group sessions, 3 weeks/session): consisted of 6 workshops held by CHWs on diabetes prevention, nutrition, physical activity, diabetes complications, stress and family support, and access to health care. | 1.Demographics.  2.Weight, BMI, WC, BP, glucose, and cholesterol.  3. Health behaviours: physical activity, nutrition, food behaviours, diabetes knowledge, self-efficacy, and mental health.  4.Health access: insurance and self-reported health.  5. Experiences in implementing the program. | 1. The CHW model is acceptable and appropriate to the target community and helps to promote positive directional changes in weight, WC, diastolic physical activity, nutrition, diabetes knowledge, and mental health.  2. Barriers:1) Difficulty accessing Korean immigrants due to busy work schedules.2) Misperception that having a regular doctor means do not need program.3) Misperception that if not diabetic or no symptoms, do not need program. 4)Lack of understanding regarding CHWs and their role in the program. 5)Difficulty retaining participants due to work schedules, travel to home country. | |
| Islam & Wyatt,  2013 ^81^ | | To explore the impact and feasibility of a pilot CHW intervention to improve diabetes management among Bangladeshi-American individuals with T2D. | N=26 | Bangladesh, Bengali;  US, English;  14.4 (7.8) | 53.4 (9.4) or (21-85),  Female  57.7%,  Bangladeshi | | | T2D | | CBPR  framework | Convergent parallel; Medical records, questionnaires, interview;  Fisher’s exact tests, Paired t-tests, Narrative analysis techniques | CHW-facilitated 2.5-hour group sessions (one session/month, 6sessions). It includes an overview of diabetes, nutrition, physical activity, diabetes complications, stress and family support, and access to health care. | 1.Demographics.  2.Diabetes knowledge.  3.Medication adherence.  4.Self-efficacy on nutrition and physical activity.  5.Mental health.  6.Experiences in implementing the program. | Quantitative findings:  The intervention improved the participants’ diabetes knowledge, exercise, and diet to control diabetes, frequency of checking feet, medication compliance, self-efficacy, physical activity, HbA1C, weight, and BMI.  Qualitative findings:  1. The intervention demonstrated high acceptability and suggested efficacy in improving diabetes management outcomes among Bangladeshi immigrants in an urban setting.  2. CHWs helped overcome barriers and facilitated program outcomes through communal concordance, trust, and leadership in the targeted population. | |
| Rankin,  1997 ^82^ | | To teste translated and back-translated instruments to ascertain factors related to diabetes management in a group of Chinese immigrants T2D. | N=30,  convenience sampling | Mainland China (77%), Mandarin/Taiwanese/Cantonese; US, English;  13(11). | 63.6 (7.8) or (46-80),  Female 43%, Chinese | | | T2D | | N/A | Cross-sectional, descriptive data from convergent parallel;  Questionnaires, Open-ended questions;  Reliability and validity analyses. | N/A | 1.Diabetes knowledge.  2.Social environment.  3. QoL issues related to diabetes.  4.Depression.  5.Satisfaction with family functioning.  6. Psychosocial needs. | 1. Participants had received limited diabetes education, reported many problems managing diabetes, and reported dissatisfaction with their QoL, especially regarding social and vocational issues.  2. The Chinese immigrants’ depression scores surpassed the US mean for depression clinical screening.  3. Diet and exercise were the highest rated of non-supportive diabetes-related family behaviours, while blood glucose testing, and medications were rated lower.  4. Most participants expressed fear regarding having to use insulin and believed that if they were not using insulin their diabetes was less severe. | |
| Jayne,  2001 ^83^ | | To demonstrate the application of Leventhal's Self-Regulation Model with a group of Chinese immigrants with T2D. | N=30,  convenience sampling | Mainland China (77%), Cantonese/Mandarin (88%);  US, English;  13y. | 46-80,  Female 43%,  Chinese | | | T2D | | Leventhal’s Theory of Self-Regulation | Ethnographic qualitative data after a cross-sectional study;  Individual interviews  (open-ended questions); Ethnographic qualitative analysis, content analysis. | Illness perceptions | Themes, patterns, and categories that exemplified the self-regulation model. | 1.Application of the Leventhal self-regulation model was useful in describing Chinese immigrants’ perceptions of diabetes.  2. The subcategories of illness representation: cause of illness, identity of the illness with symptoms, consequences of having the illness, and the illness timeline.  3.Coping strategies included wishful thinking, belief in powerful others, keeping diabetes a secret, and avoiding social situations. Participants lacked the ability to appraise the effects of their coping strategies. | |
| Kellow, 2020 ^84^ | | To develop and evaluate a pilot T2D group education program designed specifically for Chinese migrants living in Australia. | N=35 | Hong Kong 38%, China50%,  Malaysia12%, Vietnam 3%; Cantonese;  Australia,  English;  25.7 (10.8) | 69(9),  Female 65%,  Chinese | | | T2D,  10 (IQR = 2.8-20.5) | | Association of Diabetes Educators (AADE)7 Self-Care Behaviours framework | Quantitative data from an Explanatory sequential design with a pre- post-test first;  Questionnaires, self-rated scale, reflexive memoing;  The Shapiro-Wilk  Test, paired sample t tests, Mann–Whitney U tests. | The Chinese diabetes education intervention: healthy eating, being active, monitoring, taking medications, problem solving, healthy coping and reducing risks. 5 × 2-h classroom-like group education sessions, 10 weeks.4-month follow-up. | 1.WHR, WC.  2.HbA1c, lipid panel.  3.AADE7 self-care behaviours.  4.Diabetes distress and self-rated health status.  5.Participants’ experience of attending the program: (not reported). | This education program for Chinese Australians  successfully reduced WHR and WC in participants and increased their frequency of DSM behaviours while reducing diabetes distress. | |
| Kaltman, 2016 ^85^ | | To determine the feasibility, acceptability, and preliminary effectiveness of an integrated self-management intervention that simultaneously targets diabetes and depression self-management in a Latino immigrant community. | N=18, convenience sampling | El Salvador 61%, Mexico22%, Honduras 11.1%, and Bolivia 5.6%, Spanish;  US, English. | 49.7(8.8), Female 56%, Latinos | | Uncontrolled T2D and depression | | | N/A | Explanatory sequential with a pilot trial first;  Questionnaire,  a semi-structured interview;  Descriptive analyses, Paired t-tests, and the thematic analysis. | The integrated intervention:  pleasant activities, exercise, glucose monitoring/medication adherence, healthy eating, stress management/problem solving, and social support. 45 min/session, 1 session/week, 6 sessions, followed by 2 once-monthly booster sessions. Each session included an education component, behavioural activation, and motivational interviewing. | 1.HbA1c.  2.Depression.  3.DSM Behaviours.  4. Patient activation.  5.Self-efficacy.  6.The feasibility, suggestions, and appropriateness of the intervention. | 1.The intervention may have a positive impact on diabetes and depression-related outcomes.  2. Participants reported an overall favourable experience and acceptance of the intervention.  3. Depression improved significantly, and most self-management behaviours, diabetes-related self-efficacy, and patient activation increased in both the intent-to-treat and treated samples.  4.The only self-management behaviour that did not improve significantly was exercise. | |
| Marylyn, 2017 ^86^ | | To explore the potential effect of the intervention on the health–illness transition of Mexican immigrant women with T2D. | N=15 | Mexico, Spanish;  US, English;  21(12.35) or  (5–46) | 53 (11.05) or (36–72),  Female 100%, Hispanic | | T2D,  3(2.58) or  (0.5–10) | | | An emerging  middle-range theory | Convergent parallel;  Questionnaires, focus groups discussion(60min);  Descriptive statistics, Paired t tests, the Thematic analysis. | The community-based intervention(6-month): 1.A group component: a monthly 30-minute class (healthy eating, physical activity, problem solving, healthy coping, and reducing risks), followed by a 1-hour focused discussion, and ended with 10 mins physical activity.  2. An individual component: 60-90min/session, 4 sessions in total, including healthy eating, managing stress, and physical activity. | 1.Diabetes knowledge.  2. Social support.  3. Psychosocial problems.  4. Health-related behaviour problems.  5. Self-efficacy. | 1.Quantitative findings: There were significant changes between pre- and post-intervention in the psychosocial problems and health-related behaviour problems (e.g., nutrition, physical activity, healthcare supervision, medication regimen, and sleep and rest).  2. Qualitative findings: Themes: 1) Difficulty in acknowledging diabetes as part of my Life, 2) Still adjusting to change and difference, and 3) Putting family first. | |
| Pettersson,2023 ^87^ | | To describe self-care maintenance, possible changes, and factors related to unchanged self-care maintenance; to describe well- being, social support, and the need for support from healthcare services in migrant patients with T2D during the COVID-19. | N=79 | Middle East47%,  European countries 35%,  outside Europe17%;  Sweden, Swedish;  33 or (6-62). | 69 (11),  Female  49% | | T2D,  13 or  (1-40) | | | The middle-range theory | A triangulation design with cross-sectional data;  Questionnaires, open-ended questions; Student T-test, Pearson bivariate correlation, χ2, direct content analysis. | Changes in self-care maintenance: maintain an active lifestyle; perform physical exercise; eat a balanced diet; avoid eating salt and fats; limit alcohol intake; avoid getting sick; avoid cigarettes; take care of your feet; maintain good oral hygiene; keep appointments with health care; have health check-ups on time; take all prescribed medicines. | 1.Self-care maintenance for diabetes, and the participants’ experiences of possible changes in their self-care maintenance.  2.Self-efficacy.  3.Well- being. | 1.The majority (76%) had changed their self-care in some way during the COVID-19 pandemic, most often the self-care behaviours related to PA and diet.  2.Factors related to self-care maintenance: region of birth, well-being, male gender, and being married/living with somebody.  3.The relationship between well- being, social support, and need for support from healthcare services. | |
| Piombo, 2020 ^88^ | | To test if a culturally tailored intervention, based on customized diet and transcultural  mediator’s support, can improve diabetic immigrants’ food habits. | N=55 | Bangladesh67%,Morocco18%,Algeria4%,Tunisia 2%,Egypt 9%,Bangladeshi and Arabic;  Italy, Italian;  11.4 or (0–41). | 44.3 or (22–65), Female  11% | | T2D | | | N/A | Explanatory sequential with a pre-post first; Semi-structured questionnaires;  Paired Wilcoxon Signed-Rank test; a constant comparative method. | A culturally tailored dietary-plan: nutritional counselling and the choice of personalized diets according to the patient’s culture of origin. Baseline, 3-month, and 6-month follow-up. | 1.Demographics.  2.Food habits, food choice determinants, barriers in dietary management.  3. Customer satisfaction, adherence to diet and the recommended  activity levels. | 1.The worst dietary habits was generic inadequate food intake and excessive sugar consumption; Patients’ food habits improved at six-month follow-up, and their customer satisfaction was generally high.  2.Barriers and facilitators of dietary management.  3.No significant relationship between the ease of adapting to a healthier diet and the immigrants’ length of stay in Italy. | |
| Shultz, 2009 ^89^ | | To identify Slavic women’s beliefs about eating;  their dietary behaviours for health; and the influences on their dietary beliefs and behaviours. | N=10, convenience sampling | Ukraine 50%, Russia 20%, Kazakhstan 20%, Other 10%, Russian;  US, English;  8.6 (5.1). | Female 100% | | T2D,  9.2(5.4) | | | The Purnell Model for Cultural Competence | Qualitative data from a convergent parallel design;  Questionnaire, physical assessments, a semi-structured interview (29 open-ended items);  Descriptive statistics, content analysis. | Diet, medications, and use of medical care, as well as influences on diabetes beliefs and behaviours | 1.Dietary behaviours and beliefs.  2.Influences on dietary behaviours and beliefs. | Themes: 1. Dietary behaviours and beliefs:  1)Behaviour: giving up or reducing amounts of foods; eating more of certain foods; compensations.2) Beliefs about diet to manage diabetes; being healthy or maintaining a healthy heart.  2.Influences on dietary behaviours and beliefs: motivations; advice from health care providers; family/social; barriers; educational needs and experiences.  3.Barriers:1) Dietary control. 2) Barriers to cooking or eating the way I need to. 3) Inadequate finances. 4) Lack of fresh produce. 5) Habits difficult to break. 6) Lack of time difficult to think about diet all the time. 7) After advice to eat smaller portions more frequently, experienced weight gain.  4.Motivations:1) Want to be healthy. 3) Weight control.4) I don’t want to get worse. | |
| Smith-Miller, 2016 ^90^ | | To examine the relationship among knowledge, self-efficacy, health promoting behaviours, and T2D self-management among recent limited English proficient immigrants. | N=30,  snowball sampling | Mexico 83.3%,  other Latin American countries 16.7%,  Spanish;  US, English; <5y. | 27-86,  Female 63.3% | | T2D | | | Social cognitive theory | Quantitative data from a convergent parallel design; Demographics, questionnaire/scale, a semi-structured interview;  Descriptive statistics,  factor analysis; multiple linear regression  models; and structural  equation modelling with bootstrapping. | Individual’s knowledge, and self-efficacy | 1.Screening.  2. Socio-Demographics, height, weight, WC, BP, and HbA1c.  3. Diabetes’ knowledge.  4. Self-efficacy. | 1. The study population was on average not successfully managing their T2D and is unlikely to do so without linguistically and culturally appropriate interventions.  2. The participants adequately controlled their BP but most of the participants were overweight. Factor analysis supported the use of HbA1c as the sole measure of T2D management.  3.A low understanding of diabetes was found among the population and overall knowledge was found to be an important component of T2D self-management. | |
| Smith-Miller,  2017 ^91^ | | To explore T2D self-management experiences, behaviours, socio-cultural barriers, and facilitators, and examine the HbA1c levels among Hispanic immigrants. | N=30,  purposive and snowball sampling | Mexico 87%,  other Latin American country 13%, Spanish;  US, English; <5y. | 27-86,  Female 63% | | T2D,  >1y | | | Social cognitive theory | Qualitative data from a convergent parallel design; One-to-one semi-structured interviews, questionnaires;  Directed content analysis, constant comparative methods. | Medication, diet, physical activity, blood glucose monitoring, foot or eye care; health care providers; socio-economic influences. | 1.HbA1c.  2.Information seeking/knowledge acquisition.  3. Social environment.  4. Self-management behaviours.  5.Self-management practices. | 1.Information-seeking/knowledge.  2.The most difficult self-management behaviours: adhering to recommended dietary practices and engaging in regular physical activity.  3. Barriers to effective T2D self-management.  4.Social environment: family influences/vicarious learning.  5.Living with diabetes. | |
| Shah,  2022 ^92^ | | To develop and test the feasibility of a tailored group visit model for Bangladeshis with T2D or prediabetes based in primary care. | Focus group=50, Pre-post study=14 | Bengal, Bengali;  US, English | Focus group:  45.2, 46%  Pre-post study:  50 or  (23-74),  57% | | Self-reported T2D or Prediabetes | | | N/A | Convergent parallel with a single-arm feasibility study;  Focus group discussions, physical assessments;  Thematic analysis, descriptive analyses, one tailed paired t tests | A culturally acceptable intervention: 16-week program focusing on healthy diet, exercise, and weight loss. | 1.Factors influencing lifestyle behaviours.  2.Feasibility and participant satisfaction. 3.Weight, BP, cholesterol, and HbA1c. | 1.Themes from the formative focus groups were closely tied to sociocultural beliefs and included:  dietary patterns, physical activity perceptions, and healthcare access concerns.  2. Participant attendance in the feasibility study was 50%, and the levels of participants’ satisfaction were high. Statistically significant reductions in mean weight, BP, and triglycerides were noted.  3. This lifestyle program based in primary care is feasible and acceptable for Bangladeshi immigrants. | |
| Tang,  2015 ^93^ | | To examine the feasibility and potential health impact of a DSM education and support intervention involving peer support on glycemic control and diabetes distress. | N=32/41 | India78%, other(Uganda, Tanzania, Pakistan, Kenya)22%; Punjabi;  Canada, English;  29(14) | 67(9) or (43-89),  Female  73% | | T2D,  13(9) | | | N/A | Explanatory sequential with a pre-post first;  Self-report survey, physical assessments, open-ended questions;  Spearman’s correlation coefficient, negative binomial regression model, paired t-tests, content analysis | DSM intervention: 1) Education sessions (6 weeks): participants’ self-management questions, goal setting and action planning. 2)Support sessions (18 weeks): self-management challenges, shared emotions, asked self-management questions, problem-solved in a group, set goals, and developed and evaluated action plans. | 1.Feasibility.  2.HbA1c, lipid profile, BP, BMI, WC.  3. Diabetes distress.  4.Demographic.  5.Programme satisfaction. | 1. The average attendance rate for the total 24-week intervention is 65%. This intervention may have a positive impact on diabetes distress, but not on HbA1c levels.  2. Themes of program satisfaction (a) the suitability of faith-based locations for lifestyle change interventions; (b) peer leaders’ level of diabetes knowledge; (c) teaching methods and learning styles for participants with low literacy levels; (d)the intensity of diabetes education; (e) the match between professional expertise and diabetes education delivered;  (f) the addition of active physical activity. | |
| Venkatesh,  2013 ^94^ | | To examine the association between the degree of acculturation and HbA1c in Asian Indians adults with T2D. | N=30,  convenience sampling | Asian, India;  US, English. | Female 46.7% | | T2D | | | N/A | Convergent parallel;  Questionnaire, interview (open-ended questions);  A two-step multiple linear regression analysis, content analysis. | Self-management behaviours (ADA): healthy eating and weight management, regular exercise, SMBG, foot care, regular consultation with a physician, and compliance with prescribed medications. | 1.Acculturation.  2.Demographics.  3. HbA1c.  4. Lifestyle changes after immigration and perceptions about diabetes management in the US. | The two themes relative to acculturation and glycemic control status:  1.Changes in lifestyle after immigration.  2. Ease of management of diabetes in the US. | |
| Vang,  2021 ^95^ | | To provide diabetes education in Hmong language to improve diabetes knowledge, QoL for the Hmong population with T2D; to seek their feedback. | Quantitative=7, Qualitative=6 | Asia, Hmong language;  US, English | N/A | | T2D | | | The theories of Leininger | Explanatory sequential;  A telephone  questionnaire with open-ended questions;  A Two-Tailed Wilcoxon Signed Rank Test, content analysis. | The Hmong DSM and support program: three 50-min educational meetings, which were conducted two weeks apart for a total of six weeks. | 1.Diabetes knowledge.  2. QoL.  3.Participants’ feedback. | After participating in the program, the participants’ diabetic knowledge improved, and they had fewer unhealthy days than before program implementation. | |
| Yeh,  2023 ^96^ | | To describe the formative research and process of developing a culturally tailored web-based DPP intervention for Chinese Americans with prediabetes. | N=24 | China, Chinese;  US, English | >18y | | Pre-diabetes | | | The health planning logic of the PRECEDE-PROCEED Model (PPM) | Formative research using triangulation design;  Focus groups (n=3);  A thematic analysis | The development of an online DPP curriculum through (1) a  literature review, (2) three focus groups, and (3) a community advisory board meeting. | 1.Overall reaction to the content of study materials  (Images, colour, fonts, and translations of the curriculum).  2. Perceptions and experiences  with web-based interventions.  3. Strategies for maintaining a healthy lifestyle. | 1. Key themes:  1) Barriers to behavioural changes.  2) Feedback on curriculum content and suggestions.  3) Web-based intervention acceptability.  4) Web-based intervention feasibility.  5) Web-based intervention implementation and modifications.  2.Web-based intervention acceptability and feasibility: well-received, useful and easily accessible.  3.The acceptability for web-based self-monitoring tools was very high among Chinese Americans; they thought the pedometer helped increase their physical activity. | |

Note: American Diabetes Association (ADA);Body Mass Index (BMI); blood pressure (BP); community health worker (CHW); diabetes self-management, DSM; Waist Hip Rate (WHR) ;Waist Circumference (WC); Quality of Life (QoL); Glycated Haemoglobin (HbA1c); Uncontrolled T2D (HbA1c ≥8 mg/dL); diabetes prevention program(DPP).

Reference:

1. Alzubaidi H, Mc Mamara K, Chapman C, Stevenson V, Marriott J. Medicine-taking experiences and associated factors: comparison between Arabic-speaking and Caucasian English-speaking patients with type 2 diabetes. Diabet Med. 2015;32(12):1625–33.

2. Alzubaidi H, Sulieman H, Mc Namara K, Samorinha C, Browning C. The relationship between diabetes distress, medication taking, glycaemic control and self-management. Int J Clin Pharm. 2022;44(1):127–37.

3. Amirehsani KA. Self-care expressions, patterns, and practices of Latinos/Hispanics for the management of type 2 diabetes [internet]. 2011:P231 Available from: https://libres.uncg.edu/ir/uncg/f/Amirehsani_uncg_0154D_10737.pdf

4. Chesla CA, Chun KM, Kwan CM, Mullan J, Waters C. Testing a culturally adapted behavioral diabetes intervention with Chinese immigrants. Diabetes. 2013;61: A196.

5.Chesla CA, Kwan CM, Chun KM, Stryker L. Gender differences in factors related to diabetes management in Chinese American immigrants. West J Nurs Res. 2014;36(9):1074–90.

6. Choi SE, Rush EB. Effect of a short-duration, culturally tailored, community-based diabetes self-management intervention for Korean immigrants: a pilot study. Diabetes Educ. 2012;38(3):377–85.

7. Choi SE. Diet-Specific family support and glucose control among Korean immigrants with type 2 diabetes. Diabetes Educ. 2009;35(6):978–85.

8. Choi S, Toyama J, Brecht M-L. Effect of disclosure on receiving spousal support in Korean immigrants with type 2 diabetes. Diabetes Educ. 2020;46(6):559–68.

9. Coffman MJ, Norton CK, Beene L. Diabetes symptoms, health literacy, and health care use in adult Latinos with diabetes risk factors. J Cult Divers. 2012;19(1):4–9.

10. Eh K, McGill M, Wong J, Krass I. Cultural influences on self-management of type 2 diabetes (T2DM) among Chinese immigrants in Australia. Int J Pharm Pract. 2016; 24:11.

11. Hempler NF, Fagt C, Olesen K, Wagner S, Rasmussen LB, Laursen DH, et al. Improving health and diabetes self-management in immigrants with type 2 diabetes through a co-created diabetes self-management education and support intervention. J Community

Health. 2022;48(10:141-151.

12. Ho EY, Pak S, Leung G, Xu SW, Yu CK, Hecht FM, et al. Pilot Cluster Randomized Controlled Trial of Integrative Nutritional Counseling Versus Standard Diabetes Self-Management Education for Chinese Americans with Type 2 Diabetes. Health Equity.

2020;4(1):410–20.

13. Hu L, Trinh-Shevrin C, Islam N, Wu B, Cao SM, Freeman J, et al. Mobile Device Ownership, Current Use, and Interest in Mobile Health Interventions Among Low-Income Older Chinese Immigrants With Type 2 Diabetes: Cross-sectional Survey Study.

JMIR Aging. 2022;5(1).

14. Hu L, Islam N, Trinh-Shevrin C, Wu B, Feldman N, Tamura K, et al. A Social Media-Based Diabetes Intervention for Low-Income Mandarin-Speaking Chinese Immigrants in the United States: Feasibility Study. JMIR Formative Research. 2022;6(5).

15. Huang YC, Zuniga J, Garcia A. Illness perceptions as a mediator between emotional distress and management self-efficacy among Chinese Americans with type 2 diabetes. Ethnicity & health. 2022;27(3):672–86.

16.Hyman I, Shakya Y, Jembere N, Gucciardi E, VissandjÃ©e B. Provider- and patient-related determinants of diabetes self-management among recent immigrants: Implications for systemic change. Can Fam Physician. 2017;63(2): e137–44.

17. Hyman I, Gucciardi E, Patychuk D, Rummens JA, Shakya Y, Kljujic D, et al. Self-management, health service use and information seeking for diabetes care among black caribbean immigrants in Toronto. Can J Diabetes. 2014;38(1):32–7.

18. Hyman I, Patychuk D, Zaidi Q, Kljujic D, et al. Self-management, health service use and information seeking for diabetes care among recent immigrants in Toronto. Chronic Dis Inj Can. 2012;33(1):12–8.

19. Iten A, Jacobs E, Lahiff M, FernÃ¡ndez A. Undocumented immigration status and diabetes care among Mexican immigrants in two immigration “sanctuary” areas. J Immigr Minor Health.. 2014;16(2):229–38.

20. Jordan DN, Jordan JL. Self-care behaviors of Filipino-American adults with type 2 diabetes mellitus. J Diabetes Complications. 2010;24(4):250–8.

21. Jordan DN, Jordan JL. Foot self-care practices among Filipino American women with type 2 diabetes mellitus. Diabetes Ther. 2011;2(1):1–8.

22. Kim MT, Han H-R, Song H-J, Lee J-E, Kim J, Ryu JP, et al. A community-based, culturally tailored behavioral intervention for Korean Americans with type 2 diabetes. Diabetes Educ. 2009;35(6):986–94.

23. Kim MT, Kim KB, Huh B, Nguyen T, Han HR, Bone LR, et al. The effect of a community-based self-help intervention Korean Americans with type 2 diabetes. Am J Prev Med. 2015;49(5):726–37.

24. Kim MT, Kim KB, Ko J, Murry N, Xie B, Radhakrishnan K, et al. Health literacy and outcomes of a community-based self-help intervention: a case of Korean Americans with type 2 diabetes. Nursing Research. 2020;69(3):210–8.

25. Krieg LT. APRN-Led Culturally Tailored Diabetes Self-Management Education (DSME) for Spanish-Speaking Hispanic Americans (SSHAs) [PhD Thesis]. University of Virginia. 2017;05.

26. Loya JC. Salud Paso por Paso: A culturally-tailored physical activity intervention with Hispanic adults with type 2 diabetes mellitus [PhD Thesis]. University of Missouri-Columbia. 2021;06.

27. Mier N, Smith ML, Carrillo-Zuniga G, Wang X, Garza N, Ory MG. Personal and cultural influences on diabetes self-care behaviors among older Hispanics born in the U.S. and Mexico. J Immigr Minor Health. 2012;14(6):1052–62.

28. Naccashian Z. The impact of diabetes self-management education on glucose management and empowerment in ethnic Armenians with type 2 diabetes. Diabetes Educ. 2014;40(5):638–47.

29. Pettersson S, Hadziabdic E, marklund H, Hjelm K. Lower knowledge about diabetes among foreign‐born compared to Swedish‐born persons with diabetes - a descriptive study. Nurs Open. 2018;6(2):367-376.

30. Park SW. Health Literacy, Illness Perception, and Diabetes Self-management in Korean-speaking Immigrants with Diabetes [Internet]. 2020: p105. Available from: <https://rc.library.uta.edu/uta-ir/bitstream/handle/10106/29637/PARK-DISSERTATION->

2020.pdf?sequence=1&isAllowed=y

31. Rechenberg K, Szalacha L, Martinez G, Graham M, Stauber L, Menon U. Feasibility and acceptability of a language concordant health coaching intervention delivered by nurses for Latinx with type 2 diabetes. Worldviews Evid Based Nurs. 2021;18(3):210–6.

32. Thabit H, Shah S, Nash M, Brema I, Nolan JJ, Martin G. Globalization, immigration and diabetes self-management: An empirical study amongst immigrants with type 2 diabetes mellitus in Ireland. QJM. 2009;102(10):713–20.

33. Vaccaro JA, Exebio JC, Zarini GG, Huffman FG. The role of family/friend social support in diabetes self-management for minorities with type 2 diabetes. J Nutr Health, 2014:2 (1): 1-9.

34. Wang CY & Chan SMA. Culturally tailored diabetes education program for Chinese Americans: a pilot study. Nurs Res. 2005;54(5):347-53.

35. Williams DA. The Influence of Psychosocial Adjustment on Medication Adherence among Uninsured Hispanic Immigrants Aged 40 to 64 Years-Old with a Type-2 Diabetes Diagnosis [Internet]. 2016:p141. Available from:

http://jbox.gmu.edu/bitstream/handle/1920/10511/Williams_gmu_0883E_11093.pdf

36. Abuelmagd W, Osman BB, Hakonsen H, Jenum AK, Toverud EL. Experiences of Kurdish immigrants with the management of type 2 diabetes: a qualitative study from Norway. Scand J Prim Health Care. 2019;37(3):345–52.

37. Alzubaidi H, Mc Narmara K, Kilmartin GM, Kilmartin JF, Marriott J. The relationships between illness and treatment perceptions with adherence to diabetes self-care: A comparison between Arabic-speaking migrants and Caucasian English-speaking patients.

Diabetes Res Clin Pract. 2015;110(2):208–17.

38. Alzubaidi H, Mc Namara K, Browning C. Time to question diabetes self-management support for Arabic-speaking migrants: exploring a new model of care. Diabet Med.2017;34(3):348–55.

39. Baghikar S, Benitez A, Patricia Fernandez P, Gao Y, Baig AA. Factors Impacting Adherence to Diabetes Medication Among Urban, Low Income Mexican-Americans with Diabetes. J Immigr Minor Health. 2019;21(6):1334–41.

40. Brunk DR, Taylor AG, Clark ML, Williams IC, Cox DJ. A culturally appropriate self-management program for Hispanic adults with type 2 diabetes and low health literacy skills. J Transcult Nurs.2017:28(2):187-194

41. Barbara S, Krass I. Self management of type 2 diabetes by Maltese immigrants in Australia: Can community pharmacies play a supporting role? Int J Pharm Pract. 2013;21(5):305–13.

42. Barko R, Corbett CF, Allen CB, Shultz JA. Perceptions of diabetes symptoms and self-management strategies: A cross-cultural comparison. J Transcult Nurs. 2011, 22(3):274–81.

43. Carolan-Olah M, Cassar A. The experiences of older Italian migrants with type 2 diabetes: A Qualitative Study. J Transcult Nurs. 2018;29(2):172–9.

44. Cha E, Yang K, Lee J, Min J, Kim KH, Dunbar SB, et al. Understanding cultural issues in the diabetes self-management behaviors of Korean immigrants. Diabetes Educ. 2012;38(6):835–44.

45. Choi SE, Lee JJ, Park JJ, Sarkisian CA. Spousal support in diabetes self-management among Korean immigrant older adults. Res Gerontol Nurs. 2015;8(2):94–104.

46. Chun KM, Chesla CA, Kwan CM. So We Adapt Step by Step: Acculturation experiences affecting diabetes management and perceived health for Chinese American immigrants. Social Science & Medicine. 2011;72(2):256–64.

47. Chesla CA, Chun KM, Kwan CML. Cultural and family challenges to managing type 2 diabetes in immigrant Chinese Americans. Diabetes Care. 2009;32(10):1812-6.

48. Chun KM & Chesla CA. Cultural issues in disease management for Chinese Americans with type 2 diabetes. Psychol Health. 2004;19(6):767-85.

49. Deng FY, Chan CB. Defining modifiable barriers to uptake of dietary recommendations in Chinese immigrants with type 2 diabetes: a qualitative study. FACETS. 2019; 4:551–65.

50. Fleming E, Carter B, Pettigrew J. The influence of culture on diabetes self-management: perspectives of Gujarati Muslim men who reside in northwest England. Journal of Nursing & Healthcare of Chronic Illnesses. 2008;17(1):51–9.

51. Fagerli RA, Lien ME, Wandel M. Experience of dietary advice among Pakistani-born persons with type 2 diabetes in Oslo. Appetite. 2005; 45 (3), 295–304.

52. Guell C. Diabetes management as a Turkish family affair: Chronic illness as a social experience. Ann Hum Biol. 2011;38(4):438–44.

53. Hawkins J, Watkins DC, Kieffer E, Spencer M, Espitia N, Anderson M. Psychosocial Factors That Influence Health Care Use and Self-Management for African American and Latino Men with Type 2 Diabetes. J Mens Stud. 2015;23(2):161–76.

54. Jager MJ, van der Sande R, Essink-Bot M-L, van den Muijsenbergh METC. Views and experiences of ethnic minority diabetes patients on dietetic care in the Netherlands - a qualitative study. Eur J Public Health. 2019;29(2):208–13.

55. Jamil A, Jonkman LJ, Miller M, Jennings L, Connor SE. Medication adherence and health beliefs among South Asian immigrants with diabetes in the United States: A qualitative study. JACCP J Am Coll Clin Pharm. 2022;5(8):829–36.

56. Jowsey T, Gillespie J, Aspin C. Effective communication is crucial to self-management: The experiences of immigrants to Australia living with diabetes. Chronic Illn. 2011;7(1):6–19.

57. Joo JY, Lee H. Barriers to and facilitators of diabetes self-management with elderly Korean-American immigrants. Int Nurs Rev. 2016;63(2):277–84.

58. Kaltman S, Talisman N, Serrano A, Cabassa LJ, Magee MF, Pulgar-Vidal O, et al. Type 2 Diabetes and Depression: Patient, Family Member, and Primary Care Provider Perspectives on the Development of an Integrated Self-Management Intervention. Diabetes

Educ. 2015;41(6):763–72.

59. Kindarara DM, McEwen MM, Crist JD, Loescher LJ. Health-Illness Transition Experiences With Type 2 Diabetes Self-management of Sub-Saharan African Immigrants in the United States. Diabetes Educ. 2017;43(5):506–18.

60. Kokanovic R, Manderson L. Social support and self-management of type 2 diabetes among immigrant Australian women. Chronic Illn. 2006;2(4):291–301.

61. Leake AR. Self-management by uninsured Filipino immigrants with type 2 diabetes [Internet]. 2003: P274. Available from: hhttps://scholarspace.manoa.hawaii.edu/server/api/core/bitstreams/b2f110b9-8308-457b-a1ad-761f4bdd8cf7/content

62. Leung AYM, Ai B, Hsin-Yi H, Song Song W, Chi I. Health literacy issues in the care of Chinese American immigrants with diabetes: a qualitative study. BMJ Open. 2014;4(11).

63. Magny-Normilus C, Mawn B, Dalton J. Self-Management of Type 2 Diabetes in Adult Haitian Immigrants: A Qualitative Study. J Transcult Nurs. 2020;31(1):51–8.

64. Magny-Normilus C, Whittemore R, Wexler DJ, Schnipper JL, Nunez-Smith M, Fu MR. Barriers to Type 2 Diabetes Management Among Older Adult Haitian Immigrants. The science of diabetes self-management and care. 2021;47(5):382–90.

65. McConatha JT, Kumar VK, Raymond E, Akwarandu A. Cultural dimensions of diabetes management: a qualitative study of Middle Eastern immigrants in the USJ Cross Cult Gerontol. 2020;35(1):85–98.

66. Mitchell-Brown F, Nemeth L, Cartmell K, Newman S, Goto K. A Study of Hmong Immigrants Experience with Diabetes Education: A Community-Engaged Qualitative Study. J Transcult Nurs. 2017;28(6):540–9.

67. Mwalui A. Understanding the Importance of Culturally Appropriate Patient-Provider Communication in Diabetes Self-Management [Internet]. 2017. p159. Available from:

https://scholarworks.waldenu.edu/cgi/viewcontent.cgi?article=4813&context=dissertations

68. Njeru JW, Patten CA, Hanza MMK, Brockman TA, Ridgeway JL, Weis JA, et al. Stories for change: Development of a diabetes digital storytelling intervention for refugees and immigrants to Minnesota using qualitative methods Health behaviour, health

promotion and society. BMC public health. 2015;15(1).

69. Nam S, Song HJ, Park SY, Song YS. Challenges of Diabetes Management in Immigrant Korean Americans. Diabetes Educ, 2013;39(2):213-21.

70. Patel T. The Development of a New Theoretical Framework to Explore Type 2 Diabetes Self-Management Behaviours in Uk South Asian Patients: Recommendations for Healthcare Practice [Internet]. 2018:p398. Available from:

http://researchonline.ljmu.ac.uk/id/eprint/10567/

71. Peeters B, Van Tongelen I, Duran Z, YÃ¼ksel G, Mehuys E, Willems S, et al. Understanding medication adherence among patients of Turkish descent with type 2 diabetes: a qualitative study. Ethnicity & health. 2015;20(1):87–105.

72. Pistulka GM, Winch PJ, Park H, Han HR, Kim MT. Maintaining an outward image: a Korean immigrant’s life with type 2 diabetes mellitus and hypertension. Qual Health Res. 2012;22(6):825–34.

73. Renfrew MR, Taing E, Cohen MJ, Betancourt JR, Pasinski R, Green AR. Barriers to care for cambodian patients with diabetes: results from a qualitative study. J Health Care Poor Underserved. 2013;24(2):633–55.

74. Roth P, Tang CY, Rumbold B, Gupta S. Knowledge and perceptions around self-management of type 2 diabetes among a Sudanese community in Australia: A qualitative study. Health Promot J Austr. 2022;33(3):869–79.

75. Weiler DM, Crist JD. Diabetes self-management in a latino social environment. Diabetes Educ. 2009;35(2):285–92.

76. Wieland, M. L., Njeru, J. W., Hanza, M. M., Boehm, D. H., Singh, D., Yawn, B. P., Patten, C. A., Clark, M. M., Weis, J. A., Osman, A., Goodson, M., Porraz Capetillo, M. D., Hared, A., Hasley, R., Guzman-Corrales, L., Sandler, R., Hernandez, V., Novotny, P.

J., Sloan, J. A., & Sia, I. G. (2017). Pilot Feasibility Study of a Digital Storytelling Intervention for Immigrant and Refugee Adults With Diabetes. Diabetes Educ, 43(4), 349-359.

77. Wang Y, Chuang L, Bateman WB. Focus group study assessing self-management skills of Chinese Americans with type 2 diabetes mellitus. J Immigr Minor Health. 2012;14(5):869–74.

78. Washington G, Wang-Letzkus MF, Washington G, Wang-Letzkus MF. Self-care practices, health beliefs, and attitudes of older diabetic Chinese Americans. Journal of Health & Human Services Administration. 2009;32(3):305–23.

79. Coffman MJ, Ferguson BL, Steinman L, Talbot LA, Dunbar-Jacob J. A health education pilot for Latina women with diabetes. Clinical Nursing Research. 2013;22(1):70–81.

80. Islam NS, Zanowiak JM, Wyatt LC, Chun K, Lee L, Kwon SC, Trinh-Shevrin C. A randomized-controlled, pilot intervention on diabetes prevention and healthy lifestyles in the New York City Korean community. J Community Health. 2013;38(6):1030-41.

81. Islam NS, Wyatt LC, Patel SD, Shapiro E, Tandon SD, Mukherji BR, et al. Evaluation of a community health worker pilot intervention to improve diabetes management in Bangladeshi immigrants with type 2 diabetes in New York City. Diabetes Educ.

2013;39(4):478–93.

82. Rankin SH, Galbraith ME, Huang P. Quality of life and-social environment as reported by Chinese immigrants with non-insulin-dependent diabetes mellitus. Diabetes Educ. 1997: 23(2):171-177.

83. Jayne RL, Rankin SH. Application of Leventhal’s self-regulation model to Chinese immigrants with type 2 diabetes. Journal of nursing scholarship: an official publication of Sigma Theta Tau International Honor Society of Nursing. 2001;33(1):53–9.

84. Kellow NJ, Palermo C, Choi TS. Not Scared of Sugarâ¢: Outcomes of a structured type 2 diabetes group education program for Chinese Australians. Health & social care in the community. 2020;28(6):2273–81.

85. Kaltman S, Serrano A, Talisman N, Magee MF, Cabassa LJ, Pulgar-Vidal O, et al. Type 2 Diabetes and Depression: A Pilot Trial of an Integrated Self-management Intervention for Latino Immigrants. Diabetes Educ. 2016;42(1):87–95.

86. Marylyn Morris M, Baird M, Pasvogel A, Gallegos G. Health-Illness transition experiences among Mexican immigrant women with diabetes. Fam Community Health. 2007;30(3):201–12.

87. Pettersson S, Jaarsma T, HedgÃ¤rd K, Klompstra L. Self-care in migrants with type 2 diabetes, during the COVID-19 pandemic. J Nurs Scholarsh.2023;55(1):167–77.

88. Piombo L, Nicolella G, Barbarossa G, Tubili C, Pandolfo MM, Castaldo M, et al. Outcomes of Culturally Tailored Dietary Intervention in the North African and Bangladeshi Diabetic Patients in Italy. Int J Environ Res Public Health.2020;17(23).

89. Shultz JA, Corbett CF, Allen CB. Slavic women’s understanding of diabetes dietary self-management and reported dietary behaviors. J Immigr Minor Health. 2009;11(5):400–5.

90. Smith-Miller CA, Berry DC, DeWalt D, Miller CT. Type 2 Diabetes Self-management Among Spanish-Speaking Hispanic Immigrants. J Immigr Minor Health. 2016;18(6):1392–403.

91. Smith-Miller CA, Berry DC, Miller CT. Diabetes affects everything: Type 2 diabetes self-management among Spanish-speaking Hispanic immigrants. Research in nursing & health. 2017;40(6):541–54.

92. Shah KM, Naing S, Kurra N, Weber MB, Islam N, Ali MK, Narayan KMV. A culturally adapted, social support-based, diabetes group visit model for Bangladeshi adults in the USA: a feasibility study. Pilot and Feasibility Stud. 2022;8(1):18.

93. Tang TS, Sohal PS, Garg AK. Rethinking peer support for diabetes in Vancouver’s South-Asian community: a feasibility study. Diabet Med. 2015;32(8):1077–84.

94. Venkatesh S, Weatherspoon LJ, Kaplowitz SA, Song WO. Acculturation and glycemic control of Asian Indian adults with type 2 diabetes. Journal of community health. 2013;38(1):78–85.

95. Vang K. Bridging the gap in knowledge deficit in type 2 diabetes among Hmong Americans [PhD Thesis]. Lenoir Rhyne University.2021:08.

96. Yeh MC, Lau W, Gong Z, Horlyck-Romanovsky M, Tung HJ, Zhu L, et al. Development of a web-based diabetes prevention program (DPP) for Chinese Americans: a formative evaluation approach. Int J Environ Res Public Health. 2023;20(1):599-612.
