# Supplementary material for: Cultural Influences, Experiences and Interventions Targeting Self‐Management Behaviours for Prediabetes or Type 2 Diabetes in First‐Generation Immigrants: A Scoping Review
Source: J Adv Nurs. 2024 Nov 21;81(6):2929–45. doi: 10.1111/jan.16621 (PMC12080094; doi:10.1111/jan.16621)
Supplement: Supplementary file 5 — Table S4. [file JAN-81-2929-s007.docx]

**Supplementary Table 4. Barriers to and facilitators of effective self-management**

| **Barriers** | **Theme** | **Examples of barriers identified** | **Citation** | **Facilitators** | **Theme** | **Examples of facilitators identified** | | **Citation** |
| --- | --- | --- | --- | --- | --- | --- | --- | --- |
| **Personal barriers** | Limited health  literacy | - Poor written literacy or diabetes literacy - Knowledge gaps and misinformation about T2D. - Unawareness of self-care responsibility | (Barbara & Krass, 2013; Kaltman et al., 2015; Kindarara et al., 2017; Leung et al., 2014; Mwalui, 2017; Njeru et al., 2015; Smith-Miller et al., 2017) | **Daily routine** | Seeking information | - Easy access to health information - Acceptance of the disease - Finding it easier - Learning more about diabetes | | (Joo & Lee, 2016; Kindarara et al., 2017; Njeru et al., 2015; Shah et al., 2022) |
|  | Failing to medication adherence and  self-monitoring | - Unwanted medication effects - Utility of medication - Desire to control diabetes with lifestyle instead of medication. - The use of herbal or other culturally based remedies - Memory loss due to T2D: forgetting to take medication and check blood glucose levels. | (Barbara & Krass, 2013; Joo & Lee, 2016; Mitchell-Brown et al., 2017; Renfrew et al., 2013) |  | Diabetes medication in their daily life | - Following medical advice - Taking their medication as prescribed | | (Abuelmagd et al., 2019; Baghikar et al., 2019; Njeru et al., 2015; Shultz et al., 2009) |
|  | Patients' barriers to exercise | - A sedentary lifestyle: no more walking - Financial challenges - Work responsibilities - Inclement weather - A lack of motivation - Physical limitations - Cultural traditions or norms - Family responsibilities, particularly among women | (Abuelmagd et al., 2019; Coffman et al., 2013; Kindarara et al., 2017; Magny-Normilus et al., 2020; Njeru et al., 2015; Renfrew et al., 2013) |  | Familiarity and habits associated with T2D self-management | - Regular blood glucose measurements - Moderate changes in diet and eating habits. - Weight control - Adapting to circumstances - Incorporating exercise into their everyday lives - Staying positive | | (Abuelmagd et al., 2019; Carolan-Olah & Cassar, 2018; Kindarara et al., 2017; Njeru et al., 2015) |
|  | Burden of self-management | - Loss of self-control - Feelings of vulnerability - Anxiety and diabetes stress - The physical pain associated with glucose testing or insulin injections. - Complexity of disease | (Joo & Lee, 2016; McConatha et al., 2020; Mitchell-Brown et al., 2017; Njeru et al., 2015; Roth et al., 2022; Smith-Miller et al., 2017) |  | Self-discipline | - Being disciplined: having a routine, changing habits - Self-efficacy | | (Mwalui, 2017; Njeru et al., 2015) |
|  | Struggling to follow dietary recommendations | - Food preferences: personal choice/taste; ethnic diet - Inadequate finances: price and thrift - Time for buying and preparing to prepare healthier meals. - Lack of fresh produce. - Habits difficult to break. - Having an absence of culturally sensitive diabetic dietary guidelines - Difficult in following dietary recommendations in social gatherings. - Conforming to traditional gender role expectations - Faced challenges finding their interests in local foods. - Place of consumption of main meals - Food cravings - Confusion with nutrition | (Abuelmagd et al., 2019; Carolan-Olah & Cassar, 2018; Cha et al., 2012; Deng & Chan, 2019; Kaltman et al., 2015; Kindarara et al., 2017; Leake, 2003; Mwalui, 2017; Njeru et al., 2015; Piombo et al., 2020; Roth et al., 2022; Shultz et al., 2009; Wang et al., 2012) | **Dietary self-management** | Nutritional profile | - Nutritional counselling and the choice of personalized diets | | (Carolan-Olah & Cassar, 2018; Deng & Chan, 2019; Kindarara et al., 2017; Piombo et al., 2020; Wieland et al., 2017) |
|  |  |  |  |  | A typical diet from the origin country | - Eating the same foods as previously but in lesser quantities - Eating more regularly - Drinking more water | | (Carolan-Olah & Cassar, 2018; Deng & Chan, 2019; Kindarara et al., 2017; Piombo et al., 2020; Wieland et al., 2017) |
|  |  |  |  |  | Health worries and concerns | - Want to be healthy and feel good. - Fear of complications - “I don’t want to get worse” | | (Deng & Chan, 2019; Njeru et al., 2015; Shultz et al., 2009) |
|  |  |  |  |  | Social support | - Social support from family and friends - Support from transcultural mediators: the involvement of social and health workers of the same language | | (Carolan-Olah & Cassar, 2018; Deng & Chan, 2019; Kindarara et al., 2017; Piombo et al., 2020; Wieland et al., 2017) |
| **Interpersonal barriers** | Lack of social support | - The expectations of familial and social obligations - Competing family needs - Lack of social support from family, friends, and communities - Social isolation from their neighbours of different races or ethnicity | (Abuelmagd et al., 2019; Hawkins et al., 2015; Magny-Normilus et al., 2021; McConatha et al., 2020; Nam et al., 2013; Roth et al., 2022; Smith-Miller et al., 2017; Wieland et al., 2017) | **Social support networks** | Family and friends support | - Strong support from family and friends. - Encouragement by family members | | (Barbara & Krass, 2013; Cha et al., 2012; Choi et al., 2015; Chun et al., 2011; Deng & Chan, 2019; Hawkins et al., 2015; Joo & Lee, 2016; Kaltman et al., 2015; Kindarara et al., 2017; Magny-Normilus et al., 2021; Mitchell-Brown et al., 2017; Mwalui, 2017; Njeru et al., 2015; Pettersson et al., 2023; Wieland et al., 2017) |
|  |  |  |  |  | Peer or community support | - Interaction with community resources: e.g., creating and sustaining strong networks within their Haitian communities. - Support from the Chinese Community Centre | | (Chun et al., 2011; Kindarara et al., 2017; Mitchell-Brown et al., 2017) |
|  | Quality of provider-patient relationship | - Poor communication with providers - Negative interactions and communications with healthcare providers/other personnel | (Baghikar et al., 2019; Fagerli et al., 2005; Hawkins et al., 2015; Smith-Miller et al., 2017) |  | Patient-provider relationships | - Healthcare providers’ non-judgmental recognition of alternative medicine practices. - Healthcare providers’ feedback. - Community health worker: emotional, appraisal, and informational support. - Trust in the GP's medical expertise and social support | | (Cha et al., 2012; Deng & Chan, 2019; Hawkins et al., 2015; Kindarara et al., 2017; Magny-Normilus et al., 2021; Pettersson et al., 2023) |
| **Societal barriers** | High cost of T2D care | - Dealing with costs not covered by insurance | (Baghikar et al., 2019; Hawkins et al., 2015; Joo & Lee, 2016; Magny-Normilus et al., 2021; Smith-Miller et al., 2017) | **Financial**  **independence** | Financial independence | - Financial security and independence | | (Barbara & Krass, 2013) |
|  | Financial hardship | - Low household income - Limited ability to buy medication and management supplies. - The cost of ongoing medical management (basic day-to-day needs over medications and doctor appointments) - Low socioeconomic status | (Kaltman et al., 2015; Kindarara et al., 2017; Leake, 2003; Magny-Normilus et al., 2021; Wieland et al., 2017) |  |  |  | |  |
|  | Work problems | - A busy work schedule. - Work was a competing priority, particularly for employed men | (Hawkins et al., 2015; Leake, 2003; Wieland et al., 2017) |  |  |  | |  |
| **Linguistic and cultural barriers** | Language barriers | - Limited English language proficiency, especially among the elderly - A lack of Chinese language materials | (Carolan-Olah & Cassar, 2018; Deng & Chan, 2019; Joo & Lee, 2016; Jowsey et al., 2011; Kaltman et al., 2015; Mitchell-Brown et al., 2017; Mwalui, 2017; Nam et al., 2013; Roth et al., 2022; Wang et al., 2012) | **Language and cultural supports** | Language | - Clinicians or translators from their community - Translated diabetes education sessions | | (Shah et al., 2022) |
|  | Cultural beliefs, attitudes & influences | - Cultural disconnection - Acculturative stress - Being unaware of the disease/unfamiliar with disease prevention - The belief of only seeking health care when ill - Cultural characteristics: e.g., alcohol, parties, rice, soda, sweets - Cultural relevancy: Lack of access to familiar treatment options like Traditional Chinese Medicine - Traditional family values | (Barbara & Krass, 2013; Deng & Chan, 2019; Kindarara et al., 2017; Leake, 2003; McConatha et al., 2020; Mwalui, 2017; Shah et al., 2022) |  | Cultural beliefs and attitudes | - Involvement in religion and adopting traditional remedies - Culturally specific education/Cultural support initiatives - A compelling feeling for self-care to be healthy enough to care for family - Faith & Spirituality: Faith made self-care work easier for some | | (Barko et al., 2011; Jager et al., 2019; Kindarara et al., 2017; Mitchell-Brown et al., 2017; Njeru et al., 2015; Wieland et al., 2017) |
|  | Life after immigration | - Differences in way of life and environment between the two countries | (Hawkins et al., 2015; Kindarara et al., 2017; Magny-Normilus et al., 2020; Nam et al., 2013) |  | Acculturation levels | - Lifestyle changes after immigration - Ease of diabetes management after immigration | | (Alzubaidi et al., 2015; Shah et al., 2022; Venkatesh et al., 2013) |
| **Resource barriers** | Access to health services | - Inadequate diabetes education - Health insurance challenges - Limited community-level healthcare resources - Waiting until symptoms get severe. - A lack of information: e.g., where to go, Chinese-speaking community - Limited resources for diabetes self-management, including medications and blood glucose testing materials. - Difficulties finding a doctor of the same sex. - Dissatisfaction with healthcare services: e.g., long waits to see doctors or specialists; a perceived lack of cultural competence among healthcare practitioners | (Barbara & Krass, 2013; Barko et al., 2011; Cha et al., 2012; Hawkins et al., 2015; Joo & Lee, 2016; Jowsey et al., 2011; Kindarara et al., 2017; Leake, 2003; Leung et al., 2014; Magny-Normilus et al., 2020; Renfrew et al., 2013) | **Available resources** | Use of media | - Use of media (printed or online) to get information - Use of mobile phone | | (Carolan-Olah & Cassar, 2018; Deng & Chan, 2019; Kindarara et al., 2017; Piombo et al., 2020; Wieland et al., 2017) |
|  | Lack of time | - Insufficient time to manage daily commitments related to work and childcare issues and follow an exercise program. - Lack of time: diet and exercise | (Cha et al., 2012; Joo & Lee, 2016; Kaltman et al., 2015; Kindarara et al., 2017) |  | Flexible scheduling | - Flexible scheduling that accounts for participants’ jobs and family obligations | | (Joo & Lee, 2016; Kaltman et al., 2015; Shah et al., 2022) |
|  | Transportation issues | - Limited access to transportation: “a barrier to see a doctor”. | (Cha et al., 2012; Hawkins et al., 2015; Kindarara et al., 2017; Leung et al., 2014) |  |  |  |  | |

Notes: T2D, type 2 diabetes; GP, general practitioner
