# Supplementary material for: Cultural Influences, Experiences and Interventions Targeting Self‐Management Behaviours for Prediabetes or Type 2 Diabetes in First‐Generation Immigrants: A Scoping Review
Source: J Adv Nurs. 2024 Nov 21;81(6):2929–45. doi: 10.1111/jan.16621 (PMC12080094; doi:10.1111/jan.16621)
Supplement: Supplementary file 6 — Table S5. [file JAN-81-2929-s005.docx]

Supplementary table 5. Key outcome measurement tools

| **Outcome Indicators** | **Example of tools** | **Studies** |
| --- | --- | --- |
| Diabetes knowledge | Diabetes Knowledge Test (DKT) | n=9 |
|  | The Spoken Knowledge in Low Literacy in Diabetes (SKILLD) scale | n=2 |
|  | Diabetes Knowledge Questionnaire (DKQ) | n=2 |
|  | Diabetes Educational Profile | n=1 |
|  | ADA risk assessment questions | n=1 |
| Health literacy | Short Test of Functional Health Literacy in Adults (S-TOFHLA) | n=4 |
|  | Short Assessment of Health Literacy for Spanish-Speaking Adults (SAHLSA) | n=1 |
|  | Short Form of the Korean Health Literacy scale. | n=1 |
|  | The Rapid Estimate of Adult Literacy in Medicine (REALM) | n=1 |
| Self-efficacy | Diabetes Self-Efficacy Scale | n=6 |
|  | Stanford Self-Efficacy for Diabetes scale | n=3 |
|  | Stanford Chronic Disease Self-Efficacy scale | n=3 |
|  | Bandura Self-Efficacy Scale | n=2 |
|  | Diabetes management self-efficacy (DMSE) scale | n=1 |
|  | Self-Efficacy for Exercise Behaviours Scale (SEEBS) | n=1 |
|  | Lorig’s Diabetes Self-Efficacy Scale | n=1 |
| Empowerment | Diabetes Empowerment Scale (DES) | n=1 |
| Quality of life | Diabetes Quality of Life Measure (DQOL) | n=5 |
|  | 12-item Short-form Health Survey (SF-12) | n=2 |
|  | The Centers for Disease  Control and Prevention Health-Related Quality-of-Life 14-item Measure (CDC HRQOL-14) | n=1 |
| Self-rated health | WHO-5 Well-being index | n=2 |
|  | Self-reported health | n=2 |
|  | EQ VAS questionnaires | n=1 |
|  | Well-being: Cantril’s ladder | n=1 |
| Illness perceptions | Brief Illness Perception Questionnaire (IPQ) | n=2 |
|  | Moss-Morris and colleagues’ revised Illness Perception Questionnaire (IPQ-R) | n=1 |
| Diabetes-related experiences/symptoms | Diabetes Symptom Self-care Inventory (DSSCI) | n=1 |
|  | Fatigue Assessment Scale (FAS) | n=1 |
| Diabetes distress | The Diabetes Distress Scale (DDS) | n=5 |
|  | Problem Areas in Diabetes Scale (PAID) | n=3 |
|  | The Diabetes Distress Disclosure Index (DDDI) | n=1 |
| Depression | Center for Epidemiological Studies Depression Scale (CES-D) | n=5 |
|  | Patient Health Questionnaire-9(PHQ-9) | n=3 |
|  | The Kim Depression Scale for Korean Americans (KDSKA) | n=1 |
| Anxiety | Generalized Anxiety Disorder (GAD) | n=2 |
| Psychosocial adjustment | Psychosocial Adjustment to Illness Scale-Self Report (PAIS-SR) | n=1 |
| Self-management skills | Summary of Diabetes Self-care Activities (SDSCA) | n=14 |
|  | The SDSCA-R&E | n=2 |
|  | Diabetes Care Profile | n=2 |
|  | Diabetes self-management (DSM) Composite Scale | n=1 |
|  | AADE7 self-care behaviours: Association of Diabetes Educators Questionnaire Score | n=1 |
| Dietary behaviors | The 8-item Starting the Conversation scale | n=1 |
|  | A simplified food-frequency questionnaire (FFQ) | n=1 |
|  | A culturally sensitive adaption of the Mediterranean diet score | n=1 |
| Medication adherence | Medication adherence scores (MMAS-8) | n=5 |
|  | The Hill-Bone medication compliance scale | n=1 |
| Physical activities | 7-day Physical Activity Recall (7-Day PAR) questionnaire | n=2 |
|  | International Physical Activity Questionnaire | n=1 |
| Behavioural risk factor | The Behavioral Risk Factor Surveillance Survey (BRFSS) | n=2 |
|  | Lifestyle-related diabetes risk factors | n=1 |
| Acculturation levels | Suinn-Lew Asian self-identity acculturation instrument (SL-ASIA) | n=3 |
|  | Short Acculturation Scale for Hispanics (SASH) | n=3 |
|  | Bicultural efficacy for health management scale | n=2 |
|  | Acculturation scale for the Hispanic linguistic proficiency subscale | n=1 |
|  | 15-item Armenian ethnic orientation questionnaire-revised (AEOQ-R) | n=1 |
|  | 12-item Acculturation Inventory  Short acculturation and language screening scale | n=1  n=1 |
| Traditional beliefs | Chinese-Western Medical Beliefs (CWMB) Scale | n=1 |
|  | Chinese Health Beliefs (CHB) Scale | n=1 |
| Family support | Diabetes Family Behavior Checklist-II (DFBC) | n=2 |
|  | Medical Outcomes Study (MOS) Social Support Inventory | n=1 |
|  | Family or friend social support (FSS) questionnaire | n=1 |
|  | Family APGAR questionnaire | n=1 |
|  | Quality of marriage index | n=1 |
| Healthcare use | Immigrant Use of Health Care Scale | n=1 |
|  | The consumer assessments of healthcare providers and systems' cultural competency item set (CAHPS-CC) | n=1 |
| Program Satisfaction | Patient Satisfaction with Cancer-Care (PSCC) scale | n=1 |
