# Supplementary material for: Cultural Influences, Experiences and Interventions Targeting Self‐Management Behaviours for Prediabetes or Type 2 Diabetes in First‐Generation Immigrants: A Scoping Review
Source: J Adv Nurs. 2024 Nov 21;81(6):2929–45. doi: 10.1111/jan.16621 (PMC12080094; doi:10.1111/jan.16621)
Supplement: Supplementary file 7 — Appendix S1. [file JAN-81-2929-s004.docx]

**Appendix: Data extraction form**

| **Scoping Review Details** | |
| --- | --- |
| Scoping Review title: Cultural influences, experiences, and interventions targeting self-management behaviours for prediabetes or type 2 diabetes in first-generation immigrants | |
| Review objective/s: To map the key concepts and identify gaps in knowledge on prediabetes or type 2 diabetes self-management behaviours among first-generation immigrants | |
| Review question/s: What is the existing evidence regarding self-management behaviours among first-generation immigrants with prediabetes or type 2 diabetes? | |
| **Inclusion/Exclusion Criteria** | |
| Population: Adults aged 18 years and over who identified as first-generation immigrants and had been diagnosed with prediabetes or type 2 diabetes. | |
| Concept: Self-management | |
| Context: In any country or healthcare setting | |
| Types of evidence source: All types of literature with qualitative, quantitative, and mixed methods study designs | |
| **Evidence Source Details and Characteristics** | |
| Citation details (e.g. author/s, year, title, journal, volume, issue, pages) | |
| Study design |  |
| Sampling method |  |
| Study objective/aims |  |
| Theoretical framework (if applicable) |  |
| **Participants** |  |
| Number, age, sex, diagnosis, condition duration |  |
| Country of origin |  |
| Primary language (spoken by participants) |  |
| Secondary language and proficiency (e.g., English proficiency) |  |
| **Concept (Self-management)** |  |
| Content (eg, diet, exercise, education, medication) |  |
| Duration (length and number of sessions) |  |
| Delivery format (eg, face-to-face, online, one-on-one, group) |  |
| Length of follow-up |  |
| **Context** |  |
| Race/ethnicity and percentage |  |
| Country of residence |  |
| Duration of immigration |  |
| Location/Settings (e.g., home, primary care, community, hospital) |  |
| **Details/Results Extracted from Source of Evidence** | |
| Primary outcomes and measures | |
| Barriers to engagement with self-management |  |
| Facilitators of engagement with self-management |  |
| Main findings |  |
| Does the study describe cultural influences or culturally tailored strategies for self-management? If yes, what are these? |  |
| **Data Items** |  |
| Data collection method |  |
| Data collection process |  |
| Approach to analysis |  |
| **Limitations and Implications** |  |
| Limitations identified by the author(s) of the study |  |
| Implications identified by the author(s) of the study |  |
